# A scoping review and meta-analysis on the prevalence of pan-tumour biomarkers (dMMR, MSI, high TMB) in different solid tumours

**DOI:** 10.1038/s41598-022-23319-1

**Published:** 2022-11-28

**Authors:** Yoon-Jung Kang, Sophie O’Haire, Fanny Franchini, Maarten IJzerman, John Zalcberg, Finlay Macrae, Karen Canfell, Julia Steinberg

**Affiliations:** 1 The Daffodil Centre, The University of Sydney, a Joint Venture with Cancer Council NSW, Sydney, NSW 2060 Australia; 2 Cancer Health Services Research, Centre for Health Policy, Melbourne School of Population and Global Health, Faculty of Medicine, Dentistry and Health Sciences, The University of Melbourne, Parkville, VIC 3053 Australia; 3 Sir Peter MacCallum Department of Oncology, Faculty of Medicine, Dentistry and Health Sciences, The University of Melbourne, Melbourne, VIC 3010 Australia; 4 Department of Medical Oncology, Alfred Health and School of Public Health and Preventive Medicine, Faculty of Medicine, Monash University, Melbourne, VIC 3004 Australia; 5 Colorectal Medicine and Genetics, and Department of Medicine, University of Melbourne, The Royal Melbourne Hospital, Parkville, VIC 3050 Australia

**Keywords:** Prognostic markers, Cancer, Health policy, Cancer immunotherapy

## Abstract

Immune checkpoint inhibitors have been approved in the USA for tumours exhibiting mismatch repair deficiency (dMMR), microsatellite instability (MSI), or high tumour mutational burden (TMB), with regulatory and reimbursement applications in multiple other countries underway. As the estimated budget impacts of future reimbursements depend on the size of the potential target population, we performed a scoping review and meta-analysis of the prevalence of these pan-tumour biomarkers in different cancers. We systematically searched Medline/Embase and included studies reporting the prevalence of dMMR/MSI/high TMB in solid tumours published 01/01/2018–31/01/2021. Meta-analyses were performed separately for the pan-cancer prevalence of each biomarker, and by cancer type and stage where possible. The searches identified 3890 papers, with 433 prevalence estimates for 32 different cancer types from 201 studies included in meta-analyses. The pooled overall prevalence of dMMR, MSI and high TMB (≥ 10 mutations/Mb) in pan-cancer studies was 2.9%, 2.7% and 14.0%, respectively. The prevalence profiles of dMMR/MSI and high TMB differed across cancer types. For example, endometrial, colorectal, small bowel and gastric cancers showed high prevalence of both dMMR and MSI (range: 8.7–26.8% and 8.5–21.9%, respectively) and high TMB (range: 8.5–43.0%), while cervical, esophageal, bladder/urothelial, lung and skin cancers showed low prevalence of dMMR and MSI (< 5%), but high prevalence of high TMB (range: 23.7–52.6%). For other cancer types, prevalence of all three biomarkers was generally low (< 5%). This structured review of dMMR/MSI/high TMB prevalence across cancers and for specific cancer types and stages provide timely evidence to inform budget impact forecasts in health technology assessments for drug approvals based on these pan-tumour biomarkers.

## Introduction

With increasing application of genomic medicine, cancer treatment has started to evolve from an approach based on tumour location to targeted treatments based on specific molecular characteristics (“biomarkers”) of the tumour^1^. In particular, many research efforts focus on the identification of so-called “pan-tumour biomarkers”, which can predict favourable response to a treatment for cancers originating from any tumour site. The first drug to receive tumour-agnostic approval based on presence of a pan-tumour biomarker was pembrolizumab (Merck & Co., Inc.), an immune checkpoint inhibitor targeting and blocking PD-1, which the US Food and Drug Administration approved for use for the treatment of adult and paediatric patients with unresectable or metastatic solid tumours exhibiting mismatch repair deficiency (dMMR) or microsatellite instability (MSI)^2^. This approval was later broadened to another biomarker, high tumour mutational burden (TMB)^3^. Similar pan-cancer regulatory approvals were made or are under consideration in other jurisdictions, including the European Union, Japan, and Australia (in addition to some cancer-specific approvals for e.g. colorectal cancer that are already in place)^4–6^. Clinical trials for multiple other drugs targeting these pan-tumour biomarkers are also in progress^7^. dMMR, MSI and TMB are distinct but related biomarkers; the hypermutation generally concomitant with all three is associated with improved immune checkpoint inhibitor response, thus linking the three biomarkers to these targeted treatments^8^.

Given the relatively high cost of pembrolizumab and other potential targeted treatments^9^, a key question to inform health system planning and budget impact evaluations is how many patients might be eligible for these treatments based on the presence of these biomarkers. In particular, budget impact evaluations are an integral aspect of health technology assessments that summarise the information needed to inform policy and funding decisions (including e.g. drug efficacy, effectiveness, cost-effectiveness, and re-imbursement costs)^10^. To facilitate such assessments, it is therefore crucial to map and consolidate the recent available evidence on the prevalence of the pan-tumour biomarkers, where possible, by cancer type as well as across all cancers. As approvals based on dMMR/MSI/high TMB currently focus on patients with advanced-stage disease, and biomarker prevalence may vary between cancer stages^11^, stage-specific prevalence estimates are also important where data are available.

Past reviews have typically focused on a single cancer type, with only two existing structured/systematic reviews consolidating evidence for multiple different cancers. One review included literature published to October 2017 and focused on the prevalence of dMMR and MSI only^12^. Another review included literature published to September 2018 and focused on the prevalence of MSI, and separately, the overlap between MSI and high TMB based on studies that assayed both (but without separate consideration of the prevalence of high TMB)^13^. Thus, consolidation of more recent expanding evidence across cancers with prevalence estimates for all three biomarkers is required to inform comprehensive health technology assessments. To address the evidence gaps and facilitate health technology assessments and health system planning, the aim of this scoping review was to identify the available evidence on the prevalence of each of dMMR, MSI and high TMB in adult and paediatric solid tumours, by cancer type and cancer stage. Scoping reviews follow a structured process similar to systematic reviews; however, their general purpose is to identify and map the available evidence, thus they generally do not involve an assessment of risk of bias^14^. Our specific aims were to: (1) provide a broader overview of studies reporting the prevalence of these three pan-tumour biomarkers; and (2) consolidate the evidence by cancer type and cancer stage. To the best of our knowledge, this is the first structured review on the prevalence of all three pan-tumour biomarkers (dMMR, MSI, high TMB) in a pan-cancer setting.

## Methods

This scoping review comprises two components. The first component is a broader overview of literature reporting the prevalence of dMMR/MSI/high TMB (for high TMB, based on appropriate TMB thresholds determined from the literature). Mismatch repair deficiency (dMMR) can be defined as loss of MLH1/MSH2/MSH6/PMS2 function determined as loss of immunohistochemistry staining or genetic loss of function identified in gene panels, whole exome, or whole genome sequencing. Microsatellite instability (MSI) is commonly defined as instability of 2 + microsatellite markers determined by PCR, although some studies have used other thresholds. MSI can also be determined based on large gene panels, whole exome or whole genome sequencing, using dedicated algorithms. Definitions of high tumour mutational burden (TMB) are more study-specific, with thresholds of e.g. ≥ 10 mutations per Mb or ≥ 20 mutations per Mb. TMB is also generally determined based on large gene panels, whole exome, or whole genome sequencing. The second component consolidates the evidence on the prevalence of these three pan-tumour biomarkers, applying a cancer-type-specific minimal sample size for studies to be included in meta-analyses as described below.

The protocol and the PRISMA-ScR checklist^13^ for scoping reviews are provided in Appendix I; the protocol was not registered on PROSPERO as that database does not accept scoping reviews and protocol registration is not mandatory for scoping reviews^14,15^.

### Search strategy

**Figure 1:**
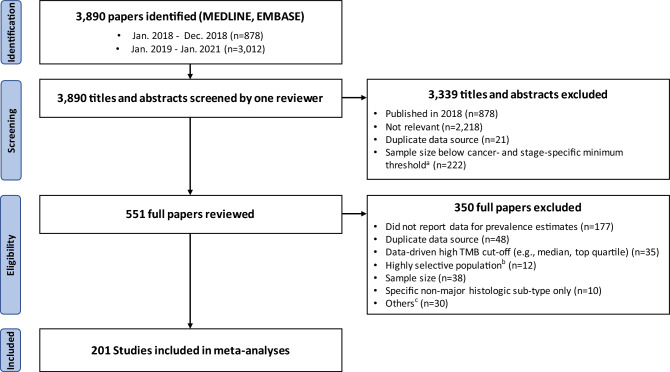
Scoping review process. ^a^We determined a minimum sample size threshold for each cancer type and stage to focus on estimates from larger studies where available, while retaining 10+ studies (see Supplementary Table S6 for details). ^b^Excluding studies focused on selected populations based on family history or inherited predisposition to cancer, rare histologic subtype(s) only, or specific molecular subtypes only. ^c^Other reasons for exclusion are unavailability of the full-text article (n = 10), focus on validation of a new assay (n = 8), use of blood rather than tumour samples (n = 8), and < 4 mismatch repair proteins evaluated by immunohistochemistry for colorectal cancer (n = 4).

Figure 1 illustrates the scoping review process, with search terms and inclusion/exclusion criteria detailed in Appendix I.2. A literature search was conducted on 01/02/2021 using Medline and Embase to identify articles that: (1) explicitly mention keywords related to cancer and to MMR, MSI, or TMB; (2) were published 01/01/2018–31/01/2021 (based on a trial search suggesting almost all studies published prior to 2018 were related to colorectal cancer); and (3) are in English. Conference abstract and duplicate records were removed.

### Selection criteria

One reviewer performed title/abstract screening (YJK), with two reviewers (SO, JS) double-screening 25% of articles (100% concordance after discussion). This scoping review focused on solid tumours only, excluding haematologic tumours and lymphomas (as per the current/proposed pembrolizumab indication in the USA, the European Union, Japan, and Australia^2–6^). We included original research studies, reviews (including structured or systematic reviews) and meta-analyses, with careful considerations to avoid data duplication in meta-analyses (see “Data extraction” below). Case studies and studies focused on selected populations based on family history or inherited predisposition to cancer, rare histologic sub-type(s) only, or specific molecular sub-types only, were excluded. In the broad literature overview, we included studies with cancer-specific sample size ≥ 50 or no number of cases listed in the abstract.

For the second component of this work, we imposed the following additional inclusion criteria as part of the full-text review. Only studies published since 2019 were included due to very few studies on TMB published prior to 2019. Moreover, as underpowered studies often contribute little information to meta-analysis, it has been suggested that exclusion of small studies could be appropriate for meta-analyses in a rapid review when at least two adequately powered studies are available^16^. Therefore, we determined a minimum sample size threshold for each cancer type and stage, to allow focus on estimates from larger studies where available, while retaining 10+ studies for each cancer type and stage (see Supplementary Table S6 for details). Full-text review was performed by one reviewer (YJK), with a second reviewer (JS) independently assessing 10% of studies (100% concordance after discussion).

### Data extraction

Details on data extraction are described in Appendix I.3. During the title/abstract screening for the broad literature overview, we extracted data on pan-tumour biomarker type (dMMR/MSI/TMB), cancer type, cancer stage (early, advanced, not limited to specific stage; see Supplementary Table S5 for detailed definitions) and sample size (50–99, 100–199, 200–399, 400–999, 1000+, unspecified).

For all studies included after full-text review (after imposing minimum sample size thresholds, see “Selection criteria” above), we extracted data on key study characteristics including study population, study design, major data sources, cancer type, cancer stage, assays used (see Supplementary Table S3 for details) and the prevalence of dMMR/MSI/high TMB (and the TMB threshold). Cancer types were grouped into 13 tumour group categories by body location or system (Supplementary Table S4). We considered data on biomarker prevalence for early-stage cancers and separately advanced-stage cancers, as well as overall prevalence not restricted to specific stages. Pan-cancer biomarker prevalence estimates were consolidated based on studies that included ≥ 10 cancer types and did not focus on selected cancer types only (e.g. only rare cancers). Overlap in data from underlying primary studies was considered carefully to prevent data duplication in meta-analyses (see Appendix II for managing overlap of data sources and the list of the studies and the rationale for inclusion/exclusion in meta-analyses). We considered the prevalence estimates as referring to the biomarker that was assayed as per each study’s methods sections.

Data extraction was performed in duplicate (by YJK, JS) for 10% of studies to verify high concordance (100%), then completed by one reviewer (YJK) for the remaining studies.

### Quality assessment and risk of bias

Critical appraisal of included studies is not required for scoping reviews^14,15^, and was not performed in this study (with potential impact elaborated in the “Discussion” section).

### Synthesis of results

#### Broad overview of the literature

For an overview of the current landscape of studies reporting the prevalence of dMMR/MSI/high TMB, we created a graphical summary of the characteristics of studies reporting the prevalence of these three pan-tumour biomarkers in adult solid tumours: publication year (2018, 2019, 2020–Jan. 2021), study size, and included cancer type(s) and stage(s).

#### Meta-analyses of biomarker prevalence

As only one study reported the prevalence in paediatric tumours^17^, meta-analyses focused on adult solid tumours only and the prevalence in paediatric tumours is not presented in this review. The primary outcome measures were the overall proportions of cancer cases with each of (1) dMMR, (2) MSI and (3) high TMB (at different TMB thresholds), among all cancer cases whose tumour samples were evaluated for each of the pan-tumour biomarkers. We generally separated dMMR and MSI where possible since most studies included in our analysis focused on either dMMR or MSI alone, thus we wanted to retain that more detailed information. Notably, the two biomarkers may be discordant in some cases (with e.g. 4.9% discordance reported by a previous study on endometrial cancers)^18^. However, as some previous work did not distinguish between dMMR and MSI for colorectal cancers, we also carried out a meta-analysis of prevalence estimates for colorectal, colon, or rectal cancer based on studies that combined results for dMMR and MSI. These analyses were carried out on three levels: (1) cancer-specific analyses of overall prevalence; (2) analyses of overall prevalence by tumour group (using studies that only reported prevalence on this level, e.g. for gastrointestinal cancers); and (3) analyses of pan-cancer overall prevalence obtained from pan-cancer studies. Secondary outcome measures were the prevalence of (1) dMMR, (2) MSI, (3) high TMB among early-stage cancers, and separately, among advanced-stage cancers, and the analyses were performed on three levels (if available) analogous to the main analyses. Sub-group analyses were performed of: (1) prevalence by cancer sub-types (distinct histologic sub-types of a specific cancer type, e.g. separate analyses for non-small cell lung cancer and small cell lung cancer, where data were available); and (2) pooled prevalence of dMMR and MSI in colorectal, colon and rectal cancers (given high concordance between dMMR and MSI in these cancer types^19^). The specific tumour groups, cancer types and cancer sub-types considered in the meta-analyses are listed in Supplementary Table S4.

For cancer-type-specific estimates, we only considered biomarker prevalence estimates based on a number of samples above the cancer-specific minimum sample size threshold (see Supplementary Table S6). Meta-analyses were not possible for estimates based on a high TMB threshold used by only one study (e.g., ≥ 16 mutations/Mb^20^ or ≥ 17 mutations/Mb^21^).

We performed random-effect meta-analyses, using the inverse variance heterogeneity model to pool the Freeman-Tukey transformed proportions of cases with dMMR, MSI, or high TMB^22^. Heterogeneity across studies (for meta-analyses with ≥ 2 estimates) was presented based on the I^2^ score estimate, with higher I^2^ score indicating higher level of heterogeneity, and based on the heterogeneity test p-value (defining significance at p < 0.05). All statistical analyses were performed using R (Version 4.1.1) and the package “meta” (version 4.19-1).

Sensitivity analyses of the prevalence of dMMR/MSI/high TMB were pre-planned for each cancer type by (1) assay used (where reported); (2) in tumours that progressed following prior systemic treatment; if ≥ 3 estimates were available for a given cancer type and each assay/treatment category. Therefore, these analyses were only performed for the overall prevalence of MSI in colorectal and gastric cancers (PCR *vs* gene panel sequencing), and for high TMB (≥ 10 mutations/Mb) in advanced lung cancer (gene panel sequencing *vs* whole exome sequencing).

We have investigated potential publication bias via funnel plots, focusing on meta-analyses of ≥ 10 estimates based on the recommendations from the Cochrane Handbook for Systematic Reviews of Interventions^23^. Therefore, publication bias was assessed for 4 analyses: the overall prevalence of dMMR in colorectal cancer, the overall prevalence of MSI in colorectal cancer, the overall prevalence of MSI in gastric cancer, and the prevalence of MSI in early-stage gastric cancer. For the analysis of MSI prevalence in early-stage gastric cancer, the funnel plot was also repeated after exclusion of two outlier estimates (based on a different assay to the other nine studies).

## Results

### Search results

The search yielded 3,890 papers published 01/01/2018–31/01/2021. A total of 962 papers satisfied the initial criteria and were included in the literature overview. Applying cancer-type-and stage-specific minimum sample size thresholds, we reviewed full texts for 551 papers and included estimates from 201 studies in meta-analyses (Fig. 1). Of the 201 included studies, the majority were retrospective analyses (n = 160), followed by clinical trials (n = 14), prospective studies (n = 13), reviews (including structured and systematic review) or meta-analyses (n = 11), with 3 other studies (case–control study, prospective series/clinical trial, and a longitudinal data linkage study). There were similar numbers of European (n = 56), Asian (n = 53), North American (n = 42) and global studies (n = 40) with smaller number of studies in other parts of the world (7 from Oceania, 2 from the Middle East and 1 from South America). A total of 32 studies that analysed major common data sources (The Cancer Genome Atlas, Foundation Medicine Database, Memorial Sloan Kettering Cancer Center data, Dana-Farber Cancer Institute data, and Caris Life Science data) were included in the analysis, comprising 55% of all samples (447,128/807,1360).

### Overview of literature reporting the prevalence of dMMR/MSI/high TMB

**Figure 2:**
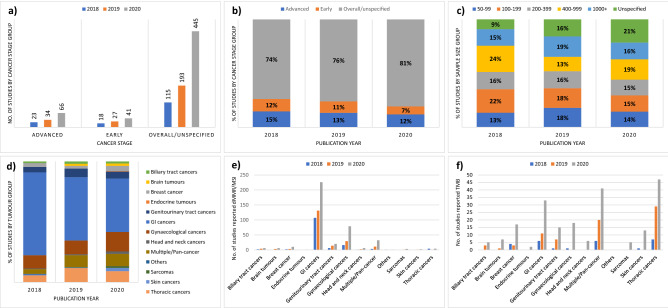
Landscape of studies reporting the prevalence of dMMR/MSI/high TMB. (**a**) Number of studies by cancer stage; (**b**) the relative proportion of studies by cancer stage; (**c**) the relative proportion of studies by study sample size; (**d**) the relative proportion of studies by tumour group; (**e**) number of studies reporting the prevalence of dMMR/MSI; and (**f**) number of studies reporting the prevalence of high TMB. *dMMR* mismatch repair deficiency; *MSI* microsatellite instability; *TMB* tumour mutational burden.

Between 01/01/2018 and 31/01/2021, the number of studies reporting the prevalence of dMMR/MSI/high TMB almost doubled annually, but there has been no substantial change in the relative proportion of studies by cancer stage and study sample size (Fig. 2). With respect to the cancer types different studies focused on, a notable change was the decrease in the relative proportion of studies reporting on gastrointestinal cancers, from 69% of all studies reporting prevalence of dMMR/MSI/high TMB in 2018 to 44% in 2020/2021. By contrast, there has been a substantial increase in the relative proportion of studies focusing on the prevalence of high TMB, from 9% of all studies in 2018 to 28% in 2020/2021. The relative proportion of studies reporting the prevalence of high TMB has also increased for most tumour groups, e.g. from 14% in 2018 to 43% in 2020/2021 for genitourinary tract cancers, and from 64% in 2018 to 92% in 2020/2021 for thoracic cancers (Supplementary Fig. S1).

### Meta-analyses of the prevalence of dMMR/MSI/high TMB

A total of 433 estimates of the prevalence of dMMR, MSI, or high TMB were obtained on three levels: 412 estimates for specific cancer types (overall or stage-specific), available for 32 cancer types across 13 tumours groups; 8 tumour-group-specific estimates; and 13 pan-cancer estimates. Of the 412 estimates for specific cancer types, 70% were for gastrointestinal cancers (n = 170), breast and gynaecological cancers (n = 69), and genitourinary tract cancers (n = 51). Almost 2/3 of the estimates were for overall prevalence across cancer stages (269/412, 65%). The number of estimates for prevalence of high TMB (n = 108) was slightly less than for dMMR (n = 135) and MSI (n = 160), with only three records related to early-stage cancers (Supplementary Tables S7 and S8). dMMR was mostly assayed by IHC except in three studies, which used whole-exome sequencing or a gene panel test^24–26^. MSI was mostly assayed by PCR or gene panel sequencing, and TMB was mostly assayed by gene panel sequencing (Supplementary Data and Supplementary Table S9). For the included studies, an extract of key information on the cancer type and subtype, the assay used, the specific definition for the biomarker (e.g. the threshold used for high TMB), the total number of cases, and the number of cases exhibiting the pan-tumour biomarker is provided in Supplementary Data.

Table 1 shows the pooled overall prevalence of dMMR, MSI and high TMB (≥ 10 mutations/Mb) based on pan-cancer studies, while Tables 2, 3 and 4 show cancer type- and stage-specific prevalence. Additional results are shown in Supplementary Table S19 (meta-analyses of studies that reported prevalence on tumour group level only) and Supplementary Table S20 (meta-analyses of prevalence estimates for colorectal, colon, and rectal cancers based on studies that combined results for dMMR and MSI).

**Table 1:** Overall pan-cancer prevalence of dMMR/MSI/high TMB in adult solid tumours.

Pan-tumour biomarker (cut-off)	Overall prevalence from random-effect model (95% CI)^a^
dMMR^24,37^	2.9% (2.7–3.1%), I^2^ = 0
MSI^17,18,21,37,38^	2.7% (2.1–3.4%), I^2^ = 0.98^b^
High TMB (≥ 10 mut/Mb)^38,39^	14.0% (3.9–28.8%), I^2^ = 1^b^
High TMB (≥ 17 mut/Mb)^21^	8.4% (8.1–8.7%)
High TMB (≥ 20 mut/Mb)^17,40^	6.6% (4.4–9.1%), I^2^ = 0.98^b^

*dMMR* mismatch repair deficiency, *MSI* microsatellite instability, *TMB* tumour mutational burden, *CI* confidence interval.

^a^Estimates for pan-cancer biomarker prevalence were considered based on studies that included ≥ 10 solid cancer types, excluding haematologic tumours and lymphomas. Heterogeneity across studies was presented based on the point estimate of I^2^ score and heterogeneity test, if ≥ 2 records were available for each pan-tumour biomarker.

^b^Statistically significant heterogeneity across studies (p < 0.05).

Another paper (Shemesh et al.^20^) reported the prevalence of high TMB (≥ 16 mut/Mb) based on the analysis of seven studies evaluating the efficacy and safety of atezolizumab monotherapy. As four of the seven studies included locally advanced or metastatic cancers only, contributing 1671/2894 individuals to the pooled analysis, the pooled estimate (17.7%, 95% CI 15.4–20.2%) may not be directly comparable with other non-stage-specific estimates and was not included in the table for overall prevalence.

#### Pan-cancer prevalence of dMMR/MSI/high TMB

The pooled overall prevalence of dMMR and MSI in pan-cancer studies was lower than the prevalence of high TMB (≥ 10 mutations/Mb) at 2.9%, 2.7% and 14.0%, respectively. No pan-cancer studies reporting stage-specific prevalence of dMMR/MSI/high TMB were identified (Table 1).

#### Prevalence of dMMR

The pooled overall prevalence of dMMR was high for endometrial (26.8%), small bowel (21.0%), colorectal (11.7%), colon (8.9%) and gastric (8.7%) cancers. However, here and for all following results for all three biomarkers, most estimates had wide 95% confidence intervals (CIs), see Tables 2, 3 and 4. The pooled overall prevalence was generally low (~ 5%) for gynaecological cancers other than endometrial cancer and for other cancer types. Substantial variation was found in the pooled overall prevalence of dMMR among gastrointestinal cancers (range: 0.0–21.0%). Where stage-specific data were available, dMMR tumours tended to be identified more often in early-stage disease as seen in colon, colorectal and gastric cancers (Table 2). Substantial variation in the pooled overall prevalence of dMMR was also found by cancer sub-type where data were available, including: (1) ovarian cancer (11.8% in endometroid/non-serous/mucinous carcinoma *vs* 2.4% in any ovarian carcinoma); (2) head and neck cancer (7.4% in oral cavity carcinoma *vs* 0.0% in salivary gland carcinoma); and (3) skin cancer (6.7% in melanoma *vs* 2.0% in non-melanoma), see Supplementary Table S15.

**Table 2:** Prevalence of mismatch repair deficiency in adult solid tumours by cancer type and stage.

Cancer	Prevalence from random-effect model (95% CI)^a^		
	Overall	Early stage	Advanced stage
**Gastrointestinal cancers**			
Anal cancer	–	–	–
Appendiceal cancer	2.5% (0.0–7.4%)	–	–
Colon cancer^c^	8.9% (5.4–13.2%), I^2^ = 0.81^b^	20.7% (14.3–27.9%), I^2^ = 0.97^b^	7.9% (6.5–9.5%)
Colorectal cancer^c^	11.7% (9.3–14.4%), I^2^ = 0.98^b^	9.0% (7.5–10.6%)	6.9% (5.4–8.5%)
Esophageal cancer	3.8% (1.1–7.8%), I^2^ = 0.77 ^b^	–	–
Gastric cancer	8.7% (7.6–9.9%), I^2^ = 0.2	10.2% (8.2–12.4%), I^2^ = 0.6 ^b^	5.6% (3.9–7.6%), I^2^ = 0
Liver cancer	0.0% (0.0–2.2%)	–	–
Pancreatic cancer	1.5% (0.6–2.7%), I^2^ = 0.15	–	0.0% (0.0–3.4%)
Rectal cancer^c^	–	8.7% (2.4–18.3%), I^2^ = 0.95^b^	–
Small bowel cancer	21.0% (15.8–26.7%), I^2^ = 0.77^b^	21.3% (13.5–30.2%)	–
**Genitourinary tract cancers**			
Bladder/urothelial cancer	4.4% (1.6–8.3%), I^2^ = 0.88^b^	–	–
Kidney cancer	0.8% (0.2–1.8%)	–	–
Penile cancer	0.0% (0.0–2.4%)	–	–
Prostate cancer	6.2% (0.7–16.2%), I^2^ = 0.97^b^	–	3.5% (1.3–6.6%)
Testicular cancer	–	0.0% (0.0–2.2%)	–
**Breast and gynaecological cancers**			
Breast cancer	1.3% (0.7–2.0%), I^2^ = 0.71^b^	–	–
Cervical cancer	1.9% (0.0–5.8%)	–	–
Endometrial cancer (EC)	26.8% (23.3–30.5%), I^2^ = 0.92^b^	27.6% (22.0–33.7%), I^2^ = 0.92^b^	–
Ovarian cancer	2.4% (0.5–5.5%), I^2^ = 0.92^b^	7.2% (5.1–9.6%)	–
Uterine cancer (excl. EC)	–	–	–
Vulvar cancer	–	–	–
**Thoracic cancers**			
Lung cancer	1.6% (0.5–3.4%), I^2^ = 0.76^b^	–	–
Thymic malignancy	3.9% (0.1–11.5%)	–	–
**Biliary tract cancers**			
Ampullary cancer	–	18.1% (11.9–25.3%)	–
Bile duct/gallbladder	3.8% (1.5–7.0%), I^2^ = 0.66	–	–
**Head and neck cancers, sarcomas, and skin cancers**			
Head and neck cancers	2.2% (0.1–6.1%), I^2^ = 0.86^b^	–	–
Sarcomas	0.5% (0.0–2.1%), I^2^ = 0.72	–	–
Skin cancers	4.2% (0.3–11.9%), I^2^ = 0.94^b^	–	9.1% (5.2–13.8%)
**Central nervous system tumours, endocrine tumours, neuroendocrine tumours, and other cancers**			
Brain tumours	3.8% (0.0–12.8%), I^2^ = 0.91^b^	–	5.1% (3.0–7.7%), I^2^ = 0.21
Endocrine tumours	0.7% (0.1–1.7%)	–	–
Neuroendocrine tumours	0.0% (0.0–1.2%)	–	–
Other cancers^d^	2.1% (0.1–6.0%), I^2^ = 0.82^b^	–	–

^a^Heterogeneity across studies was presented based on the point estimate of I^2^ score and heterogeneity test, if ≥ 2 records were available for each cancer type and stage group.

^b^Statistically significant heterogeneity across studies (p < 0.05).

^c^We analysed the pooled prevalence of pan-tumour biomarkers separately based on estimates including (1) colon cancer only, (2) rectal cancer only, and (3) tumours at any sites in the colon and the rectum, obtaining pooled prevalence estimates described as “colon cancer”, “rectal cancer”, and “colorectal cancer”, respectively.

^d^Other caners include cancer of unknown primary, cancer of unknown primary-neuro, germ cell tumour, peritoneal cancer, and underspecified cancer.

See Supplementary Tables S7 and S10 for the number of records included in the analysis and the references.

**Table 3:** Prevalence of microsatellite instability in adult solid tumours by cancer type and stage.

Cancer	Prevalence from random-effect model (95% CI)^a^		
	Overall	Early stage	Advanced stage
**Gastrointestinal cancers**			
Anal cancer	0.6% (0.1–1.4%), I^2^ = 0	–	–
Appendiceal cancer	2.1% (0.8–3.9%), I^2^ = 0.51	–	1.5% (0.0–4.5%)
Colon cancer^c^	13.0% (9.5–17.0%)	15.1% (7.6–24.7%), I^2^ = 1^b^	–
Colorectal cancer^c^	10.2% (6.6–14.5%), I^2^ = 1^b^	11.7% (8.1–16.0%), I^2^ = 0.96^b^	4.1% (2.5–6.0%), I^2^ = 0.9^b^
Esophageal cancer	2.4% (1.1–4.2%), I^2^ = 0.87^b^	–	–
Gastric cancer	8.5% (6.4–10.9%), I^2^ = 0.95^b^	10.6% (7.9–13.5%), I^2^ = 0.89^b^	8.2% (1.7–18.2%), I^2^ = 0.74^b^
Liver cancer	1.4% (0.4–2.9%), I^2^ = 0.23	–	0.5% (0.0–1.9%), I^2^ = 0.55
Pancreatic cancer	0.9% (0.4–1.5%), I^2^ = 0.9^b^	–	–
Rectal cancer^c^	–	9.2% (5.5–13.6%), I^2^ = 0.91^b^	–
Small bowel cancer	14.3% (5.4–26.3%), I^2^ = 0.95^b^	31.2% (13.4–52.4%), I^2^ = 0.9^b^	–
**Genitourinary tract cancers**			
Bladder/urothelial cancer	2.9% (0.7–6.5%), I^2^ = 0.94^b^	–	0.7% (0.3–1.1%)
Kidney cancer	0.4% (0.2–0.6%), I^2^ = 0	–	1.7% (0.0–5.2%)
Penile cancer	0.0% (0.0–1.8%)	–	–
Prostate cancer	2.3% (1.7–3.1%), I^2^ = 0.5	–	6.6% (3.5–10.5%)
Testicular cancer	–	–	0.9% (0.0–4.0%)
**Breast and gynaecological cancers**			
Breast cancer	0.6% (0.2–1.0%), I^2^ = 0.73^b^	–	0.2% (0.1–0.3%)
Cervical cancer	1.5% (0.7–2.6%), I^2^ = 0.66	–	–
Endometrial cancer (EC)	21.9% (15.1–29.6%), I^2^ = 0.98^b^	19.1% (15.6–22.8%)	17.6% (9.6–27.2%)
Ovarian cancer	1.7% (0.0–5.4%), I^2^ = 0.99^b^	–	–
Uterine cancer (excl. EC)	4.4% (3.2–5.7%)	–	–
Vulvar cancer	–	–	–
**Thoracic cancers**			
Lung cancer	0.4% (0.2–0.7%), I^2^ = 0.77^b^	–	0.0% (0.0–2.0%)
Thymic malignancy	3.8% (0.1–11.2%)	–	–
**Biliary tract cancers**			
Ampullary cancer	–	–	–
Bile duct/gallbladder	1.6% (1.0–2.3%), I^2^ = 0.69^b^	–	1.4% (0.0–4.2%)
**Head and neck cancers, sarcomas, and skin cancers**			
Head and neck cancers	0.5% (0.3–0.7%), I^2^ = 0	–	–
Sarcomas	1.8% (0.0–6.0%), I^2^ = 0.94^b^	1.4% (0.0–5.7%)	–
Skin cancers	1.3% (0.0–4.1%), I^2^ = 0.92^b^	–	–
**Central nervous system tumours, endocrine tumours, neuroendocrine tumours, and other cancers**			
Brain tumours	0.6% (0.2–1.2%)	0.5% (0.0–2.2%)	0.6% (0.2–1.3%)
Endocrine tumours	0.6% (0.0–2.2%), I^2^ = 0.53	–	–
Neuroendocrine tumours	1.4% (0.4–2.9%), I^2^ = 0.79^b^	–	–
Other cancers^d^	1.4% (0.8–2.2%), I^2^ = 0.85^b^	–	–

^a^Heterogeneity across studies was presented based on the point estimate of I^2^ score and heterogeneity test, if ≥ 2 records were available for each cancer type and stage group.

^b^Statistically significant heterogeneity across studies (p < 0.05).

^c^We analysed the pooled prevalence of pan-tumour biomarkers separately based on estimates including (1) colon cancer only, (2) rectal cancer only, and (3) tumours at any sites in the colon and the rectum, obtaining pooled prevalence estimates described as “colon cancer”, “rectal cancer”, and “colorectal cancer”, respectively.

^d^Other caners include cancer of unknown primary, cancer of unknown primary-neuro, germ cell tumour, peritoneal cancer, and underspecified cancer.

See Supplementary Tables S7 and S11 for the number of records included in the analysis and the references.

**Table 4:** Prevalence of high tumour mutational burden (≥ 10 mutations/Mb) in adult solid tumours by cancer type and stage.

Cancer	Prevalence from random-effect model (95% CI)^a^		
	Overall	Early stage	Advanced stage
**Gastrointestinal cancers**			
Anal cancer	–	–	15.7% (8.8–24.1%)
Appendiceal cancer	–	–	–
Colon cancer^c^	–	–	–
Colorectal cancer^c^	8.5% (7.1–10.1%)	–	–
Esophageal cancer	32.9% (26.9–39.2%)	–	–
Gastric cancer	13.9% (10.9–17.1%)	–	44.2% (17.3–73.0%), I^2^ = 0.9^b^
Liver cancer	1.4% (0.2–3.6%)	–	3.5% (0.0–11.2%), I^2^ = 0.93^b^
Pancreatic cancer	0.0% (0.0–1.7%)	–	–
Rectal cancer^c^	–	–	–
Small bowel cancer	19.1% (11.8–27.8%)	–	–
**Genitourinary tract cancers**			
Bladder/urothelial cancer	38.1% (21.4–56.4%), I^2^ = 0.98^b^	60.3% (53.3–67.1%)	43.6% (21.9–66.7%), I^2^ = 0.96^b^
Kidney cancer	–	–	–
Penile cancer	–	–	–
Prostate cancer	4.0% (2.3–6.3%), I^2^ = 0.91^b^	–	7.1% (3.9–11.2%)
Testicular cancer	–	–	3.7% (0.8–8.3%)
**Breast and gynaecological cancers**			
Breast cancer	7.2% (1.6–16.4%), I^2^ = 1^b^	–	9.4% (7.6–11.2%), I^2^ = 0.77^b^
Cervical cancer	23.7% (20.1–27.5%)	–	21.3% (12.7–31.4%)
Endometrial cancer (EC)	43.0% (39.1–46.9%)	–	18.3% (10.6–27.5%)
Ovarian cancer	–	–	1.6% (0.0–6.6%)
Uterine cancer (excl. EC)	–	–	–
Vulvar cancer	–	–	16.9% (9.0–26.6%)
**Thoracic cancers**			
Lung cancer	27.5% (16.0–40.8%), I^2^ = 0.99^b^	58.7% (52.4–65.0%)	29.0% (20.1–38.7%), I^2^ = 0.97^b^
Thymic malignancy	–	–	–
**Biliary tract cancers**			
Ampullary cancer	–	–	–
Bile duct/gallbladder	9.5% (3.3–18.2%)	–	0.0% (0.0–2.7%)
**Head and neck cancers, sarcomas, and skin cancers**			
Head and neck cancers	8.6% (1.8–19.4%), I^2^ = 0.95^b^	–	3.7% (0.5–9.1%)
Sarcomas	1.7% (0.4–3.6%), I^2^ = 0.14	–	–
Skin cancers	52.6% (49.7–55.5%)	–	42.5% (15.5–72.0%), I^2^ = 0.92^b^
**Central nervous system tumours, endocrine tumours, neuroendocrine tumours, and other cancers**			
Brain tumours	2.8% (0.0–8.3%)	–	–
Endocrine tumours	5.8% (3.8–8.2%)	–	2.5% (0.0–7.4%)
Neuroendocrine tumours	24.8% (22.9–26.7%)	–	5.7% (1.7–11.8%)
Other cancers^d^	–	–	–

^a^Heterogeneity across studies was presented based on the point estimate of I^2^ score and heterogeneity test, if ≥ 2 records were available for each cancer type and stage group.

^b^Statistically significant heterogeneity across studies (p < 0.05).

^c^We analysed the pooled prevalence of pan-tumour biomarkers separately based on estimates including (1) colon cancer only, (2) rectal cancer only, and (3) tumours at any sites in the colon and the rectum, obtaining pooled prevalence estimates described as “colon cancer”, “rectal cancer”, and “colorectal cancer”, respectively.

^d^Other caners include cancer of unknown primary, cancer of unknown primary-neuro, germ cell tumour, peritoneal cancer, and underspecified cancer.

See Supplementary Tables S7 and S12 for the number of records included in the analysis and the references.

#### Prevalence of MSI

The pooled overall MSI prevalence was similar to dMMR prevalence: high in endometrial (21.9%), small bowel (14.3%), colon (13.0%), colorectal (10.2%), and gastric cancers (8.5%), but generally low (~ 5%) for gynaecological cancers other than endometrial cancer and for other cancer types. Substantial variation was found in the pooled overall prevalence of MSI among gastrointestinal cancers (range: 0.6–14.3%). Similar to dMMR tumours, where stage-specific data were available, MSI tumours tended to be identified more often in early-stage disease as seen in colorectal and gastric cancers (Table 3). Substantial variation in the pooled overall prevalence of MSI was also found by cancer sub-type where data were available (e.g., 14.4% in endometroid/non-serous/mucinous carcinoma vs 1.7% in any ovarian carcinoma) (Supplementary Table S16). The pooled overall prevalence estimates of MSI in colorectal and gastric cancers based on PCR assays tended to be higher than based on gene panel sequencing, but with overlapping 95% CIs (Supplementary Table S21).

#### *Prevalence of high TMB (*≥ *10 mutations/Mb)*

The pooled overall prevalence of high TMB (≥ 10 mutations/Mb) was high in skin (52.6%), endometrial (43.0%), bladder/urothelial (38.1%), esophageal (32.9%) and lung (27.5%) cancers. Substantial variation was found in the pooled overall prevalence of TMB (≥ 10 mut/Mb) among gastrointestinal (range: 0.0–32.9%) and genitourinary tract cancers (range: 4.0–38.1%). By contrast to dMMR/MSI, advanced-stage tumours often showed substantially high prevalence of TMB, as e.g. seen in gastric and bladder/urothelial cancers (Table 4). Substantial variation in the prevalence of TMB (≥ 10 mutations/Mb) was also found by cancer sub-type where data were available (Supplementary Table S17; e.g., 58.2% in advanced non-melanoma *vs* 28.0% in advanced melanoma). For advanced lung cancer, the pooled overall prevalence of high TMB (≥ 10 mutations/Mb) based on gene panel sequencing tended to be higher than based on whole exome sequencing, but with very wide and overlapping 95% CIs (Supplementary Table S21). The patterns for prevalence of high TMB (≥ 20 mutations/Mb) were generally similar, see Supplementary Tables S14 and S18.

#### Comparison between the prevalence of dMMR/MSI/high TMB

In general, the results for MSI prevalence were similar to those for dMMR prevalence, with somewhat different prevalence profiles for high TMB. For some cancer types, the prevalence estimates were similar, e.g. for small bowel (21.0% overall prevalence of dMMR *vs* 19.1% of high TMB [≥ 10 mutations/Mb]), gastric cancers (8.7% vs 13.9%). However, differences in pooled prevalence estimates were more pronounced for other cancer types, e.g. endometrial (26.8% overall prevalence of dMMR vs 43.0% of high TMB [≥ 10 mutations/Mb]), with non-overlapping 95% CIs here and in the following examples, skin (4.2% vs 52.6%), bladder/urothelial (4.4% vs 38.1%), esophageal (3.8% vs 32.9%), lung (1.6% vs 27.5%) and cervical (1.9% vs 23.7%) cancers (Tables 2, 3, 4).

#### Publication bias

Of the four meta-analyses with ≥ 10 estimates, the funnel plots for the overall prevalence of dMMR in colorectal cancer, MSI in colorectal cancer, and MSI in gastric cancer were symmetric (Supplementary Fig. S2a). The plots showed horizontal scatter in line with the meta-analyses pooling results from large studies with varying effect sizes (noting the minimum sample size for inclusion in the review was 1000 for overall prevalence estimates for colorectal cancer, 400 for gastric cancer). This fits the choice of random-effect model and is in line with the significant heterogeneity of results indicated by a heterogeneity p-value of p < 0.05 in these analyses. For the analysis of MSI prevalence in early-stage gastric cancer, the plot was somewhat asymmetric, suggesting potential publication bias (Supplementary Fig. S2b); however, this may also be at least partially driven by two studies with substantially higher estimates than the other 9 studies (21.8–23.8% vs 7.3–11.4%). Notably, as opposed to the other nine studies, the two studies with higher estimates did not use PCR, which could contribute to differences in estimates. The funnel plot of the nine PCR-based prevalence estimates only was symmetric.

## Discussion

As the first structured review of the prevalence of all three pan-tumour biomarkers (dMMR, MSI, high TMB) in a pan-cancer setting, this study consolidated estimates for the prevalence of dMMR, MSI, and high TMB across cancers as well as by cancer type and stage for 32 different adult solid tumours across 13 tumour groups. In particular, with dMMR/MSI prevalence estimates for 28 cancer types, our study provides expanded and updated evidence compared to a previous review in 2018^13^, while also providing pooled estimates for high TMB at two common TMB thresholds.

The pooled overall pan-cancer prevalence of dMMR was comparable to that for MSI, and lower than that for high TMB (≥ 10 mut/Mb) (respective prevalence: 2.9% [95% CI 2.7–3.1%] vs 2.7% [95% CI 2.1–3.4%] vs 14.0% [95% CI 3.9–28.8%]). Our pan-cancer prevalence estimates for dMMR and MSI were substantially lower than the ~ 16% estimated in a previous review^12^, which is likely mainly explained by differences in primary study inclusions. In particular, the pan-cancer prevalence estimates in this review were based on estimates from pan-cancer studies only. By contrast, the previous review by Lorenzi et al. estimated pan-cancer prevalence by pooling estimates from all studies identified in a literature search, of which two thirds were related to colorectal, endometrial and stomach cancers, where dMMR and MSI are more common^12^. Our cancer-specific pooled overall prevalence estimates for dMMR and MSI were also generally similar, and for the cancer types included in a previous review, similar to the estimates reported in that study (see Appendix I.6 for detailed comparisons)^13^. Luchini et al. described the cancer-specific prevalence of dMMR/MSI in 14 different cancer types as well as the overlap between MSI and high TMB among 4186 patients, illustrating that dMMR, MSI and high TMB are related but do not always co-occur, with overlap depending on cancer type^13^.

By contrast, prevalence of high TMB in some cancer types was markedly different to dMMR/MSI, and it is helpful to consider three different categories: (1) high prevalence of dMMR/MSI and high TMB (dMMR^+^/MSI^+^/high TMB^+^); (2) low prevalence of dMMR/MSI, high prevalence of high TMB (dMMR^-^/MSI^-^/high TMB^+^); and (3) low to moderate prevalence of all three biomarkers (dMMR^-^/MSI^-^/high TMB^-^). As expected due to the mismatch repair defects intrinsic to Lynch syndrome (a cancer predisposition syndrome due to germline pathogenic variants in mismatch repair genes^27^), the dMMR^+^/MSI^+^/high TMB^+^ group includes some of the Lynch syndrome-associated cancers such as endometrial, colorectal, small bowel and gastric cancers. The dMMR^-^/MSI^-^/high TMB^+^ group includes head and neck, anal, cervical, esophageal, bladder/urothelial, lung and skin cancers. The dMMR^-^/MSI^-^/high TMB^-^ group includes both common and rare cancers (e.g., prostate, breast and bile duct/gall bladder cancers, sarcoma, brain tumour and endocrine tumour). These different categories likely reflect different contributions of carcinogenic mechanisms, which differ between cancer types. For example, some leading causes of dMMR^-^/MSI^-^/high TMB^+^ cancers include smoking, UV exposure and human papillomavirus infection^28^, which could contribute to high TMB without presence of dMMR.

Where stage-specific data were available, dMMR and MSI tumours tended to be identified more often in early-stage disease, whereas advanced-stage tumours often showed substantial prevalence of high TMB. This suggests that dMMR and MSI tumours may be more amenable to cure when diagnosed at an early stage, and thus do not advance to a late stage. This could be due to differences in immune environment preventing spread: tumours exhibiting dMMR or MSI have been reported to often have increased tumour-infiltrating lymphocytes and display gene signatures related to cytotoxic T lymphocytes, suggesting an enhanced antitumour immune environment limiting their ability to metastasise^29^. The prevalence of biomarkers could differ between tumours diagnosed at advanced stage and those that progressed to advanced stage after treatment; data on prior treatment of patients included in original studies would be required to examine such differences, which was generally not available for studies included in this review.

Our analysis also showed substantial increase in studies reporting high TMB (vs dMMR/MSI) in recent years in most tumour groups, but with very few studies of early-stage cancers. This potentially reflects recent advances in precision oncology with increased use of sequencing for clinical decision-making, which is still currently more focused on advanced-stage tumours.

We note that even where prevalence estimates of different biomarkers in a cancer type are similar, that does not necessarily imply that both are present in the same individuals’ tumours. To accurately estimate the total prevalence of any of the three biomarkers (as per drug approvals based on presence of any of dMMR/MSI/high TMB), the estimated concordance of all three based on measurements of the biomarkers in the same tumours would be needed. However, our review did not identify any large-scale original study addressing this aspect (see Appendix I.7 for a summary, with studies largely focused on gastrointestinal cancer or a TMB cut-off of ≥ 17 mutations/Mb). We therefore could not estimate the combined proportion of patients whose tumour exhibits at least one of the biomarkers, which is the target population of interest for some drug regulatory and reimbursement approvals. In particular, as the current approval for pembrolizumab is based on the presence of either dMMR or MSI^2^, the concordance of these markers in individual tumours is a crucial area for future research. In the future, large-scale studies reporting the concordance of all three biomarkers in individual tumours based on a high TMB cut-off of ≥ 10 mutations/Mb (or other cut-offs as per proposed drug indications) would help address this evidence gap.

Prevalence estimates for the three pan-tumour biomarkers such as provided in this study (alongside future accurate estimates for concordance of the biomarkers on the level of individual tumours) can also help address key questions for the successful implementation of biomarker testing in practice: who should be tested for which of the three pan-tumour biomarkers, when, and using which test? The European Society for Medical Oncology recommended immunohistochemistry tests for loss of expression of four MMR proteins in Lynch syndrome-related cancer types (colorectal, endometrial, small intestine, urothelial, gliomas/glioblastomas, and sebaceous gland), with PCR-based MSI test on five microsatellite markers if immunohistochemistry results are equivocal^13^. For rare cancers and cancers not belonging to the spectrum of Lynch syndrome and without existing access to immune checkpoint inhibitors, sequencing-based assays coupling MSI and TMB analysis could become the method of choice for decisions on targeted treatment^13^. These tests need to be performed before or during the standard treatment of advanced (unresectable or metastatic) solid tumours, or ideally at the time of diagnosis to ensure test results are available when treatment decisions need to be made (though this could increase the number of tested patients and thus the healthcare costs)^17^. However, there still are outstanding questions regarding different assays as eligible companion tests for targeted treatments. MMR immunohistochemistry is well-established and validated in colorectal and endometrial cancers, with limited data on its reliability for other cancer types^30,31^. While increasingly more common, genomic sequencing is currently not universally available in a clinical setting even in high-income countries (with resources limited further in low- and middle-income countries), and TMB assessment is currently not standardised and lacking consensus on the most-appropriate threshold^32^. In the future, in-depth consideration of pan-tumour biomarker prevalence by histologic subtype could also help identify the most efficient biomarker testing approaches.

To help inform assessments of potential testing pathways, the prevalence estimates obtained from our review will later be used to estimate the number of cancer patients with each of the three pan-tumour biomarkers likely to require any curative treatment over the next 5 years. However, the uncertainty in the biomarker or combination of biomarkers required for access to future treatments, assay development and/or accessibility, as well as uncertainty in prevalence estimates (e.g., wide 95% CIs) will result in uncertainty for the population projections and ultimately health system planning. Notably, common cancers with low biomarker prevalence and less common cancers with high biomarker prevalence could contribute similar numbers of patients who might be eligible for the targeted treatment (see Supplementary Table S22 for an illustrative example considering dMMR estimates for lung cancer and for gastric cancer in the Australian population). Thus, both common and less common cancers are important to consider for health system planning related to dMMR/MSI/high TMB.

Both our scoping review and the currently available evidence have some limitations. Limitations of the available evidence include lack of information on the prevalence of the pan-tumour biomarkers (1) for some cancer types and stages; (2) by previous cancer treatment; and (3) by patient characteristics (e.g. age, BMI). Another key limitation is the lack of population-based estimates with patients sampled from comprehensive cancer registries that are not subject to any selection bias. This aspect also affects the pooled estimates obtained in this review, which will be influenced by biases present in the original studies (noting studies generally did not adjust for ascertainment bias), and thus may not reflect the true prevalence among all patients. For example, overall prevalence estimates in many clinical study cohorts (e.g. the Foundation Medicine Database) are likely to be predominantly based on advanced-stage tumours, for which inclusion in clinical trials and research studies is more common. This might have resulted in lower overall pan-cancer prevalence of dMMR/MSI, given the relatively lower prevalence of dMMR/MSI in advanced-stage cancers. Moreover, data from clinical trials would also be influenced by the trial eligibility criteria. Lastly, for most cancer types, we could not investigate the potential heterogeneity of estimates by assays used due to insufficient number of studies for each cancer type, stage group and assay. For example, our prevalence estimate of MSI for ovarian cancer is lower than that reported in the recent systematic review^33^, however this difference could be potentially explained by differences in study populations (with our review including two recent large studies that were not included in the published review), and/or by differences in the assays with higher MSI prevalence estimates based on PCR assays and lower prevalence estimates based on gene panel sequencing.

As a risk of bias assessment of the 201 studies included in meta-analyses was not part of this scoping review, we could not examine the impact of non-representativeness or other aspects of bias on the prevalence estimates from original studies. In the future, integration of clinical data on biomarker status into routinely collected, population-wide administrative datasets and cancer registries would allow to efficiently examine both current testing approaches and biomarker prevalence. Meanwhile, hospital-based studies may still provide acceptable estimates for patient populations that might be considered for targeted therapies in the near term, thus helping inform health system planning and budget impact estimates. Another limitation of our review is that some studies might have been missed during the initial screen and full-text review performed by one person. However, our process identified all relevant studies that were also included in previous cancer-specific systematic reviews/meta-analyses. While our meta-analyses did not include all studies of pan-tumour biomarkers, with small studies excluded for cancer types for which many larger studies were available, these small studies would unlikely have much impact on the pooled estimates. We performed random-effect meta-analyses using the Freeman-Tukey transformation. The random-effect model allows the true effect sizes to differ across the studies, as opposed to the assumption of one true common effect size across all studies in a fixed-effect model^34^. As different studies included in our review were based on different patient populations, the random-effect model was deemed more appropriate. The Freeman-Tukey double arcsine is a popular choice in systematic reviews of prevalence as it stabilised variances^35^. While problems have been reported for meta-analyses of single proportions with highly skewed sample sizes^36^, this is not the case in the current analyses. Despite the limitations, this is one of the most comprehensive analyses of the prevalence of the three key pan-tumour biomarkers in a fast-moving field with a substantial and rapidly growing number of publications.

## Conclusions

This review reports the estimated prevalence of dMMR, MSI, high TMB across cancers as well as for specific cancer types and stages, providing timely evidence to inform health technology assessments for drug approvals based on these pan-tumour biomarkers to support appropriate evaluation of precision oncology approaches. Rates for both common cancers with low prevalence of dMMR/MSI/high TMB and rare cancers with high prevalence of dMMR/MSI/high TMB are important for projections of future patient populations to enable health system planning.

## Supplementary Information

Supplementary Information 1.

Supplementary Information 2.

Supplementary Information 3.

## Data Availability

Data supporting the findings of this study are available within the article and the supplementary information.

## Abbreviations

dMMR: Mismatch repair deficiency
MSI: Microsatellite instability
TMB: Tumour mutational burden
