# Supplementary material for: A scoping review and meta-analysis on the prevalence of pan-tumour biomarkers (dMMR, MSI, high TMB) in different solid tumours
Source: Sci Rep. 2022 Nov 28;12:20495. doi: 10.1038/s41598-022-23319-1 (PMC9705554; doi:10.1038/s41598-022-23319-1)
Supplement: Supplementary file 1 — Supplementary Information 1. [file 41598_2022_23319_MOESM1_ESM.pdf]

# A scoping review and meta-analysis on the prevalence of pan-tumour biomarkers (dMMR, MSI, high TMB) in different solid tumours

## *Appendix I. Detailed methods and results*

**RUNNING TITLE: Prevalence of dMMR, MSI and high TMB in different solid tumours**

**CORRESPONDENCE:** Dr Julia Steinberg

The Daffodil Centre

Tel: + 61293080283; Fax: + 61 2 8302 3550

Email: julia.steinberg@nswcc.org.au

## Table of contents

List of supplementary tables ........ 3

List of supplementary figures ........ 5

1. Scoping review protocol and PRISMA-ScR Checklist ........ 6

   1) Scoping review protocol ........ 6

   2) Preferred Reporting Items for Systematic reviews and Meta-Analyses extension for Scoping Reviews (PRISMA-ScR) Checklist ........ 9

2. Background, search strategy and section criteria ........ 11

3. Data extraction ........ 13

   1) Tumour group categories ........ 13

   2) Cancer stage categories ........ 15

4. Overview of literature reporting the prevalence of dMMR/MSI/high TMB ........ 16

5. Detailed results ........ 17

   1) Number of prevalence estimates included in each cancer-specific meta-analysis ........ 17

   2) Prevalence of high TMB (≥20 mutations/Mb) ........ 23

   3) Sub-group analyses of the prevalence of dMMR/MSI/high TMB ........ 26

   4) Sensitivity analyses of the prevalence of dMMR/MSI/high TMB ........ 31

   5) Publication bias ........ 32

   6) Illustrative example: estimated numbers of gastric and lung cancer patients with dMMR and, separately, high TMB tumours for two cancer types in Australia in 2021 ........ 33

6. Comparison with prior reviews reporting the prevalence of dMMR/MSI/high TMB ........ 34

7. Studies reporting the overlap between high TMB and dMMR/MSI status in individual tumours 36

8. References ........ 39

## List of supplementary tables


Supplementary Table S1. PRISMA-ScR Checklist ........ 9

Supplementary Table S2. Final search terms and the number of titles and abstracts identified in EMBASE and Medline searches on 01/02/2021 ........ 11

Supplementary Table S3. Detailed inclusion and exclusion criteria and information sources ........ 11

Supplementary Table S4. Adult solid tumour group categories ........ 13

Supplementary Table S5. Cancer stage categories ........ 15

Supplementary Table S6. Minimum sample size thresholds for inclusion of studies in meta-analyses ........ 15

Supplementary Table S7. Number of prevalence estimates included in each cancer-specific meta-analysis ........ 17

Supplementary Table S8. Numbers of prevalence estimates included in stage-specific meta-analyses ........ 18

Supplementary Table S9. Numbers of prevalence estimates included in cancer-specific meta-analyses by pan-tumour biomarker and assays used ........ 18

Supplementary Table S10. References of studies included in each cancer-specific meta-analysis of dMMR prevalence ........ 19

Supplementary Table S11. References of studies included in each cancer-specific meta-analysis of MSI prevalence ........ 20

Supplementary Table S12. References of studies included in each cancer-specific meta-analysis of high TMB (≥10 mutations/Mb) prevalence ........ 21

Supplementary Table S13. References of studies included in each cancer-specific meta-analysis of high TMB (≥20 mutations/Mb) prevalence ........ 22

Supplementary Table S14. Prevalence of high tumour mutational burden (≥20 mutations/Mb) in adult solid tumours by cancer type and stage group ........ 24

Supplementary Table S15. Prevalence of dMMR in adult solid tumours by cancer sub-type and stage group ........ 26

Supplementary Table S16. Prevalence of MSI in adult solid tumours by cancer sub-type and stage group ........ 27

Supplementary Table S17. Prevalence of high TMB (≥10 mutations/Mb) in adult solid tumours by cancer sub-type and stage group ........ 28

Supplementary Table S18. Prevalence of high TMB (≥20 mutations/Mb) in adult solid tumours by cancer sub-type and stage group ........ 29

Supplementary Table S19. Prevalence of dMMR/MSI/high TMB by tumour group, based on studies reporting prevalence estimates on tumour group level only ........ 30

Supplementary Table S20. Pooled prevalence of dMMR/MSI in colorectal, colon and rectal cancers 30

Supplementary Table S21. Prevalence of MSI/high TMB by assays used 31

Supplementary Table S22. Estimated numbers of gastric and lung cancer patients with dMMR and, separately, with high TMB (≥10 mutations/Mb) tumours in Australia in 2021 33

Supplementary Table S23. Comparison of the prevalence of dMMR/MSI between a prior systematic review and the current analysis 35

Supplementary Table S24. Studies reporting the concordance between high TMB and dMMR/MSI status in individual tumours 36

Supplementary Table S25. Individual-level overlap between high TMB (≥17 mutations/Mb) and MSI tumour status as reported in Vanderwalde et al. (Cancer Medicine 2018) 38

## List of supplementary figures

Supplementary Fig. S1. Relative proportion of studies reporting the prevalence of high TMB *vs* dMMR/MSI within each tumour group ........................................................................................ 16

Supplementary Fig. S2. Funnel plots for meta-analyses pooling 10+ estimates, showing the transformed proportions and their standard errors. a) Overall prevalence of dMMR and MSI in colorectal cancer and MSI in gastric cancer; b) Prevalence of MSI in early-stage gastric cancer ........ 32

## 1. Scoping review protocol and PRISMA-ScR Checklist

### 1) Scoping review protocol

| | |
|---|---|
| Title of the review | A scoping review and meta-analysis on the prevalence of pan-tumour biomarkers (dMMR, MSI, high TMB) in different solid tumours |
| First reviewer | Dr Yoon-Jung Kang |
| Team of reviewers | Dr Julia Steinberg, Ms Sophie O'Haire, Dr Fanny Franchini |
| Supervisor/Project PI | Dr Julia Steinberg |

**1. Background to review**

With increasing application of genomic medicine, many research efforts have been focusing on the identification of so-called "pan-tumour biomarkers", which can predict favourable response to a treatment for cancers originating from any tumour site.[1] A few drugs have recently received tumour-agnostic approval based on presence of a pan-tumour biomarker, including immune checkpoint inhibitors (e.g. pembrolizumab [Merck & Co., Inc.]), for the treatment of patients with solid tumours exhibiting mismatch repair deficiency (dMMR), microsatellite instability (MSI), or high tumour mutational burden (TMB).[2]

Given the relatively high cost of pembrolizumab and other potential targeted treatments,[3] a key question to inform health system planning and budget impact evaluations is how many patients might be eligible for these treatments based on the presence of these biomarkers. In particular, budget impact evaluations are an integral aspect of health technology assessments that summarise the information needed to inform policy and funding decisions (including e.g. drug efficacy, effectiveness, cost-effectiveness, and re-imbursement costs).[4] To facilitate such assessments, it is therefore crucial to map and consolidate the recent available evidence on the prevalence of the pan-tumour biomarkers, where possible, by cancer type as well as across all cancers. As approvals based on dMMR/MSI/high TMB currently focus on patients with advanced-stage disease, and biomarker prevalence may vary between cancer stages,[5] stage-specific prevalence estimates are also important where data are available.

**2. Aims and specific objectives**

Aim

To identify the available evidence on the prevalence of each of dMMR, MSI and high TMB in adult and paediatric solid tumours, by cancer type and cancer stage. Specific objectives are to:

1) provide a broader overview of studies reporting the prevalence of these three pan-tumour biomarkers; and
2) consolidate the evidence by cancer type and cancer stage. To the best of our knowledge, this is the first structured review on the prevalence of all three pan-tumour biomarkers (dMMR, MSI, high TMB) in a pan-cancer setting.

**3. a) Criteria for including studies in the review**

| | |
|---|---|
| **Population** | Adult and paediatric cancer patients with solid tumours |
| **Concept** | The prevalence of the following biomarkers by cancer type and cancer stage (if available):<br>• mismatch repair deficiency (dMMR)<br>• microsatellite instability (MSI)<br>• high tumour mutational burden (TMB) |
| **Context** | No limit to any setting |
| **Type of evidence sources and publication status** | • Primary research studies (including case series and analyses of large-scale genomic data)<br>• review articles (including scoping reviews, systematic reviews and meta-analyses), letters, opinions and editorials that contain extractable data |
| **Publication date** | 01/01/2018-31/01/2021 |
| **Language** | English only |

Note: See Supplementary Table S3 for the Detailed inclusion and exclusion criteria and information sources.

3. b) Criteria for excluding studies not covered in inclusion criteria

| | |
|---|---|
| **Population** | Highly selected population (e.g. based on restriction to patients with family history or inherited predisposition to cancer, specific rare histological cancer subtypes only) |
| **Concept** | • Sample size ≤ 50<br>• TMB detected from circulating tumour DNA[6] |
| **Context** | None |
| **Type of evidence sources and publication status** | • Case reports<br>• Conference abstracts<br>• In vitro studies and in vivo studies<br>• Non-academic literature (e.g., Health Technology Assessment reports) |

Note: See Supplementary Table S3 for the Detailed inclusion and exclusion criteria and information sources.

**4. Search methods**

| | |
|---|---|
| **Electronic databases** | MEDLINE, EMBASE |
| **Other methods used for identifying relevant research** | None |
| **Journals hand searched** | None |

**5. Methods of review**

| | |
|---|---|
| **Details of methods** | Title and abstract screening will be performed by one reviewer, with two reviewers double-screening 25% of articles to ensure reliability.<br>Full-text review will be performed by one reviewer, with a second reviewer independently assessing 10% of studies to ensure reliability. |
| **Quality assessment** | Not applicable |
| **Data extraction** | Data extraction will be performed in duplicate for 10% of studies. If concordance is high, data extraction will be completed by one reviewer for the remaining studies. |
| **Narrative synthesis** | Not applicable |

| | |
|---|---|
| **Meta-analysis** | We will perform random-effect meta-analyses, using the inverse variance heterogeneity model to pool the Freeman-Tukey transformed proportions of cases with dMMR, MSI, or high TMB.[7] Heterogeneity across studies (for meta-analyses with ≥2 estimates) will be assessed based on the $I^2$ score estimate, with higher $I^2$ score indicating higher level of heterogeneity, and based on the heterogeneity test p-value (defining significance at $p<0.05$). All statistical analyses will be performed using R (Version 4.1.1) and the package "meta" (version 4.19-1). |
| **Grading evidence** | Not applicable |

### 6. Presentation of results

We will present a narrative broader overview of studies reporting the prevalence of the three pan-tumour biomarkers, with figures displaying the proportion of studies with specific characteristics (e.g. cancer type or assay used) by publication year.

Meta-analysis results will be presented using tables and forest plots as appropriate.

## 2) Preferred Reporting Items for Systematic reviews and Meta-Analyses extension for Scoping Reviews (PRISMA-ScR) Checklist

**Supplementary Table S1. PRISMA-ScR Checklist**

| SECTION | ITEM | PRISMA-ScR CHECKLIST ITEM | REPORTED ON SECTION |
|---|---|---|---|
| ***TITLE*** | | | |
| **Title** | 1 | Identify the report as a scoping review. | Title |
| ***ABSTRACT*** | | | |
| **Structured summary** | 2 | Provide a structured summary that includes (as applicable): background, objectives, eligibility criteria, sources of evidence, charting methods, results, and conclusions that relate to the review questions and objectives. | Abstract |
| ***INTRODUCTION*** | | | |
| **Rationale** | 3 | Describe the rationale for the review in the context of what is already known. Explain why the review questions/objectives lend themselves to a scoping review approach. | Introduction paragraph 3 |
| **Objectives** | 4 | Provide an explicit statement of the questions and objectives being addressed with reference to their key elements (e.g., population or participants, concepts, and context) or other relevant key elements used to conceptualize the review questions and/or objectives. | Introduction paragraph 3 |
| ***METHODS*** | | | |
| **Protocol and registration** | 5 | Indicate whether a review protocol exists; state if and where it can be accessed (e.g., a Web address); and if available, provide registration information, including the registration number. | Methods paragraph 2 |
| **Eligibility criteria** | 6 | Specify characteristics of the sources of evidence used as eligibility criteria (e.g., years considered, language, and publication status), and provide a rationale. | Methods (“Search strategy” sub-section) |
| **Information sources*** | 7 | Describe all information sources in the search (e.g., databases with dates of coverage and contact with authors to identify additional sources), as well as the date the most recent search was executed. | Methods (“Search strategy” sub-section) |
| **Search** | 8 | Present the full electronic search strategy for at least 1 database, including any limits used, such that it could be repeated. | Methods (“Search strategy” sub-section); Appendix I.2 |
| **Selection of sources of evidence†** | 9 | State the process for selecting sources of evidence (i.e., screening and eligibility) included in the scoping review. | Methods (“Selection criteria” sub-section); Figure 1; Appendix I.2 |
| **Data charting process‡** | 10 | Describe the methods of charting data from the included sources of evidence (e.g., calibrated forms or forms that have been tested by the team before their use, and whether data charting was done independently or in duplicate) and any processes for obtaining and confirming data from investigators. | Methods (“Data extraction” sub-section) |
| **Data items** | 11 | List and define all variables for which data were sought and any assumptions and simplifications made. | Methods (“Data extraction” sub-section) |
| **Critical appraisal of individual sources of evidence§** | 12 | If done, provide a rationale for conducting a critical appraisal of included sources of evidence; describe the methods used and how this information was used in any data synthesis (if appropriate). | Methods (“Quality assessment and risk of bias” sub-section) |

| SECTION | ITEM | PRISMA-ScR CHECKLIST ITEM | REPORTED ON SECTION |
|---|---|---|---|
| **Synthesis of results** | 13 | Describe the methods of handling and summarizing the data that were charted. | Methods ("Synthesis of results" sub-section) |
| ***RESULTS*** | | | |
| **Selection of sources of evidence** | 14 | Give numbers of sources of evidence screened, assessed for eligibility, and included in the review, with reasons for exclusions at each stage, ideally using a flow diagram. | Results ("Search results" sub-section) |
| **Characteristics of sources of evidence** | 15 | For each source of evidence, present characteristics for which data were charted and provide the citations. | Results ("Search results" sub-section) |
| **Critical appraisal within sources of evidence** | 16 | If done, present data on critical appraisal of included sources of evidence (see item 12). | Not performed as per Item 12 above |
| **Results of individual sources of evidence** | 17 | For each included source of evidence, present the relevant data that were charted that relate to the review questions and objectives. | Results ("Overview of literature reporting the prevalence of dMMR/MSI/high TMB" sub-section); Results ("Meta-analyses of the prevalence of dMMR/MSI/high TMB" sub-section) |
| **Synthesis of results** | 18 | Summarize and/or present the charting results as they relate to the review questions and objectives. | Results; Tables 1-4; Appendix I.4; Appendix. I.5 |
| ***DISCUSSION*** | | | |
| **Summary of evidence** | 19 | Summarize the main results (including an overview of concepts, themes, and types of evidence available), link to the review questions and objectives, and consider the relevance to key groups. | Discussion paragraphs 1-5 |
| **Limitations** | 20 | Discuss the limitations of the scoping review process. | Discussion paragraphs 9-10 |
| **Conclusions** | 21 | Provide a general interpretation of the results with respect to the review questions and objectives, as well as potential implications and/or next steps. | Conclusions |
| ***FUNDING*** | | | |
| **Funding** | 22 | Describe sources of funding for the included sources of evidence, as well as sources of funding for the scoping review. Describe the role of the funders of the scoping review. | Funding |

JBI = Joanna Briggs Institute; PRISMA-ScR = Preferred Reporting Items for Systematic reviews and Meta-Analyses extension for Scoping Reviews.

* Where sources of evidence (see second footnote) are compiled from, such as bibliographic databases, social media platforms, and Web sites.

† A more inclusive/heterogeneous term used to account for the different types of evidence or data sources (e.g., quantitative and/or qualitative research, expert opinion, and policy documents) that may be eligible in a scoping review as opposed to only studies. This is not to be confused with information sources (see first footnote).

‡ The frameworks by Arksey and O'Malley (6) and Levac and colleagues (7) and the JBI guidance (4, 5) refer to the process of data extraction in a scoping review as data charting.

§ The process of systematically examining research evidence to assess its validity, results, and relevance before using it to inform a decision. This term is used for items 12 and 19 instead of "risk of bias" (which is more applicable to systematic reviews of interventions) to include and acknowledge the various sources of evidence that may be used in a scoping review (e.g., quantitative and/or qualitative research, expert opinion, and policy document).

*From: Tricco AC, Lillie E, Zarin W, O'Brien KK, Colquhoun H, Levac D, et al. PRISMA Extension for Scoping Reviews (PRISMAScR): Checklist and Explanation. Ann Intern Med. 2018;169:467–473. doi: 10.7326/M18-0850.*[8]

## 2. Background, search strategy and section criteria

dMMR indicates a reduced ability to repair DNA damage, typically due to loss of function in the MMR genes *MLH1*, *MSH2*, *MSH6*, and/or *PMS2*.[9] A contributing factor (especially for some cancer types) can be germline pathogenic variants in the MMR genes ('Lynch syndrome', with these variants increasing cancer predisposition).[10] dMMR tumours are very often hypermutated and thus exhibit MSI and/or high TMB. Similarly, MSI can lead to high TMB and vice versa, but both MSI and high TMB can also arise through non-dMMR mechanisms.[9] The hypermutation can lead to the generation of a higher number of neoantigens, which is related to better immune checkpoint inhibitor response, thus linking these biomarkers to the targeted treatments.[11]

**Supplementary Table S2. Final search terms and the number of titles and abstracts identified in EMBASE and Medline searches on 01/02/2021**

| No | Search terms | Results |
|---|---|---|
| 1 | (microsatellite instability or msi).tw. | 28,944 |
| 2 | (microsatellite adj2 unstable).tw. | 1,232 |
| 3 | (mismatch repair or mmr or dmmr or mmrd).tw. | 39,125 |
| 4 | (tmb or tumo?r mutation burden* or tumo?r mutation load* or tml).tw. | 11,006 |
| 5 | 1 or 2 or 3 or 4<br>***Articles with explicit keywords related to MMR, MSI or TMB*** | 69,871 |
| 6 | (cancer* or carcinoma* or tumo?r* or neoplas* or malignan*).tw. | 8,189,812 |
| 7 | 5 and 6 | 40,630 |
| 8 | limit 7 to (English language and humans) | 33,059 |
| **9** | **Remove case reports, conference abstracts, duplicates (EMBASE and Medline) and limit to studies published between 01/01/2018 and 31/01/2021** | **3,890** |

**Supplementary Table S3. Detailed inclusion and exclusion criteria and information sources**

| Category | Details |
|---|---|
| Population | Inclusion criteria: adult and paediatric cancer patients with solid tumours (see Supplementary Table S4)<br>• Data for children were considered separately where available<br>• Data from populations of any ancestry were included, as early evidence suggests no substantial differences in the prevalence of dMMR and MSI among colorectal cancers from different ancestries[12, 13]<br><br>Exclusion criteria: highly selected population, e.g. based on restriction to<br>• patients with family history or inherited predisposition to cancer (e.g., Lynch syndrome probands or their families)<br>• patients with specific medical conditions such as immunocompromise (e.g., transplant patients, HIV patients)<br>• patients exposed to a specific risk factor (e.g., individuals with gastric cancers with a prior Helicobacter Pylori infection)<br>• specific rare histological cancer subtypes only (e.g., sebaceous skin neoplasm, neuroendocrine carcinoma of the cervix) |

| Category | Details |
|---|---|
| | • specific molecular cancer subtype only (e.g., colorectal cancer with *BRAF* mutation, triple-negative breast cancer), except for ovarian cancer where guidelines generally recommend dMMR/MSI testing in specific histologic subtypes only [e.g., endometrioid, clear cell carcinoma])[14] |
| Concept | <u>Main review question</u><br>The prevalence of the following biomarkers by cancer type and cancer stage (if available):<br>• mismatch repair deficiency (dMMR; determined by immunohistochemistry as loss of MLH1/MSH2/MSH6/PMS2 staining, or based on genetic loss of function of *MLH1/MSH2/MSH6/PMS2* identified from gene panel test, or by whole genome or whole exome sequencing)<br>• microsatellite instability (MSI; instability of 2+ microsatellite markers determined by PCR, including automated MSI tests [e.g., MSISensor], or based on a study-specific definition of MSI based on a gene panel test, whole genome or whole exome sequencing)<br>• high tumour mutational burden (TMB, [e.g., ≥10 mutations per Mb or ≥20 mutations per Mb] based on a gene panel test, whole genome or whole exome sequencing)<br><br><u>Additional pre-planned review questions</u><br>• The prevalence of dMMR, MSI and high TMB by cancer type and cancer stage (if available):<br>○ in adult and paediatric patients at cancer diagnosis prior to cancer treatment;<br>○ in adult and paediatric patients with advanced cancer whose tumour has progressed following prior systemic treatment;<br>○ in adult and paediatric patients based on different assays.<br><br>Exclusion criteria:<br>• Sample size ≤ 50<br>• TMB detected from circulating tumour DNA[6] |
| Context | No limit to any setting |
| Type of evidence sources and publication status | Inclusion criteria: primary research studies (including case series and analyses of large-scale genomic data [e.g., The Cancer Genome Atlas]), review articles (including scoping reviews, systematic reviews and meta-analyses), letters, opinions and editorials that contain extractable data.<br><br>Exclusion criteria:<br>• Case reports<br>• Conference abstracts<br>• In vitro studies and in vivo studies<br>• Non-academic literature (e.g., Health Technology Assessment reports) |
| Publication date | 01/01/2018-31/01/2021 |
| Language | English only |
| Database | MEDLINE and EMBASE |
| Date of executing final search | 01/02/2021 |

dMMR – mismatch repair deficiency; MSI – microsatellite instability; TMB – tumour mutational burden; PCR = polymerase chain reaction; HIV = human immunodeficiency virus.

## 3. Data extraction

### 1) Tumour group categories

Tumour groups were categorised based on the anatomical site where possible, with 13 tumour group categories in total. Haematological tumours and lymphoma (including non-Hodgkin's lymphoma) were excluded from the scoping review. The final classification was based on each included study, as not all studies provided the specific International Classification of Diseases (ICD) codes for included cancer types, and classification of some cancers has changed over time (e.g. gastroesophageal junction cancer was classified as esophageal cancer in the 7th editing of the UICC/AJCC TNM classification, but as gastric cancer in the 8th edition[15]).

**Supplementary Table S4. Adult solid tumour group categories**

| Tumour group | Cancer types and selected caner sub-types included in meta-analyses |
|---|---|
| **Central nervous system tumours** | • Brain tumours: low-grade glioma, high-grade glioma, glioblastoma (stage IV glioma) |
| **Skin cancers** | • Skin cancers, with the following sub-types in sub-group analyses<br>○ Melanoma<br>○ Non-melanoma (including cutaneous squamous cell carcinoma) |
| **Sarcomas** | • Sarcomas, with the following sub-types in sub-group analyses<br>○ Bone sarcoma<br>○ Soft tissue sarcoma (including liposarcoma, retroperitoneal sarcoma, nerve sheath tumour, uterine sarcoma, gastrointestinal stromal tumour) |
| **Endocrine tumours[a]** | • Endocrine tumours, with the following sub-types in sub-group analyses<br>○ Adrenocortical cancer<br>○ Thyroid carcinoma |
| **Neuroendocrine tumours[b]** | • Neuroendocrine tumours, with the following sub-types in sub-group analyses<br>○ Carcinoid<br>○ Gastrointestinal neuroendocrine tumours |
| **Head and neck cancers[c]** | • Head and neck cancers, with the following sub-types in sub-group analyses<br>○ Nasopharyngeal carcinoma<br>○ Oral cavity carcinoma<br>○ Salivary gland carcinoma |
| **Thoracic cancers** | • Lung cancer, with the following sub-types in sub-group analyses<br>○ Mesothelioma<br>○ Small cell lung cancer<br>○ Non-cell lung cancer<br>• Thymic malignancy |
| **Biliary tract cancers** | • Ampullary cancer<br>• Bile duct/gallbladder, with the following sub-types in sub-group analyses<br>○ Bile duct cancer (intrahepatic/extrahepatic cholangiocarcinoma only): Although intrahepatic cholangiocarcinoma is sometimes classified as a type of liver cancer since it occurs in the parts of the bile ducts within the liver, it is defined as bile duct cancer in our analysis.<br>○ Gallbladder cancer |
| **Gastrointestinal cancers** | • Anal cancer<br>• Appendiceal cancer |

| Tumour group | Cancer types and selected caner sub-types included in meta-analyses |
|---|---|
| | • Colon cancer only<br>• Colorectal cancer (any sites in the colon and the rectum)<br>• Esophageal cancer<br>• Gastric cancer (including gastroesophageal junction cancer): Gastroesophageal junction cancer was classified as esophageal cancer in the 7th editing of the UICC/AJCC TNM classification, but as gastric cancer in the 8th edition.[15] Studies of biomarker prevalence in esophageal cancer or gastric cancer also do not always specify whether gastroesophageal junction cancers are included or excluded.<br>• Liver cancer (including hepatocellular carcinoma)<br>• Pancreatic cancer<br>• Rectal cancer only<br>• Small bower cancer |
| **Genitourinary tract cancers** | • Bladder/urothelial cancer (including both urothelial carcinoma and upper urinary tract urothelial carcinoma since studies often reported the prevalence in urothelial carcinoma)<br>• Kidney cancer (including renal cell carcinoma)<br>• Penile cancer<br>• Prostate cancer<br>• Testicular cancer |
| **Breast cancer** | • Breast cancer |
| **Gynaecological cancers** | • Cervical cancer<br>• Endometrial cancer (including endometrioid carcinoma)<br>• Ovarian cancer, with the following sub-types in sub-group analyses only<br>o dMMR/MSI enriched histological subtypes (endometroid carcinoma, non-serous/non-mucinous carcinoma, clear cell carcinoma): Guidelines generally recommends tumour testing for dMMR/MSI in these histologic types only.[14] Therefore, studies focused on these histologic sub-types were included in the cancer sub-group analyses only.<br>• Uterine cancer (excluding endometrial cancer): if reported prevalence separately for endometrial cancer and uterine cancer<br>• Vulvar cancer |
| **Others** | • Peritoneal cancer<br>• Germ cell tumours<br>• Cancer of unknown primary<br>• Cancer of unknown primary-neuro<br>• Underspecified |

dMMR = mismatch repair deficiency; MSI = microsatellite instability.

[a] Of the seven studies reporting the prevalence of dMMR/MSI/high tumour mutational burden (TMB) in endocrine tumours; i) three studies reported the prevalence at this level only, with one study in adrenocortical cancer and three studies in thyroid cancer. Therefore, cancer-specific analysis was performed at this level.

[b] Of the seven studies reporting the prevalence of dMMR/MSI/high TMB in neuroendocrine tumours, three studies reported the prevalence at this level only, with one study in carcinoid and three studies in gastrointestinal neuroendocrine tumours. Therefore, cancer-specific analysis was performed at this level.

[c] Of the 12 studies reporting the prevalence of dMMR/MSI/high TMB in head and neck cancers, five studies reported the prevalence at this level only, with one study each in nasopharyngeal carcinoma and oral cavity carcinoma and two studies in salivary gland carcinoma. Therefore, cancer-specific analysis was performed at this level.

## 2) Cancer stage categories

Not all studies provided details on how different cancer stages were defined, and the categorisation was not always consistent between studies. As current drug approvals based on dMMR/MSI/high TMB largely focus on unresectable or metastatic solid tumours, we separately considered data on biomarker prevalence for early-stage cancers and advanced-stage cancers where available, as well as data for overall prevalence.

**Supplementary Table S5. Cancer stage categories**

| Cancer stage category | Different study-specific definitions |
|---|---|
| **Early-stage** | • Stage I, II, III (any of these and their combinations)<br>• Early-stage cancer<br>• For studies of brain cancer: Grade 1 or 2 [low grade] |
| **Advanced-stage** | • Stage III/IV<br>• Stage IV<br>• Locally advanced cancer<br>• Advanced cancer<br>• Metastatic cancer<br>• Relapsed and/or refractory cancer<br>• For studies of brain cancer: Grade 3 or 4 [high grade] |
| **Overall** | • Not limited to a specific cancer stage, nor restricted to early-stage or advanced-stage cancers |

**Supplementary Table S6. Minimum sample size thresholds for inclusion of studies in meta-analyses**

| Cancer type | Minimum sample size | | |
|---|---|---|---|
| | **Overall** | **Early-stage** | **Advanced-stage** |
| Colorectal cancer | 1000 | 400 | 200 |
| Colon cancer | 200 | 200 | 200 |
| Endometrial cancer | 400 | 50 | 50 |
| Lung cancer | 200 | 50 | 50 |
| Ovarian cancer | 100 | 50 | 50 |
| Gastric cancer | 400 | 50 | 50 |
| Other cancers | 50 | 50 | 50 |

## 4. Overview of literature reporting the prevalence of dMMR/MSI/high TMB

**Supplementary Fig. S1. Relative proportion of studies reporting the prevalence of high TMB *vs* dMMR/MSI within each tumour group**

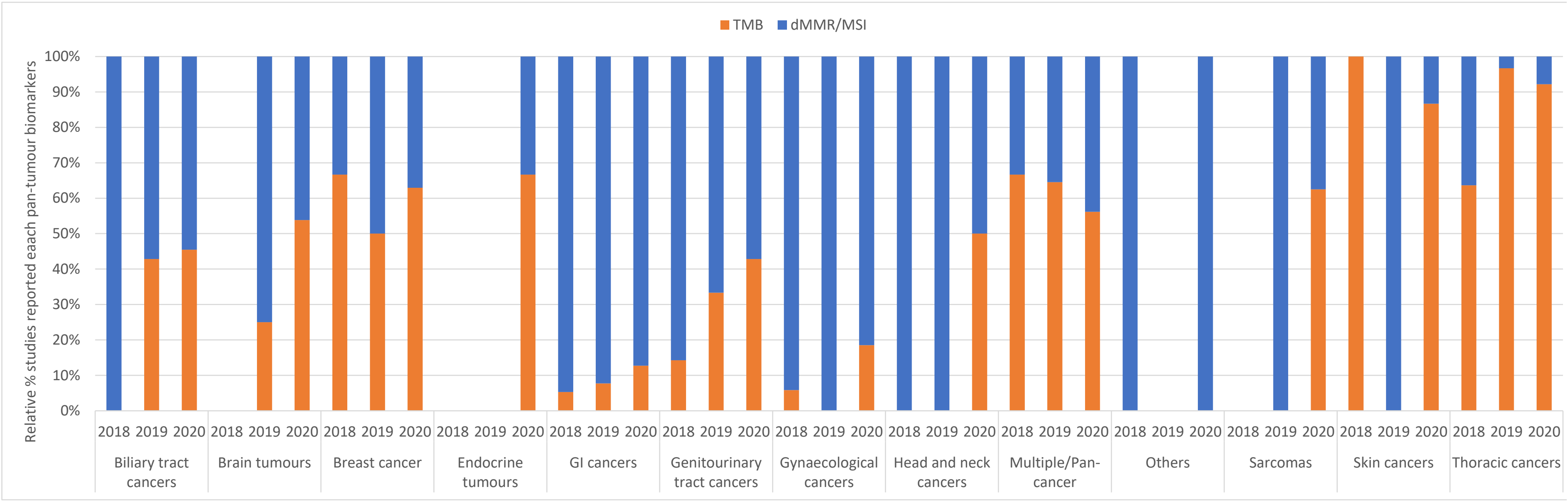


dMMR = mismatch repair deficiency; MSI = microsatellite instability; TMB = tumour mutational burden. See Supplementary Table S4 for cancer types included in each tumour group.

## 5. Detailed results

### 1) Number of prevalence estimates included in each cancer-specific meta-analysis

**Supplementary Table S7. Number of prevalence estimates included in each cancer-specific meta-analysis**

| Cancer | No. of prevalence estimates by pan-tumour biomarker | | | | | |
|---|---|---|---|---|---|---|
| | dMMR | MSI | TMB≥10 mut/Mb | TMB≥20 mut/Mb | dMMR/ MSI[a] | Total (column %) |
| ***Gastrointestinal cancers (170/412, 41%)*** | | | | | | |
| Anal cancer | - | 2 | 1 | 1 | - | **4 (1%)** |
| Appendiceal cancer | 1 | 3 | - | 1 | - | **5 (1%)** |
| Colon cancer[b] | 10 | 7 | - | 1 | 1 | **19 (5%)** |
| CRC[b] | 14 | 22 | 1 | 1 | 7 | **45 (11%)** |
| Esophageal cancer | 3 | 3 | 1 | 1 | - | **8 (2%)** |
| Gastric cancer | 17 | 24 | 3 | 2 | - | **46 (11%)** |
| Liver cancer | 1 | 6 | 3 | 1 | - | **11 (3%)** |
| Pancreatic cancer | 3 | 4 | 1 | 1 | - | **9 (2%)** |
| Rectal cancer[b] | 4 | 3 | - | - | 1 | **8 (2%)** |
| Small bowel cancer | 7 | 6 | 1 | 1 | - | **15 (4%)** |
| ***Genitourinary tract cancers (51/412, 12%)*** | | | | | | |
| Bladder/urothelial cancer | 6 | 6 | 6 | 6 | - | **24 (6%)** |
| Kidney cancer | 1 | 4 | - | 1 | - | **6 (1%)** |
| Penile cancer | 1 | 1 | - | - | - | **2 (0%)** |
| Prostate cancer | 6 | 5 | 3 | 1 | - | **15 (4%)** |
| Testicular cancer | 1 | 1 | 1 | 1 | - | **4 (1%)** |
| ***Breast and gynaecological cancers (69/412, 17%)*** | | | | | | |
| Breast cancer | 3 | 5 | 6 | 2 | - | **16 (4%)** |
| Cervical cancer | 1 | 2 | 2 | 1 | - | **6 (1%)** |
| Endometrial cancer (EC) | 14 | 7 | 2 | 2 | - | **25 (6%)** |
| Ovarian cancer | 11 | 6 | 2 | - | - | **19 (5%)** |
| Uterine cancer (excl. EC) | - | 1 | - | 1 | - | **2 (0%)** |
| Vulvar cancer | - | - | 1 | - | - | **1 (0%)** |
| ***Thoracic cancers (28/412, 7%)*** | | | | | | |
| Lung cancer | 4 | 6 | 13 | 3 | - | **26 (6%)** |
| Thymic malignancy | 1 | 1 | - | - | - | **2 (0%)** |
| ***Biliary tract cancers (17/412, 4%)*** | | | | | | |
| Ampullary cancer | 1 | - | - | - | - | **1 (0%)** |
| Bile duct/gallbladder | 3 | 9 | 2 | 2 | - | **16 (4%)** |
| ***Head and neck cancers, sarcomas, and skin cancers (39/412, 9%)*** | | | | | | |
| Head and neck cancers | 4 | 3 | 3 | 2 | - | **12 (3%)** |
| Sarcomas | 2 | 5 | 3 | 1 | - | **11 (3%)** |
| Skin cancers | 5 | 4 | 3 | 4 | - | **16 (4%)** |
| ***CNS tumours, endocrine tumours, neuroendocrine tumours, and other cancers (38/412, 9%)*** | | | | | | |
| Brain tumours | 6 | 3 | 1 | 3 | - | **13 (3%)** |
| Endocrine tumours | 1 | 3 | 2 | 1 | - | **7 (2%)** |
| Neuroendocrine tumours | 1 | 3 | 2 | 1 | - | **7 (2%)** |
| Other cancers[c] | 3 | 5 | - | 3 | - | **11 (3%)** |
| **Total (row %)** | **135 (33%)** | **160 (39%)** | **63 (15%)** | **45 (11%)** | **9 (2%)** | **412 (100%)** |

MMR = mismatch repair deficiency; MSI = microsatellite instability; TMB = tumour mutational burden; CRC = colorectal cancer; CNS = central nervous system.

[a] Pooled prevalence of dMMR and MSI in colorectal, colon and rectal cancers only using clinical trials and systematic review/meta-analysis given high concordance between dMMR and MSI in these cancer types.

[b] We analysed the pooled prevalence of pan-tumour biomarkers separately based on estimates including 1) colon cancer only, 2) rectal cancer only, and 3) tumours at any sites in the colon and the rectum, obtaining pooled prevalence estimates described as "colon cancer", "rectal cancer", and "colorectal cancer", respectively.

[c] Other caners include cancer of unknown primary, cancer of unknown primary-neuro, germ cell tumour, peritoneal cancer, and underspecified cancer.

**Supplementary Table S8. Numbers of prevalence estimates included in stage-specific meta-analyses**

| Biomarker | No. of prevalence estimates by stage group (row %) | | | Total |
|---|---|---|---|---|
| | Overall | Early-stage | Advanced stage | |
| dMMR | 93 (69%) | 28 (21%) | 14 (10%) | 135 (100%) |
| MSI | 108 (68%) | 29 (18%) | 23 (14%) | 160 (100%) |
| TMB≥10 mut/Mb | 32 (51%) | 2 (3%) | 29 (46%) | 63 (100%) |
| TMB≥20 mut/Mb | 33 (73%) | 1 (2%) | 11 (24%) | 45 (100%) |
| dMMR/MSI | 3 (33%) | 5 (56%) | 1 (11%) | 9 (100%) |
| **Total** | **269 (65%)** | **65 (16%)** | **78 (19%)** | **412 (100%)** |

MMR = mismatch repair deficiency; MSI = microsatellite instability; TMB = tumour mutational burden.

**Supplementary Table S9. Numbers of prevalence estimates included in cancer-specific meta-analyses by pan-tumour biomarker and assays used**

| Assay | No. of prevalence estimates by pan-tumour biomarker | | | | | Total |
|---|---|---|---|---|---|---|
| | dMMR | MSI | TMB≥10 mut/Mb | TMB≥20 mut/Mb | dMMR/MSI | |
| Gene panel sequencing | 28/136 (21%) | 77/160 (48%) | 41/63 (65%) | 42/45 (93%) | 0/9 (0%) | 188/413 (46%) |
| IHC | 101/135 (75%) | 0/160 (0%) | 0/63 (0%) | 0/45 (0%) | 0/9 (0%) | 102/413 (25%) |
| IHC/PCR | 0/136 (0%) | 0/160 (0%) | 0/63 (0%) | 0/45 (0%) | 8/9 (89%) | 8/413 (2%) |
| N/S | 3/136 (2%) | 5/160 (3%) | 1/63 (2%) | 2/45 (4%) | 1/9 (11%) | 12/413 (3%) |
| Others | 0/136 (0%) | 4/160 (3%) | 0/63 (0%) | 0/45 (0%) | 0/9 (0%) | 4/413 (1%) |
| PCR | 0/136 (0%) | 63/160 (39%) | 0/63 (0%) | 0/45 (0%) | 0/9 (0%) | 63/413 (15%) |
| WES | 3/136 (2%) | 10/160 (6%) | 20/63 (32%) | 1/45 (2%) | 0/9 (0%) | 34/413 (8%) |
| WGS | 0/136 (0%) | 1/160 (1%) | 1/63 (2%) | 0/45 (0%) | 0/9 (0%) | 2/413 (0%) |
| **Total** | **135/135 (100%)** | **160/160 (100%)** | **63/63 (100%)** | **45/45 (100%)** | **9/9 (100%)** | **412/412 (100%)** |

MMR = mismatch repair deficiency; MSI = microsatellite instability; TMB = tumour mutational burden; IHC = immunohistochemistry; PCR = polymerase chain reaction; WES = whole exome sequencing; WGS = whole genome sequencing.

**Supplementary Table S10. References of studies included in each cancer-specific meta-analysis of dMMR prevalence**

| | Overall | | Early stage | | Advanced stage | |
|---|---|---|---|---|---|---|
| Cancer | No. of estimate | References | No. of estimate | References | No. of estimate | References |
| ***Gastrointestinal cancers*** | | | | | | |
| Anal cancer | - | | - | | - | |
| Appendiceal cancer | 1 | 16 | - | | - | |
| Colon cancer[b] | 2 | 17, 18 | 7 | 19-25 | 1 | 26 |
| CRC[b] | 12 | 16, 27-35 | 1 | 35 | 1 | 36 |
| Esophageal cancer | 3 | 37-39 | - | | - | |
| Gastric cancer | 7 | 39-45 | 5 | 46-50 | 5 | 40, 41, 50-52 |
| Liver cancer | 1 | 16 | - | | - | |
| Pancreatic cancer | 2 | 16, 53 | - | | 1 | 54 |
| Rectal cancer[b] | - | | 4 | 55-58 | - | |
| Small bowel cancer | 6 | 16, 59-63 | 1 | 60 | - | |
| ***Genitourinary tract cancers*** | | | | | | |
| Bladder/urothelial cancer | 6 | 16, 64-68 | - | | - | |
| Kidney cancer | 1 | 16 | - | | - | |
| Penile cancer | 1 | 69 | - | | - | |
| Prostate cancer | 5 | 16, 67, 70-72 | - | | 1 | 73 |
| Testicular cancer | - | | 1 | 74 | - | |
| ***Breast and gynaecological cancers*** | | | | | | |
| Breast cancer | 3 | 16, 67, 75 | - | | - | |
| Cervical cancer | 1 | 16 | - | | - | |
| Endometrial cancer | 8 | 16, 76-82 | 6 | 78, 80, 83-86 | - | |
| Ovarian cancer[a] | 9 | 16, 42, 87-93 | 2 | 90, 94 | - | |
| Uterine cancer (excl. endometrial cancer) | - | | - | | - | |
| Vulvar cancer | - | | - | | - | |
| ***Thoracic cancers*** | | | | | | |
| Lung cancer | 4 | 16 | - | | - | |
| Thymic malignancy | 1 | 16 | - | | - | |
| ***Biliary tract cancers*** | | | | | | |
| Ampullary cancer | - | | 1 | 95 | - | |
| Bile duct/gallbladder | 3 | 16, 96, 97 | - | | - | |
| ***Head and neck cancers, sarcomas, and skin cancers*** | | | | | | |
| Head and neck cancers | 4 | 16, 98-100 | - | | - | |
| Sarcomas | 2 | 16 | - | | - | |
| Skin cancers | 4 | 16, 67, 101 | - | | 1 | 102 |
| ***CNS tumours, endocrine tumours, neuroendocrine tumours, and other cancers*** | | | | | | |
| Brain tumours | 2 | 16, 103 | - | | 4 | 104-107 |
| Endocrine tumours | 1 | 16 | - | | - | |
| Neuroendocrine tumours | 1 | 16 | - | | - | |
| Other cancers[b] | 3 | 16, 108 | - | | - | |

dMMR = mismatch repair deficiency; MSI = microsatellite instability.

[a] Including studies included in cancer sub-group analysis only (i.e., dMMR/MSI enriched histologic sub-types, e.g., endometroid carcinoma, non-serous carcinoma, mucinous carcinoma)

[b] We analysed the pooled prevalence of pan-tumour biomarkers separately based on estimates including 1) colon cancer only, 2) rectal cancer only, and 3) tumours at any sites in the colon and the rectum, obtaining pooled prevalence estimates described as "colon cancer", "rectal cancer", and "colorectal cancer", respectively.

[c] Other caners include cancer of unknown primary, cancer of unknown primary-neuro, germ cell tumour, peritoneal cancer, and underspecified cancer.

**Supplementary Table S11. References of studies included in each cancer-specific meta-analysis of MSI prevalence**

| | Overall | | Early stage | | Advanced stage | |
|---|---|---|---|---|---|---|
| Cancer | No. of estimate | References | No. of estimate | References | No. of estimate | References |
| ***Gastrointestinal cancers*** | | | | | | |
| Anal cancer | 2 | 109, 110 | - | | - | |
| Appendiceal cancer | 2 | 110, 111 | - | | 1 | 111 |
| Colon cancer[b] | 1 | 112 | 6 | 24, 113-117 | - | |
| CRC[b] | 11 | 32, 109, 110, 118-125 | 4 | 118, 119, 126, 127 | 7 | 118, 119, 128-132 |
| Esophageal cancer | 3 | 42, 109, 110 | - | | - | |
| Gastric cancer | 10 | 42, 109, 110, 121, 123, 133-137 | 11 | 138-148 | 3 | 149-151 |
| Liver cancer | 3 | 109, 121, 123 | - | | 3 | 123, 152, 153 |
| Pancreatic cancer | 4 | 109, 110, 154, 155 | - | | - | |
| Rectal cancer[b] | - | | 3 | 55, 156, 157 | - | |
| Small bowel cancer | 4 | 109, 110, 158, 159 | 2 | 160, 161 | - | |
| ***Genitourinary tract cancers*** | | | | | | |
| Bladder/urothelial cancer | 5 | 67, 68, 109, 110, 162 | - | | 1 | 163 |
| Kidney cancer | 3 | 109, 110, 164 | - | | 1 | 165 |
| Penile cancer | 1 | 69 | - | | - | |
| Prostate cancer | 4 | 67, 109, 110, 166 | - | | 1 | 167 |
| Testicular cancer | - | | - | | 1 | 168 |
| ***Breast and gynaecological cancers*** | | | | | | |
| Breast cancer | 4 | 67, 109, 110, 123 | - | | 1 | 169 |
| Cervical cancer | 2 | 110, 170 | - | | - | |
| Endometrial cancer | 5 | 80, 81, 110, 121, 171 | 1 | 80 | 1 | 172 |
| Ovarian cancer[a] | 6 | 42, 90, 109, 110, 173, 174 | - | | - | |
| Uterine cancer (excl. endometrial cancer) | 1 | 110 | - | | - | |
| Vulvar cancer | - | | - | | - | |
| ***Thoracic cancers*** | | | | | | |
| Lung cancer | 5 | 109, 110, 123 | - | | 1 | 123 |
| Thymic malignancy | 1 | 109 | - | | - | |
| ***Biliary tract cancers*** | | | | | | |
| Ampullary cancer | - | | - | | - | |
| Bile duct/gallbladder | 8 | 97, 110, 175-178 | - | | 1 | 179 |
| ***Head and neck cancers, sarcomas, and skin cancers*** | | | | | | |
| Head and neck cancers | 3 | 109, 110, 123 | - | | - | |
| Sarcomas | 4 | 67, 109, 110 | 1 | 180 | - | |
| Skin cancers | 4 | 67, 109, 110 | - | | - | |
| ***CNS tumours, endocrine tumours, neuroendocrine tumours, and other cancers*** | | | | | | |
| Brain tumours | 1 | 109 | 1 | 109 | 1 | 109 |
| Endocrine tumours | 3 | 109, 110, 181 | - | | - | |
| Neuroendocrine tumours | 3 | 109, 110 | - | | - | |
| Other cancers[c] | 5 | 109, 110 | - | | - | |

dMMR = mismatch repair deficiency; MSI = microsatellite instability.

[a] Including studies included in cancer sub-group analysis only (i.e., dMMR/MSI enriched histologic sub-types, e.g., endometroid carcinoma, non-serous carcinoma, mucinous carcinoma)

[b] We analysed the pooled prevalence of pan-tumour biomarkers separately based on estimates including 1) colon cancer only, 2) rectal cancer only, and 3) tumours at any sites in the colon and the rectum, obtaining pooled prevalence estimates described as “colon cancer”, “rectal cancer”, and “colorectal cancer”, respectively.

[c] Other caners include cancer of unknown primary, cancer of unknown primary-neuro, germ cell tumour, peritoneal cancer, and underspecified cancer.

**Supplementary Table S12. References of studies included in each cancer-specific meta-analysis of high TMB (≥10 mutations/Mb) prevalence**

| | Overall | | Early stage | | Advanced stage | |
|---|---|---|---|---|---|---|
| Cancer | No. of estimate | References | No. of estimate | References | No. of estimate | References |
| ***Gastrointestinal cancers*** | | | | | | |
| Anal cancer | - | | - | | 1 | 182 |
| Appendiceal cancer | - | | - | | - | |
| Colon cancer[b] | - | | - | | - | |
| CRC[b] | 1 | 123 | - | | - | |
| Esophageal cancer | 1 | 183 | - | | - | |
| Gastric cancer | 1 | 123 | - | | 2 | 52, 151 |
| Liver cancer | 1 | 123 | - | | 2 | 123, 152 |
| Pancreatic cancer | 1 | 123 | - | | - | |
| Rectal cancer[b] | - | | - | | - | |
| Small bowel cancer | 1 | 159 | - | | - | |
| ***Genitourinary tract cancers*** | | | | | | |
| Bladder/urothelial cancer | 3 | 184-186 | 1 | 185 | 2 | 163, 185 |
| Kidney cancer | - | | - | | - | |
| Penile cancer | - | | - | | - | |
| Prostate cancer | 2 | 166, 187 | - | | 1 | 167 |
| Testicular cancer | - | | - | | 1 | 168 |
| ***Breast and gynaecological cancers*** | | | | | | |
| Breast cancer | 4 | 123, 184, 188, 189 | - | | 2 | 169, 188 |
| Cervical cancer | 1 | 184 | - | | 1 | 182 |
| Endometrial cancer | 1 | 171 | - | | 1 | 182 |
| Ovarian cancer[a] | 1 | 174 | - | | 1 | 173 |
| Uterine cancer (excl. endometrial cancer) | - | | - | | - | |
| Vulvar cancer | - | | - | | 1 | 182 |
| ***Thoracic cancers*** | | | | | | |
| Lung cancer | 4 | 123, 184, 190, 191 | 1 | 192 | 8 | 123, 182, 193-196 |
| Thymic malignancy | - | | - | | - | |
| ***Biliary tract cancers*** | | | | | | |
| Ampullary cancer | - | | - | | - | |
| Bile duct/gallbladder | 1 | 197 | - | | 1 | 182 |
| ***Head and neck cancers, sarcomas, and skin cancers*** | | | | | | |
| Head and neck cancers | 2 | 123, 184 | - | | 1 | 182 |
| Sarcomas | 3 | 123, 184 | - | | - | |
| Skin cancers | 1 | 184 | - | | 2 | 198, 199 |
| ***CNS tumours, endocrine tumours, neuroendocrine tumours, and other cancers*** | | | | | | |
| Brain tumours | 1 | 123 | - | | - | |
| Endocrine tumours | 1 | 184 | - | | 1 | 182 |
| Neuroendocrine tumours | 1 | 184 | - | | 1 | 182 |
| Other cancers[c] | - | | - | | - | |

dMMR = mismatch repair deficiency; MSI = microsatellite instability.

[a] Including studies included in cancer sub-group analysis only (i.e., dMMR/MSI enriched histologic sub-types, e.g., endometroid carcinoma, non-serous carcinoma, mucinous carcinoma)

[b] We analysed the pooled prevalence of pan-tumour biomarkers separately based on estimates including 1) colon cancer only, 2) rectal cancer only, and 3) tumours at any sites in the colon and the rectum, obtaining pooled prevalence estimates described as "colon cancer", "rectal cancer", and "colorectal cancer", respectively.

[c] Other caners include cancer of unknown primary, cancer of unknown primary-neuro, germ cell tumour, peritoneal cancer, and underspecified cancer.

**Supplementary Table S13. References of studies included in each cancer-specific meta-analysis of high TMB (≥20 mutations/Mb) prevalence**

| | Overall | | Early stage | | Advanced stage | |
|---|---|---|---|---|---|---|
| Cancer | No. of estimate | References | No. of estimate | References | No. of estimate | References |
| ***Gastrointestinal cancers*** | | | | | | |
| Anal cancer | 1 | 110 | - | | - | |
| Appendiceal cancer | 1 | 110 | - | | - | |
| Colon cancer[b] | 1 | | - | | - | |
| CRC[b] | 1 | | - | | - | |
| Esophageal cancer | 1 | 110 | - | | - | |
| Gastric cancer | 1 | 110 | - | | 1 | 151 |
| Liver cancer | - | | - | | 1 | 152 |
| Pancreatic cancer | 1 | 200 | - | | - | |
| Rectal cancer[b] | - | | - | | - | |
| Small bowel cancer | 1 | 110 | - | | - | |
| ***Genitourinary tract cancers*** | | | | | | |
| Bladder/urothelial cancer | 3 | 110, 185, 186 | 1 | 185 | 2 | 163, 185 |
| Kidney cancer | 1 | 110 | - | | - | |
| Penile cancer | - | | - | | - | |
| Prostate cancer | 1 | 110 | - | | - | |
| Testicular cancer | - | | - | | 1 | 168 |
| ***Breast and gynaecological cancers*** | | | | | | |
| Breast cancer | 1 | 110 | - | | 1 | 169 |
| Cervical cancer | 1 | 110 | - | | - | |
| Endometrial cancer | 1 | 110 | - | | 1 | 172 |
| Ovarian cancer[a] | - | | - | | - | |
| Uterine cancer (excl. endometrial cancer) | 1 | 110 | - | | - | |
| Vulvar cancer | - | | - | | - | |
| ***Thoracic cancers*** | | | | | | |
| Lung cancer | 2 | 110 | - | | 1 | 201 |
| Thymic malignancy | - | | - | | - | |
| ***Biliary tract cancers*** | | | | | | |
| Ampullary cancer | - | | - | | - | |
| Bile duct/gallbladder | 2 | 110 | - | | - | |
| ***Head and neck cancers, sarcomas, and skin cancers*** | | | | | | |
| Head and neck cancers | 2 | 110 | - | | - | |
| Sarcomas | 1 | 110 | - | | - | |
| Skin cancers | 2 | 110 | - | | 2 | 198, 199 |
| ***CNS tumours, endocrine tumours, neuroendocrine tumours, and other cancers*** | | | | | | |
| Brain tumours | 2 | 103, 110 | - | | 1 | 202 |
| Endocrine tumours | 1 | 110 | - | | - | |
| Neuroendocrine tumours | 1 | 110 | - | | - | |
| Other cancers[c] | 3 | 110 | - | | - | |

dMMR = mismatch repair deficiency; MSI = microsatellite instability.

[a] Including studies included in cancer sub-group analysis only (i.e., dMMR/MSI enriched histologic sub-types, e.g., endometroid carcinoma, non-serous carcinoma, mucinous carcinoma)

[b] We analysed the pooled prevalence of pan-tumour biomarkers separately based on estimates including 1) colon cancer only, 2) rectal cancer only, and 3) tumours at any sites in the colon and the rectum, obtaining pooled prevalence estimates described as "colon cancer", "rectal cancer", and "colorectal cancer", respectively.

[c] Other caners include cancer of unknown primary, cancer of unknown primary-neuro, germ cell tumour, peritoneal cancer, and underspecified cancer.

### 2) Prevalence of high TMB (≥20 mutations/Mb)

The pooled overall prevalence of high TMB at the ≥20 mutations/Mb threshold was high for skin (43.5%), colon (19.0%), bladder/urothelial (12.0%), endometrial (11.9%) and lung (9.0%) cancers. Considerable variation was found in the pooled overall prevalence of TMB (≥20 mutations/Mb) among gastrointestinal (range: 0.5%-19.0%), gynaecological (range: 3.7%-11.9%) and genitourinary tract cancers (range: 1.5%-12.0%) (Supplementary Table S14). The prevalence of high TMB (≥20 mutations/Mb) also differed by sub-types in skin cancers (54.6% in non-melanoma *vs* 32.9% in melanoma overall) and lung cancers (11.5% in non-small cell lung cancer *vs* 6.7% in small cell lung cancer overall) (Supplementary Table S18).

**Supplementary Table S14. Prevalence of high tumour mutational burden (≥20 mutations/Mb) in adult solid tumours by cancer type and stage group**

| | Prevalence from random-effect model (95% CI)[a] | | |
|---|---|---|---|
| **Cancer** | **Overall** | **Early stage** | **Advanced stage** |
| ***Gastrointestinal cancers*** | | | |
| Anal cancer | 3.9% (2.5-5.5%) | - | - |
| Appendiceal cancer | 2.0% (1.2-3.0%) | - | - |
| Colon cancer[c] | 19.0% (15.3-23.1%) | - | - |
| Colorectal cancer[c] | 5.1% (4.8-5.4%) | - | - |
| Esophageal cancer | 2.5% (2.1-3.0%) | - | - |
| Gastric cancer | 4.9% (4.2-5.6%) | - | 7.4% (1.6-16.2%) |
| Liver cancer | - | - | 0.8% (0.3-1.6%) |
| Pancreatic cancer | 0.5% (0.1-1.0%) | - | - |
| Rectal cancer[c] | - | - | - |
| Small bowel cancer | 6.1% (4.7-7.7%) | - | - |
| ***Genitourinary tract cancers*** | | | |
| Bladder/urothelial cancer | 12.0% (6.9-18.3%), $I^2$=0.93[b] | 22.7% (17.0-28.9%) | 12.3% (8.8-16.2%), $I^2$=0.47 |
| Kidney cancer | 1.5% (1.1-1.9%) | - | - |
| Penile cancer | - | - | - |
| Prostate cancer | 3.0% (2.7-3.5%) | - | - |
| Testicular cancer | - | - | 0.9% (0.0-4.0%) |
| ***Breast and gynaecological cancers*** | | | |
| Breast cancer | 2.6% (2.4-2.8%) | - | 2.2% (1.7-2.7%) |
| Cervical cancer | 6.1% (5.0-7.3%) | - | - |
| Endometrial cancer (EC) | 11.9% (11.1-12.8%) | - | 13.5% (6.5-22.4%) |
| Ovarian cancer | - | - | - |
| Uterine cancer (excl. EC) | 3.7% (2.7-4.9%) | - | - |
| Vulvar cancer | - | - | - |
| ***Thoracic cancers*** | | | |
| Lung cancer | 9.0% (4.9-14.2%), $I^2$=0.98[b] | - | 11.9% (10.8-13.0%) |
| Thymic malignancy | - | - | - |
| ***Biliary tract cancers*** | | | |
| Ampullary cancer | - | - | - |
| Bile duct/gallbladder | 1.6% (0.8-2.6%), $I^2$=0.87[b] | - | - |
| ***Head and neck cancers, sarcomas, and skin cancers*** | | | |
| Head and neck cancers | 7.7% (6.9-8.6%), $I^2$=0 | - | - |
| Sarcomas | 2.8% (2.3-3.3%) | - | - |
| Skin cancers | 43.5% (23.3-64.9%), $I^2$=0.99[b] | - | 19.7% (1.5-50.0%), $I^2$=0.93[b] |
| ***Central nervous system tumours, endocrine tumours, neuroendocrine tumours, and other cancers*** | | | |
| Brain tumours | 5.5% (1.4-11.9%), $I^2$=0.82[b] | - | 1.0% (0.0-4.3%) |
| Endocrine tumours | 1.7% (1.0-2.6%) | - | - |
| Neuroendocrine tumours | 3.5% (2.2-5.1%) | - | - |
| Other cancers[d] | 8.9% (6.0-12.2%), $I^2$=0.94[b] | - | - |

[a] Heterogeneity across studies was presented based on the point estimate of $I^2$ score and heterogeneity test, if ≥2 records were available for each cancer type and stage group.

[b] Statistically significant heterogeneity across studies ($p<0.05$).

[b] We analysed the pooled prevalence of pan-tumour biomarkers separately based on estimates including 1) colon cancer only, 2) rectal cancer only, and 3) tumours at any sites in the colon and the rectum, obtaining pooled prevalence estimates described as "colon cancer", "rectal cancer", and "colorectal cancer", respectively.

[d] Other caners include cancer of unknown primary, cancer of unknown primary-neuro, germ cell tumour, peritoneal cancer, and underspecified cancer.

Note: See Supplementary Table S10 and Supplementary Table S13 for the number of records included in the analysis and the references.

## 3) Sub-group analyses of the prevalence of dMMR/MSI/high TMB

**Supplementary Table S15. Prevalence of dMMR in adult solid tumours by cancer sub-type and stage group**

| Cancer sub-type | Prevalence from random-effects model (95% CI)[a] | | |
|---|---|---|---|
| | Overall | Early stage | Advanced stage |
| ***Gynaecological cancers*** | | | |
| Ovarian cancer (dMMR/MSI enriched sub-types)[c] | 11.8% (5.9-19.3%), $I^2$=0.87[b] | 13.7% (9.4-18.7%) | - |
| ***Thoracic cancers*** | | | |
| Mesothelioma | 0.8% (0.0-2.5%) | - | - |
| NSCLC | 1.2% (0.8-1.6%) | - | - |
| SCLC | 0.8% (0.0-3.5%) | - | - |
| ***Biliary tract cancers*** | | | |
| Cholangiocarcinoma | 6.3% (2.1-12.1%) | - | - |
| Gallbladder cancer | - | - | - |
| ***Head and neck cancers*** | | | |
| Nasopharyngeal carcinoma | 2.0% (0.0-5.8%) | - | - |
| Oral cavity carcinoma | 7.4% (4.6-10.7%) | - | - |
| Salivary gland carcinoma | 0.0% (0.0-3.3%) | - | - |
| ***Sarcomas*** | | | |
| Bone sarcoma | - | - | - |
| Soft tissue sarcoma | 1.2% (0.6-1.9%) | - | - |
| ***Skin cancers*** | | | |
| Melanoma | 6.7% (0.0-37.5%), $I^2$=0.98[b] | - | - |
| Non-melanoma | 2.0% (0.4-4.4%) | - | 9.1% (5.2-13.8%) |
| ***Endocrine tumours*** | | | |
| Adrenocortical cancers | - | - | - |
| Thyroid carcinoma | 0.7% (0.1-1.7%) | - | - |
| ***Neuroendocrine tumours*** | | | |
| Carcinoid | - | - | - |
| GI neuroendocrine tumour | 0.0% (0.0-1.2%) | - | - |
| ***Other cancers*** | | | |
| Cancer of unknown primary | 2.3% (1.3-3.6%) | - | - |
| Germ cell tumour | 0.0% (0.0-1.5%) | - | - |
| Peritoneal cancer | 6.3% (2.7-11.4%) | - | - |
| Underspecified | - | - | - |
| Cancer of unknown primary-neuro | - | - | - |

dMMR = mismatch repair deficiency; MSI = microsatellite instability; NSCLC = non-small cell lung cancer; SCLC = small cell lung cancer.

[a] Heterogeneity across studies was presented based on the point estimate of $I^2$ score and heterogeneity test, if ≥2 records were available for each cancer type and stage group.

[b] Statistically significant heterogeneity across studies (p<0.05).

[c] dMMR/MSI enriched histologic sub-types: endometroid carcinoma, non-serous carcinoma, mucinous carcinoma.

**Supplementary Table S16. Prevalence of MSI in adult solid tumours by cancer sub-type and stage group**

| | Prevalence from random-effects model (95% CI)[a] | | |
|---|---|---|---|
| **Cancer sub-type** | **Overall** | **Early stage** | **Advanced stage** |
| ***Gynaecological cancers*** | | | |
| Ovarian cancer (dMMR/MSI enriched sub-types)[c] | 14.4% (8.9-20.9%), $I^2$=0.5 | - | - |
| ***Thoracic cancers*** | | | |
| Mesothelioma | - | - | - |
| NSCLC | 0.5% (0.2-0.9%), $I^2$=0.92[b] | - | - |
| SCLC | 0.5% (0.3-0.8%), $I^2$=0 | - | - |
| ***Biliary tract cancers*** | | | |
| Cholangiocarcinoma | 1.8% (0.7-3.3%), $I^2$=0.72[b] | - | - |
| Gallbladder cancer | 1.4% (0.0-6.1%) | - | - |
| ***Head and neck cancers*** | | | |
| Nasopharyngeal carcinoma | - | - | - |
| Oral cavity carcinoma | - | - | - |
| Salivary gland carcinoma | - | - | - |
| ***Sarcomas*** | | | |
| Bone sarcoma | 0.3% (0.0-1.0%) | - | - |
| Soft tissue sarcoma | 2.6% (0.0-11.3%), $I^2$=0.96[b] | 1.4% (0.0-5.7%) | - |
| ***Skin cancers*** | | | |
| Melanoma | 0.0% (0.0-0.2%) | - | - |
| Non-melanoma | 1.9% (1.2-2.9%), $I^2$=0 | - | - |
| ***Endocrine tumours*** | | | |
| Adrenocortical cancers | 1.1% (0.0-4.5%) | - | - |
| Thyroid carcinoma | 1.9% (0.0-5.5%) | - | - |
| ***Neuroendocrine tumours*** | | | |
| Carcinoid | 0.4% (0.0-1.1%) | - | - |
| GI neuroendocrine tumour | 2.0% (1.0-3.3%) | - | - |
| ***Other cancers*** | | | |
| Cancer of unknown primary | 2.2% (1.0-3.8%), $I^2$=0.85[b] | - | - |
| Germ cell tumour | - | - | - |
| Peritoneal cancer | 0.3% (0.0-0.9%) | - | - |
| Underspecified | 1.8% (0.9-2.8%) | - | - |
| Cancer of unknown primary-neuro | 0.9% (0.4-1.4%) | - | - |

dMMR = mismatch repair deficiency; MSI = microsatellite instability; NSCLC = non-small cell lung cancer; SCLC = small cell lung cancer.

[a] Heterogeneity across studies was presented based on the point estimate of $I^2$ score and heterogeneity test, if ≥2 records were available for each cancer type and stage group.

[b] Statistically significant heterogeneity across studies (p<0.05).

[c] dMMR/MSI enriched histologic sub-types: endometroid carcinoma, non-serous carcinoma, mucinous carcinoma.

**Supplementary Table S17. Prevalence of high TMB (≥10 mutations/Mb) in adult solid tumours by cancer sub-type and stage group**

| | Prevalence from random-effects model (95% CI)[a] | | |
|---|---|---|---|
| **Cancer sub-type** | **Overall** | **Early stage** | **Advanced stage** |
| ***Gynaecological cancers*** | | | |
| Ovarian cancer (dMMR/MSI enriched sub-types)[c] | 11.6% (6.3-18.3%) | - | - |
| ***Thoracic cancers*** | | | |
| Mesothelioma | - | - | 1.2% (0.0-5.0%) |
| NSCLC | 33.6% (28.1-39.2%), $I^2$=0.88[b] | 58.7% (52.4-65.0%) | 42.3% (35.1-49.6%), $I^2$=0.92[b] |
| SCLC | - | - | 38.6% (29.1-48.6%), $I^2$=0.59 |
| ***Biliary tract cancers*** | | | |
| Cholangiocarcinoma | 9.5% (3.3-18.2%) | - | - |
| Gallbladder cancer | - | - | - |
| ***Head and neck cancers*** | | | |
| Nasopharyngeal carcinoma | - | - | - |
| Oral cavity carcinoma | - | - | - |
| Salivary gland carcinoma | - | - | 3.7% (0.5-9.1%) |
| ***Sarcomas*** | | | |
| Bone sarcoma | - | - | - |
| Soft tissue sarcoma | 1.3% (0.0-4.8%), $I^2$=0.57 | - | - |
| ***Skin cancers*** | | | |
| Melanoma | 52.6% (49.7-55.5%) | - | 28.0% (18.8-38.3%) |
| Non-melanoma | - | - | 58.2% (44.8-71.0%) |
| ***Endocrine tumours*** | | | |
| Adrenocortical cancers | - | - | - |
| Thyroid carcinoma | - | - | 2.5% (0.0-7.4%) |
| ***Neuroendocrine tumours*** | | | |
| Carcinoid | - | - | - |
| GI neuroendocrine tumour | - | - | - |
| ***Other cancers*** | | | |
| Cancer of unknown primary | - | - | - |
| Germ cell tumour | - | - | - |
| Peritoneal cancer | - | - | - |
| Underspecified | - | - | - |
| Cancer of unknown primary-neuro | - | - | - |

dMMR = mismatch repair deficiency; MSI = microsatellite instability; TMB = tumour mutational burden; NSCLC = non-small cell lung cancer; SCLC = small cell lung cancer.

[a] Heterogeneity across studies was presented based on the point estimate of $I^2$ score and heterogeneity test, if ≥2 records were available for each cancer type and stage group.

[b] Statistically significant heterogeneity across studies (p<0.05).

[c] dMMR/MSI enriched histologic sub-types: endometroid carcinoma, non-serous carcinoma, mucinous carcinoma.

**Supplementary Table S18. Prevalence of high TMB (≥20 mutations/Mb) in adult solid tumours by cancer sub-type and stage group**

| | Prevalence from random-effects model (95% CI)[a] | | |
|---|---|---|---|
| **Cancer sub-type** | **Overall** | **Early stage** | **Advanced stage** |
| ***Gynaecological cancers*** | | | |
| Ovarian cancer (dMMR/MSI enriched sub-types)[c] | - | - | - |
| ***Thoracic cancers*** | | | |
| Mesothelioma | - | - | - |
| NSCLC | 11.5% (11.2-11.8%) | - | 11.9% (10.8-13.0%) |
| SCLC | 6.7% (5.8-7.7%) | - | - |
| ***Biliary tract cancers*** | | | |
| Cholangiocarcinoma | 1.2% (0.8-1.5%) | - | - |
| Gallbladder cancer | - | - | - |
| ***Head and neck cancers*** | | | |
| Nasopharyngeal carcinoma | - | - | - |
| Oral cavity carcinoma | - | - | - |
| Salivary gland carcinoma | 7.1% (5.5-8.8%) | - | - |
| ***Sarcomas*** | | | |
| Bone sarcoma | - | - | - |
| Soft tissue sarcoma | 2.8% (2.3-3.3%) | - | - |
| ***Skin cancers*** | | | |
| Melanoma | 32.9% (31.7-34.1%) | - | 8.5% (3.3-15.7%) |
| Non-melanoma | 54.6% (51.4-57.8%) | - | 34.5% (22.5-47.7%) |
| ***Endocrine tumours*** | | | |
| Adrenocortical cancers | - | - | - |
| Thyroid carcinoma | - | - | - |
| ***Neuroendocrine tumours*** | | | |
| Carcinoid | - | - | - |
| GI neuroendocrine tumour | 3.5% (2.2-5.1%) | - | - |
| ***Other cancers*** | | | |
| Cancer of unknown primary | 8.7% (8.2-9.2%) | - | - |
| Germ cell tumour | - | - | - |
| Peritoneal cancer | - | - | - |
| Underspecified | 13.1% (10.8-15.6%) | - | - |
| Cancer of unknown primary-neuro | 5.7% (4.5-7.0%) | - | - |

dMMR = mismatch repair deficiency; MSI = microsatellite instability; TMB = tumour mutational burden; NSCLC = non-small cell lung cancer; SCLC = small cell lung cancer.

[a] Heterogeneity across studies was presented based on the point estimate of $I^2$ score and heterogeneity test, if ≥2 records were available for each cancer type and stage group.

[b] Statistically significant heterogeneity across studies (p<0.05).

[c] dMMR/MSI enriched histologic sub-types: endometroid carcinoma, non-serous carcinoma, mucinous carcinoma.

**Supplementary Table S19. Prevalence of dMMR/MSI/high TMB by tumour group, based on studies reporting prevalence estimates on tumour group level only**

| | Prevalence from random-effects model (95% CI)[a] | | |
|---|---|---|---|
| **Tumour group** | **dMMR** | **MSI** | **TMB (≥10 mutations/Mb)** |
| GI cancers[16, 67, 184] | 8.1% (0.8-21.6%), $I^2$=0.95[b] | | 6.8% (6.3-7.3%) |
| Genitourinary tract cancers[16] | | | 5.5% (4.6-6.4%) |
| Gynaecological cancers[67, 184] | 6.0% (3.1-9.7%) | 5.0% (2.3-8.5%) | 9.5% (8.8-10.3%) |
| Central nervous system tumours[16] | | | 5.4% (3.9-7.2%) |

dMMR = mismatch repair deficiency; MSI = microsatellite instability; TMB = tumour mutational burden.

[a] Heterogeneity across studies was presented based on the point estimate of $I^2$ score and heterogeneity test, if ≥2 records were available for each cancer type and stage group.

[b] Statistically significant heterogeneity across studies (p<0.05).

**Supplementary Table S20. Pooled prevalence of dMMR/MSI in colorectal, colon and rectal cancers**

| | Prevalence from random-effects model (95% CI)[a] | | |
|---|---|---|---|
| **Cancer[c]** | **Overall** | **Early stage** | **Advanced stage** |
| Colorectal cancer[203-208] | 8.4% (4.9-12.8%), $I^2$=0.98[b] | 17.0% (12.6-21.9%), $I^2$=0.99[b] | 9.7% (8.7-10.8%) |
| Colon cancer[209] | - | 11.9% (11.0-13.0%) | - |
| Rectal cancer[210] | - | 13.7% (12.9-14.6%) | - |

dMMR = mismatch repair deficiency; MSI = microsatellite instability.

[a] Heterogeneity across studies was presented based on the point estimate of $I^2$ score and heterogeneity test, if ≥2 records were available for each cancer type and stage group.

[b] Statistically significant heterogeneity across studies (p<0.05).

[c] We analysed the pooled prevalence of pan-tumour biomarkers separately based on estimates including 1) colon cancer only, 2) rectal cancer only, and 3) tumours at any sites in the colon and the rectum, obtaining pooled prevalence estimates described as "colon cancer", "rectal cancer", and "colorectal cancer", respectively.

Note: Pooled prevalence of dMMR and MSI in colorectal, colon and rectal cancers only using clinical trials and systematic review/meta-analysis given high concordance between dMMR and MSI in these cancer types.

## 4) Sensitivity analyses of the prevalence of dMMR/MSI/high TMB

**Supplementary Table S21. Prevalence of MSI/high TMB by assays used**

| | | | Prevalence from random-effects model (95% CI)[a] | | |
|---|---|---|---|---|---|
| **Pan-tumour biomarker** | **Cancer†** | **Stage** | **PCR** | **Selected gene panel sequencing** | **Whole exome sequencing** |
| MSI | Colorectal cancer[109, 110, 118-122, 124, 125, 127, 129-131] | Overall | 11.7% (9.0-14.8%), $I^2$=0.97[b] | 6.9% (4.3-10.0), $I^2$=0.98[b] | - |
| MSI | Gastric cancer[42, 109, 110, 121, 134-137] | Overall | 9.7% (6.2-13.9%), $I^2$=0.95[b] | 5.5% (3.9-7.4), $I^2$=0.72[b] | -- |
| High TMB (≥10 mut/MB) | Lung cancer[123, 182, 193, 195, 196] | Advanced | - | 32.7% (17.9-49.5%), $I^2$=0.96[b] | 22.6% (5.3-47.2%), $I^2$=0.98[b] |

MSI = microsatellite instability; TMB = tumour mutational burden.

a Heterogeneity across studies was presented based on the point estimate of $I^2$ score and heterogeneity test, if ≥2 records were available for each cancer type and stage group.

[b] Statistically significant heterogeneity across studies (p<0.05).

Note: Cancer types with ≥3 records per each pan-tumour biomarker and assay were included in the analysis.

## 5) Publication bias

**Supplementary Fig. S2. Funnel plots for meta-analyses pooling 10+ estimates, showing the transformed proportions and their standard errors. a) Overall prevalence of dMMR and MSI in colorectal cancer and MSI in gastric cancer; b) Prevalence of MSI in early-stage gastric cancer**

**a)**

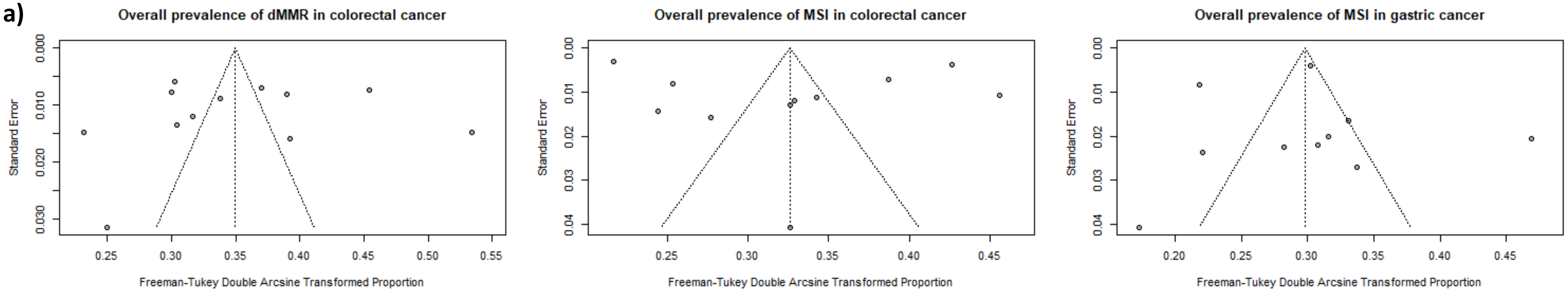


**b)**

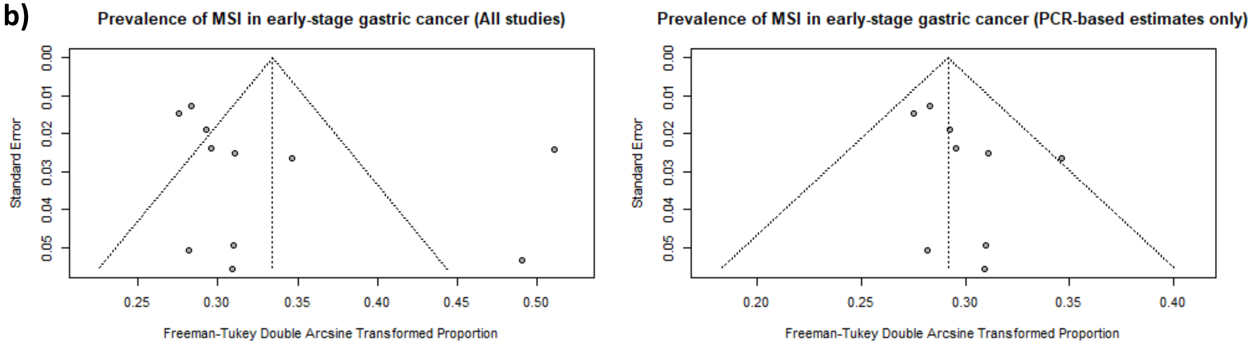


The funnel plots show the Freeman-Tukey double arcsine transformed prevalence. The vertical and diagonal dashed lines represent the pooled estimate for the transformed proportion and the corresponding 95% confidence interval, respectively. Each point represents the estimate from one study.

## 6) Illustrative example: estimated numbers of gastric and lung cancer patients with dMMR and, separately, high TMB tumours for two cancer types in Australia in 2021

**Supplementary Table S22. Estimated numbers of gastric and lung cancer patients with dMMR and, separately, with high TMB (≥10 mutations/Mb) tumours in Australia in 2021**

| Cancer type | Incidence (Estimated in 2021 in Australia)[211] | % distant cases[212] | Mismatch repair deficiency | | | High tumour mutational burden (≥10 mutations/Mb) | | |
|---|---|---|---|---|---|---|---|---|
| | | | Overall prevalence [a] | Estimated no. of all cases (Australia 2021) | Estimated no. of distant cases (Australia 2021) | Overall prevalence[*] | Estimated no. of all cases (Australia 2021) | Estimated no. of distant cases (Australia 2021) |
| Gastric cancer | 2,392 | 38.0% | 8.7% | 208 | 79 | 13.9% | 332 | 126 |
| Lung cancer | 13,810 | 53.6% | 1.6% | 221 | 118 | 25.7% | 3,549 | 1,902 |

[a] Overall prevalence from random-effect model in our study.

## 6. Comparison with prior reviews reporting the prevalence of dMMR/MSI/high TMB

Past reviews of dMMR/MSI prevalence have typically focused on a single cancer type, with only two existing structured/systematic reviews consolidating evidence for multiple different cancers.[42, 213]

Lorenzi et al. reported the pooled prevalence of dMMR and MSI across all solid tumours and for six selected tumour types (endometrial, ovarian, gastric, colorectal, esophageal and renal cancers).[42] A systematic search was performed for all studies reporting the prevalence of dMMR or MSI by immunohistochemistry or polymerase chain replication techniques on 26/10/2017, with targeted hand searches for colorectal cancer and pan-tumour genomic studies. A total of 156 studies were included in this structured review, including 103 studies reporting the prevalence of MSI, 48 studies reporting the prevalence of dMMR, and 5 pan-tumour studies. The included studies mostly focused on endometrial (n=53), gastric (n=39), ovarian (n=23), renal (n=9), esophageal (n=6) and colorectal (n=12) cancers. The pooled prevalence of dMMR across the 13 tumour types was 16% (95% CI: 11%-22%) and the pooled prevalence of MSI across 25 tumours was 14% (95% CI: 10%-19%). These pooled prevalence estimates are substantially higher than the pooled pan-cancer prevalence estimates in the current scoping review (dMMR: 2.9% [95% CI: 2.7%-3.1%]; and MSI: 2.7% [95% CI: 2.1%-3.4%]), which is likely due to almost two-thirds of the studies in the previous review focusing on colorectal, endometrial and stomach cancers (where dMMR and MSI are more common). For stage I/II, III and IV cancers, the pooled prevalence estimated in the previous review was 15% (95% CI: 8%–23%), 9% (95% CI: 3%–17%) and 3% (95% CI: 1%–7%), respectively.

Luchini et al. carried out a systematic review of studies that reported test results for high TMB, MSI/dMMR (which were not explicitly differentiated in that work), and PD-1/PD-L1 expression in the same samples, identifying 18 studies on 17 cohorts published to September 2018, of which 10 cohorts were focused on specifically on cancers of the digestive system. In total, the review summarised that a total of 4186 patients from all cohorts combined were positive for at least one of the three biomarkers, and 2.9% of these patients had tumours with high TMB, MSI, and PD-1/PD-L1 expression. Based on Figure 1, there were a total of 913 patients (21.8% of 4186) whose tumours exhibited MSI/dMMR or high TMB, of whom 45.9% exhibited both MSI/dMMR and high TMB, while 42.2% exhibited high TMB only and 11.9% exhibited MSI/dMMR only. However, there were differences in concordance of individual-level biomarker presence between different cancer types, so these results would also be influenced by the cancer type composition of the cohorts underlying the estimates.

Luchini et al. also reported the pooled cancer-specific prevalence of dMMR/MSI (which were not explicitly differentiated in that work) for 14 different cancer types based on six systematic reviews/meta-analyses on MSI published to 15 September 2018, the ESMO factsheet plus one additional study from a hand-search.[213] This study was performed as part of the collaborative project by the ESMO Translational Research and Precision Medicine Working Group, to generate consensus recommendations on the definitions of dMMR/MSI, methods of dMMR/MSI testing, and relationships between MSI, TMB and PD-1/PD-L1 expression. The estimated cancer-specific pooled overall prevalence for the 14 included cancer types was generally similar to our meta-analyses (see Supplementary Table S23).

**Supplementary Table S23. Comparison of the prevalence of dMMR/MSI between a prior systematic review and the current analysis**

| | Luchini et al.[213] | Current analysis | |
|---|---|---|---|
| Cancer type(s) | Overall prevalence of dMMR/ MSI | Overall prevalence of dMMR (95% CI) | Overall prevalence of MSI (95% CI) |
| Colorectal cancer | 17% | 11.7% (9.3-14.4%) | 10.2% (6.6-14.5%) |
| Endometrial cancer | 20% | 26.8% (23.3-30.5%) | 21.9% (15.1-29.6%) |
| Gastric-esophageal cancer | 13% | Gastric: 8.7% (7.6-9.9%)<br>Esophageal: 3.8% (1.1-7.8%) | Gastric: 8.5% (6.4-10.9%)<br>Esophageal: 2.4% (1.1-4.2%) |
| Small bowel cancer | 8.3% | 21.0% (15.8-26.7%) | 14.3% (5.4-26.3%) |
| Ovarian cancer | 3.5%-10% | 2.4% (0.5-5.5%) | 1.7% (0.0-5.4%) |
| Glioblastoma | 6%-13% | 5.1% (3.0-7.7%) | 0.6% (0.2-1.3%) |
| Cancer of unknown primary | 1.8% | 2.3% (1.3-3.6%) | 2.2% (1.0-3.8%) |
| Cervical cancer | 4% | 1.9% (0.0-5.8%) | 1.5% (0.7-2.6%) |
| Extrahepatic bile duct cancer | 3.4% | 3.8% (1.5-7.0%) | 1.6% (1.0-2.3%) |
| Pancreatic cancer | 1%-7% | 1.5% (0.6-2.7%) | 0.9% (0.4-1.5%) |
| Non-small-cell lung cancer | <1% | 1.2% (0.8-1.6%) | 0.5% (0.2-0.9%) |
| Head and neck cancer | <1% | 2.2% (0.1-6.1%) | 0.5% (0.3-0.7%) |
| Sarcomas | 2% | 0.5% (0.0-2.1%) | 1.8% (0.0-6.0%) |

dMMR = mismatch repair deficiency; MSI = microsatellite instability; CI = confidence interval.

## 7. Studies reporting the overlap between high TMB and dMMR/MSI status in individual tumours

Of the 85 studies reporting the prevalence of both high TMB and at least one of dMMR/MSI, we identified 17 studies reporting the overlap between high TMB (at different cut-offs) and dMMR/MSI status in individual tumours, including five studies reporting the overall overlap across all cancers and eight studies for gastrointestinal cancers (see Supplementary Table S24). In particular, Vanderwalde et al. reported the prevalence of MSI, high TMB (≥17 mutations/Mb) and PD-L1 in 11,348 cancer cases and the overlap between these pan-tumour biomarkers across all cancers and by 30+ different cancer types, with overlap depending on cancer types and generally high in gastrointestinal cancers (see Supplementary Table S25).[214] We did not identify any large-scale study reporting the overlap in individual tumours in 10+ cancer types based on high TMB cut-offs of ≥10 mutations/Mb or ≥20 mutations/Mb. We also did not identify any large-scale original study reporting the estimated overlap of all three pan-tumour biomarkers (dMMR, MSI, high TMB) in the same tumours, making this a key area for future research.

**Supplementary Table S24. Studies reporting the concordance between high TMB and dMMR/MSI status in individual tumours**

| Author (year) | Cancer type | Overlap between high TMB and dMMR/MSI status |
|---|---|---|
| ***High TMB (≥8 mutations/Mb)*** | | |
| Lee (2019)[215] | Colorectal cancer | • Of 66 tumours with high TMB and MSI, 42.4% exhibited both markers, while 40.9% exhibited high TMB only and 16.7% MSI only |
| ***High TMB (≥10 mutations/Mb)*** | | |
| Marabelle (2020)[182] | Pan-cancer (advanced incurable solid tumours) | • Phase 2 KEYNOTE-158 cohort<br>• Of 95 tumours with high TMB and/or MSI, 14.7% exhibited both markers, while 85.3% exhibited high TMB only and 0% MSI only |
| Huang (2020)[184] | Pan-cancer | • Of 48782 tumours, 34.2% was NSCLC (where high TMB without exhibiting dMMR/MSI is common)<br>• Of 10291 tumours with high TMB and/or MSI, 8.8% exhibited both markers, while 91.0% exhibited TMB only and 0.2% MSI only |
| Echejoh (2020)[216] | Colon cancer | • Of 16 tumours with high TMB, 81.3% exhibited both MSI and dMMR |
| Wirta (2020)[159] | Small bowel cancer | • Of 94 tumours with high TMB and/or MSI, 77.8% exhibited both markers, while 22.2% exhibited high TMB only and 0.0% MSI only |
| Jones (2020)[171] | Endometrial cancer | • Of 253 tumours with high TMB and/or MSI, 67.6% exhibited both markers, while 26.9% exhibited high TMB only and 5.5% MSI only |
| Abida (2019)[166] | Prostate cancer | • Of 47 tumours with high TMB and/or MSI, 48.9% exhibited both markers, while 51.1% exhibited high TMB only and 0.0% MSI only |
| ***High TMB (≥10.5 mutations/Mb)*** | | |
| Cho (2019)[217] | Gastric cancer | • Of 330 tumours with high TMB and/or MSI, 51.4% exhibited both markers, while 48.6% exhibited high TMB only and 0.0% MSI only |
| ***High TMB (≥12 mutations/Mb)*** | | |
| Fabrizio (2018)[218] | Colorectal cancer | • Of 466 tumours with high TMB and/or MSI, 64.6% exhibited both markers, while 35.2% exhibited high TMB only and 0.2% MSI only |
| ***High TMB (≥17 mutations/Mb)*** | | |
| Vanderwalde (2018)[214] | • Pan-cancer<br>• Cancer-specific | • Relationship between MSI, high TMB (≥17 mut/Mb) and PD-L1 was presented across all cancers and by cancer type (see |

<table>
<tr><th>Author (year)</th><th>Cancer type</th><th>Overlap between high TMB and dMMR/MSI status</th></tr>
<tr><td></td><td></td><td>Supplementary Table S25 for individual-level overlap between high TMB (≥17 mutations/Mb) and MSI tumour status)</td></tr>
<tr><td>Nikanjam (2020)[109]</td><td>Pan-cancer</td><td>• Overlap between MSI, high TMB (≥17 mut/Mb) and PD-L1 across all cancers<br>• Of 2603 tumours with high TMB and/or MSI, 25.4% exhibited both markers, while 64.5% exhibited high TMB only and 10.1% MSI only</td></tr>
<tr><td>Salem (2018)[219]</td><td>Gastrointestinal cancers (across 14 cancer types)</td><td>• Of 209 tumours with high TMB and/or MSI, 64.1% exhibited both markers, while 27.3% exhibited high TMB only and 8.6% MSI only</td></tr>
<tr><td>Weinberg (2018)[220]</td><td>Gastrointestinal cancers</td><td>• Of 47 tumours with high TMB and/or MSI, 70.2% exhibited both markers, while 14.9% exhibited high TMB only and 14.9% MSI only</td></tr>
<tr><td colspan="3">High TMB (≥20 mutations/Mb)</td></tr>
<tr><td>Harthimmer (2019)[221]</td><td>Ampullary carcinoma</td><td>• Of the 4 samples with MSI, 100% exhibited high TMB (≥20 mut/Mb)</td></tr>
<tr><td>Singhi (2019)[200]</td><td>Pancreatic cancer</td><td>• Of 6 tumours with high TMB and/or MSI, 33.3% exhibited both markers, while 50.0% exhibited high TMB only and 16.7% MSI only</td></tr>
<tr><td>Chung (2019)[187]</td><td>Prostate cancer</td><td>• Of 11 tumours with high TMB, 71.2% exhibited MSI</td></tr>
<tr><td colspan="3">High TMB (≥17 mutations/Mb or ≥20 mutations/Mb)</td></tr>
<tr><td>Luchini (2019)[213]</td><td>Multiple cancer types</td><td>• Systematic review of the association between MSI, TMB and PD-L1, including Vanderwalde et al.[214], Salem et al.[219] and Weinberg et al.[220]
<table>
<tr><th rowspan="2">Cancer</th><th>Of all cases exhibiting any of MSI, high TMB, and/or PD-L1 expression</th><th colspan="3">Of all cases exhibiting both MSI and/or high TMB</th></tr>
<tr><th>MSI and/or high TMB</th><th>Both MSI and high TMB</th><th>MSI only</th><th>high TMB only</th></tr>
<tr><td>All cancers</td><td>21.8%</td><td>45.9%</td><td>11.9%</td><td>42.2%</td></tr>
<tr><td>CRC</td><td>54.6%</td><td>81.0%</td><td>3.1%</td><td>15.9%</td></tr>
<tr><td>EC</td><td>63.4%</td><td>60.9%</td><td>31.4%</td><td>7.7%</td></tr>
<tr><td>EGA</td><td>36.5%</td><td>75.9%</td><td>14.0%</td><td>10.1%</td></tr>
<tr><td>Melanoma</td><td>29.1%</td><td>0.0%</td><td>0.0%</td><td>100.0%</td></tr>
<tr><td>NSCLC</td><td>11.5%</td><td>9.6%</td><td>0.0%</td><td>90.4%</td></tr>
</table></td></tr>
</table>

dMMR = mismatch repair deficiency; MSI = microsatellite instability; MSS = microsatellite stables; TMB = tumour mutational burden; EC = endometrial cancer; EGA = esophagogastric adenocarcinoma; NSCLC = non-small cell lung cancer.

**Supplementary Table S25. Individual-level overlap between high TMB (≥17 mutations/Mb) and MSI tumour status as reported in Vanderwalde et al. (Cancer Medicine 2018)**

| Tumour group | Cancer type | Overlap % (n/N)[a] |
|---|---|---|
| All cancer types | All cancer types | 24.5% (240/979) |
| Biliary tract cancers | Cholangiocarcinoma | 42.9% (3/7) |
| | Extrahepatic bile duct adenocarcinoma | 100.0% (1/1) |
| Breast and gynaecological cancers | Breast carcinoma | 12.1% (4/33) |
| | Cervical cancer | 11.8% (2/17) |
| | Endometrial carcinoma | 50.6% (89/176) |
| | Nonepithelial ovarian cancer | 0.0% (0/1) |
| | Ovarian surface epithelial carcinomas | 46.4% (13/28) |
| | Other female genital tract malignancies | 25.0% (1/4) |
| Central nervous system tumours | Glioblastoma | 20.0% (3/15) |
| | Low-grade glioma | 0.0% (0/1) |
| Endocrine tumours | Thyroid carcinoma | 100.0% (1/1) |
| Gastrointestinal cancers | Colorectal adenocarcinoma | 78.4% (76/97) |
| | Esophageal and esophagogastric junction carcinoma | 0.0% (0/1) |
| | Gastric adenocarcinoma | 88.2% (15/17) |
| | Liver hepatocellular carcinoma | 50.0% (1/2) |
| | Pancreatic adenocarcinoma | 50.0% (4/8) |
| | Small intestinal malignancies | 71.4% (5/7) |
| Genitourinary tract cancers | Bladder cancer | 0.0% (0/24) |
| | Kidney cancer | 0.0% (0/2) |
| | Prostatic adenocarcinoma | 80.0% (4/5) |
| Head and neck cancers | Head and neck squamous carcinoma | 0.0% (0/6) |
| Neuroendocrine tumours | Neuroendocrine tumours | 27.3% (3/11) |
| Sarcomas | Soft tissue tumours | 0.0% (0/12) |
| | Uterine sarcoma | 20.0% (1/5) |
| Skin cancers | Melanoma | 0.0% (0/126) |
| | Merkel cell carcinoma | 0.0% (0/2) |
| | Uveal melanoma | 100.0% (1/1) |
| Thoracic cancers | Non-small cell lung cancer | 3.4% (9/267) |
| | Small cell lung cancer | 0.0% (0/5) |
| | Thymic carcinoma | 0.0% (0/1) |

MSI = microsatellite instability; TMB = tumour mutational burden.

[a] Source: Cancer Medicine 2018. Vanderwalde et al.[214] Overlap was calculated by dividing the number of cases with both MSI and high TMB (≥17 mutations/Mb) by the total number of cases with MSI only, high TMB (≥17 mutations/Mb) only and both MSI and high TMB (≥17 mutations/Mb).

## 8. References


1. Pestana RC, Sen S, Hobbs BP, Hong DS. Histology-agnostic drug development - considering issues beyond the tissue. Nature reviews Clinical oncology. 2020;17(9):555-68.

2. Lemery S, Keegan P, Pazdur R. First FDA Approval Agnostic of Cancer Site - When a Biomarker Defines the Indication. The New England journal of medicine. 2017;377(15):1409-12.

3. The Pharmaceutical Benefit Scheme: Pembrolizumab: Australian Government. Department of Health; [Available from: https://www.pbs.gov.au/medicine/item/10424P-10436G-10475H-10493G-11330H-11352L-11492W-11494Y-11632F-11646Y-12119W-12120X-12121Y-12122B-12123C-12124D-12125E-12126F-12127G-12128H-12129J-12130K-12605K-12615Y.

4. About Health Technology Assessment: Australian Government. Department of Health; [updated 18/07/2019. Available from: https://www1.health.gov.au/internet/hta/publishing.nsf/Content/about-1.

5. Le DT, Durham JN, Smith KN, Wang H, Bartlett BR, Aulakh LK, et al. Mismatch repair deficiency predicts response of solid tumors to PD-1 blockade. Science (New York, NY). 2017;357(6349):409-13.

6. Buttner R, Longshore JW, Lopez-Rios F, Merkelbach-Bruse S, Normanno N, Rouleau E, et al. Implementing TMB measurement in clinical practice: Considerations on assay requirements. ESMO Open. 2019;4 (1) (no pagination)(e000442).

7. Doi SA, Barendregt JJ, Khan S, Thalib L, Williams GM. Advances in the meta-analysis of heterogeneous clinical trials I: The inverse variance heterogeneity model. Contemporary clinical trials. 2015;45(Pt A):130-8.

8. Tricco AC, Lillie E, Zarin W, O'Brien KK, Colquhoun H, Levac D, et al. PRISMA Extension for Scoping Reviews (PRISMA-ScR): Checklist and Explanation. Annals of internal medicine. 2018;169(7):467-73.

9. Pham T, Roth S, Kong J, Guerra G, Narasimhan V, Pereira L, et al. An Update on Immunotherapy for Solid Tumors: A Review. Ann Surg Oncol. 2018;25(11):3404-12.

10. Lynch HT, de la Chapelle A. Hereditary colorectal cancer. The New England journal of medicine. 2003;348(10):919-32.

11. Shirazi M, Sepulveda AR. Therapy Implications of DNA Mismatch Repair Deficiency, Microsatellite Instability, and Tumor Mutation Burden. Advances in Molecular Pathology. 2018;1(1):193-208.

12. Berera S, Koru-Sengul T, Miao F, Carrasquillo O, Nadji M, Zhang Y, et al. Colorectal Tumors From Different Racial and Ethnic Minorities Have Similar Rates of Mismatch Repair Deficiency. Clinical gastroenterology and hepatology : the official clinical practice journal of the American Gastroenterological Association. 2016;14(8):1163-71.

13. Ashktorab H, Ahuja S, Kannan L, Llor X, Ellis NA, Xicola RM, et al. A meta-analysis of MSI frequency and race in colorectal cancer. Oncotarget. 2016;7(23):34546-57.

14. Konstantinopoulos PA, Norquist B, Lacchetti C, Armstrong D, Grisham RN, Goodfellow PJ, et al. Germline and Somatic Tumor Testing in Epithelial Ovarian Cancer: ASCO Guideline. Journal of Clinical Oncology. 2020;38(11):1222-45.

15. Rice TW, Kelsen DP, Blackstone EH, et al. Esophagus and esophagogastric junction. In: Amin MB, Edge SB, Greene FL, et al., editors. AJCC Cancer Staging Manual, 8th ed. New York: Springer, 2017:185-202. p. 185-202.

16. Albayrak A, Garrido-Castro AC, Giannakis M, Umeton R, Manam MD, Stover EH, et al. Clinical pan-cancer assessment of mismatch repair deficiency using tumor-only, targeted next-generation sequencing. JCO Precision Oncology. 2020;4:1084-97.

17. Agy FE, Otmani IE, Mazti A, Lahmidani N, Oussaden A, Abkari ME, et al. Implication of microsatellite instability pathway in outcome of colon cancer in moroccan population. Disease Markers. 2019;2019 (no pagination)(3210710).

18. Rico SD, Hoflmayer D, Buscheck F, Dum D, Luebke AM, Kluth M, et al. Elevated MUC5AC expression is associated with mismatch repair deficiency and proximal tumor location but not with cancer progression in colon cancer. Medical Molecular Morphology. 2020.

19. Erbs E, Rafaelsen SR, Lindebjerg J, Jensen LH, Hansen TF. The impact of mismatch repair status to the preoperative staging of colon cancer: Implications for clinical management. Colorectal Cancer. 2020;9 (2) (no pagination)(CRC20).
20. Eriksen AC, Sorensen FB, Lindebjerg J, Hager H, Depont Christensen R, Kjaer-Frifeldt S, et al. Programmed Death Ligand-1 expression in stage II colon cancer - Experiences from a nationwide populationbased cohort. BMC Cancer. 2019;19 (1) (no pagination)(142).
21. Gkekas I, Novotny J, Fabian P, Nemecek R, Palmqvist R, Strigard K, et al. Deficient mismatch repair as a prognostic marker in stage II colon cancer patients. European Journal of Surgical Oncology. 2019;45(10):1854-61.
22. Hestetun KE, Aasebo K, Rosenlund NB, Muller Y, Dahl O, Myklebust MP. Mismatch repair phenotype determines the implications of tumor grade and CDX2 expression in stage II-III colon cancer. Modern Pathology. 2021;34(1):161-70.
23. Jimenez-Rodriguez RM, Patil S, Keshinro A, Shia J, Vakiani E, Stadler Z, et al. Quantitative assessment of tumor-infiltrating lymphocytes in mismatch repair proficient colon cancer. Oncoimmunology. 2020;9 (1) (no pagination)(1841948).
24. Loughrey MB, McGrath J, Coleman HG, Bankhead P, Maxwell P, McGready C, et al. Identifying mismatch repair-deficient colon cancer: near-perfect concordance between immunohistochemistry and microsatellite instability testing in a large, population-based series. Histopathology. 2020.
25. Pages F, Andre T, Taieb J, Vernerey D, Henriques J, Borg C, et al. Prognostic and predictive value of the Immunoscore in stage III colon cancer patients treated with oxaliplatin in the prospective IDEA France PRODIGE-GERCOR cohort study. Annals of Oncology. 2020;31(7):921-9.
26. Favazza LA, Parseghian CM, Kaya C, Nikiforova MN, Roy S, Wald AI, et al. KRAS amplification in metastatic colon cancer is associated with a history of inflammatory bowel disease and may confer resistance to anti-EGFR therapy. Modern Pathology. 2020;33(9):1832-43.
27. Cao Y, Peng T, Li H, Yang M, Wu L, Zhou Z, et al. Development and validation of MMR prediction model based on simplified clinicopathological features and serum tumour markers. EBioMedicine. 2020;61 (no pagination)(103060).
28. Chikatani K, Chika N, Suzuki O, Sakimoto T, Ishibashi K, Eguchi H, et al. Clinically applicable cases of anti-programmed cell death protein 1 immunotherapy for colorectal cancer patients. Surgery Today. 2020;50(12):1694-8.
29. Chou A, Fraser T, Ahadi M, Fuchs T, Sioson L, Clarkson A, et al. NTRK gene rearrangements are highly enriched in MLH1/PMS2 deficient, BRAF wild-type colorectal carcinomas-a study of 4569 cases. Modern Pathology. 2020;33(5):924-32.
30. Dong L, Jin X, Wang W, Ye Q, Li W, Shi S, et al. Distinct clinical phenotype and genetic testing strategy for Lynch syndrome in China based on a large colorectal cancer cohort. International Journal of Cancer. 2020;146(11):3077-86.
31. Jiang W, Sui QQ, Li WL, Ke CF, Ling YH, Liao LE, et al. Low prevalence of mismatch repair deficiency in Chinese colorectal cancers: A multicenter study. Gastroenterology Report. 2020;8(5):399-403.
32. Jin J, Shi Y, Zhang S, Yang S. PIK3CA mutation and clinicopathological features of colorectal cancer: a systematic review and Meta-Analysis. Acta Oncologica. 2020;59(1):66-74.
33. Koch KE, Goffredo P, Hrabe JE, Gribovskaja-Rupp I, Snow AN, Bellizzi AM, et al. Impact of routine mismatch repair screening on genetic counseling and surgical management in colorectal cancer patients. American Journal of Surgery. 2020.
34. Li D, Hoodfar E, Jiang SF, Udaltsova N, Pham NP, Jodesty Y, et al. Comparison of universal versus age-restricted screening of colorectal tumors for lynch syndrome using mismatch repair immunohistochemistry: A cohort study. Annals of internal medicine. 2019;171(1):19-26.
35. Li C, Liu F, Huang D, Wu Y, Wang Z, Xu Y. The correlation between DNA mismatch repair status and the clinicopathological and molecular features of Chinese sporadic colorectal cancer. Translational Cancer Research. 2020;9(1):137-44.

36. Mendis S, Beck S, Lee B, Lee M, Wong R, Kosmider S, et al. Right versus left sided metastatic colorectal cancer: Teasing out clinicopathologic drivers of disparity in survival. Asia-Pacific Journal of Clinical Oncology. 2019;15(3):136-43.
37. Christakis AG, Papke DJ, Nowak JA, Yurgelun MB, Agoston AT, Lindeman NI, et al. Targeted Cancer Next-Generation Sequencing as a Primary Screening Tool for Microsatellite Instability and Lynch Syndrome in Upper Gastrointestinal Tract Cancers. Cancer epidemiology, biomarkers & prevention : a publication of the American Association for Cancer Research, cosponsored by the American Society of Preventive Oncology. 2019;28(7):1246-51.
38. Lowenthal BM, Nason KS, Pennathur A, Luketich JD, Pai RK, Davison JM, et al. Loss of ARID1A expression is associated with DNA mismatch repair protein deficiency and favorable prognosis in advanced stage surgically resected esophageal adenocarcinoma. Human Pathology. 2019;94:1-10.
39. Svensson MC, Borg D, Zhang C, Hedner C, Nodin B, Uhlen M, et al. Expression of PD-L1 and PD-1 in chemoradiotherapy-Naive esophageal and gastric adenocarcinoma: Relationship with mismatch repair status and survival. Frontiers in Oncology. 2019;9 (MAR) (no pagination)(136).
40. Dislich B, Blaser N, Berger MD, Gloor B, Langer R. Preservation of Epstein-Barr virus status and mismatch repair protein status along the metastatic course of gastric cancer. Histopathology. 2020;76(5):740-7.
41. Huang SC, Ng KF, Yeh TS, Cheng CT, Lin JS, Liu YJ, et al. Subtraction of Epstein-Barr virus and microsatellite instability genotypes from the Lauren histotypes: Combined molecular and histologic subtyping with clinicopathological and prognostic significance validated in a cohort of 1,248 cases. International Journal of Cancer. 2019;145(12):3218-30.
42. Lorenzi M, Amonkar M, Zhang J, Mehta S, Liaw KL. Epidemiology of Microsatellite Instability High (MSI-H) and Deficient Mismatch Repair (dMMR) in Solid Tumors: A Structured Literature Review. Journal of Oncology. 2020;2020 (no pagination)(1807929).
43. Schoop I, Maleki SS, Behrens HM, Kruger S, Haag J, Rocken C. p53 immunostaining cannot be used to predict TP53 mutations in gastric cancer: results from a large Central European cohort. Human Pathology. 2020;105:53-66.
44. Ulase D, Heckl S, Behrens HM, Kruger S, Rocken C. Prognostic significance of tumour budding assessed in gastric carcinoma according to the criteria of the International Tumour Budding Consensus Conference. Histopathology. 2020;76(3):433-46.
45. Zang YS, Dai C, Xu X, Cai X, Wang G, Wei J, et al. Comprehensive analysis of potential immunotherapy genomic biomarkers in 1000 Chinese patients with cancer. Cancer Medicine. 2019;8(10):4699-708.
46. Bang S, Kim H, Jang K, Paik SS, Shin SJ. The loss of nuclear expression of singlestranded DNA binding protein 2 of gastric adenocarcinoma and its prognostic role: Analysis of molecular subtype. PLoS ONE. 2020;15 (8 August) (no pagination)(e0236896).
47. Bosch F, Todorova R, Link H, Westphalen CB, Boeck S, Heinemann V, et al. Molecular subtyping of gastric cancer with respect to the growth pattern of lymph-node metastases. Journal of Cancer Research and Clinical Oncology. 2019;145(11):2689-97.
48. Kim SM, An JY, Byeon SJ, Lee J, Kim KM, Choi MG, et al. Prognostic value of mismatch repair deficiency in patients with advanced gastric cancer, treated by surgery and adjuvant 5-fluorouracil and leucovorin chemoradiotherapy. European Journal of Surgical Oncology. 2019.
49. Ramos MFKP, Pereira MA, Amorim LC, de Mello ES, Faraj SF, Ribeiro U, et al. Gastric cancer molecular classification and adjuvant therapy: Is there a different benefit according to the subtype? Journal of Surgical Oncology. 2020;121(5):804-13.
50. Tsai CY, Lin TA, Huang SC, Hsu JT, Yeh CN, Chen TC, et al. Is Adjuvant Chemotherapy Necessary for Patients with Deficient Mismatch Repair Gastric Cancer?-Autophagy Inhibition Matches the Mismatched. The oncologist. 2020;25(7):e1021-e30.
51. Kawazoe A, Shitara K, Kuboki Y, Bando H, Kojima T, Yoshino T, et al. Clinicopathological features of 22C3 PD-L1 expression with mismatch repair, Epstein-Barr virus status, and cancer genome alterations in metastatic gastric cancer. Gastric Cancer. 2019;22(1):69-76.

52. Mishima S, Kawazoe A, Nakamura Y, Sasaki A, Kotani D, Kuboki Y, et al. Clinicopathological and molecular features of responders to nivolumab for patients with advanced gastric cancer. Journal for immunotherapy of cancer. 2019;7 (1) (no pagination)(24).
53. Ahmad-Nielsen SA, Bruun Nielsen MF, Mortensen MB, Detlefsen S. Frequency of mismatch repair deficiency in pancreatic ductal adenocarcinoma. Pathology Research and Practice. 2020;216 (6) (no pagination)(152985).
54. Taghizadeh H, Mullauer L, Mader RM, Schindl M, Prager GW. Applied precision medicine in metastatic pancreatic ductal adenocarcinoma. Therapeutic Advances in Medical Oncology. 2020;12(no pagination).
55. Acar T, Acar N, Kamer E, Tekindal MA, Cengiz F, Kar H, et al. Do microsatellite instability (MSI) and deficient mismatch repair (dMMR) affect the pathologic complete response (pCR) in patients with rectal cancer who received neoadjuvant treatment? Updates in Surgery. 2020;72(1):73-82.
56. Cho HJ, Baek JH, Baek DW, Kang BW, Lee SJ, Kim HJ, et al. Prognostic significance of clinicopathological and molecular features after neoadjuvant chemoradiotherapy in rectal cancer patients. In Vivo. 2019;33(6):1959-65.
57. Meillan N, Vernerey D, Lefevre JH, Manceau G, Svrcek M, Augustin J, et al. Mismatch Repair System Deficiency Is Associated With Response to Neoadjuvant Chemoradiation in Locally Advanced Rectal Cancer. International Journal of Radiation Oncology Biology Physics. 2019;105(4):824-33.
58. Ostwal V, Pande NS, Engineer R, Saklani A, deSouza A, Ramadwar M, et al. Low prevalence of deficient mismatch repair (dMMR) protein in locally advanced rectal cancers (LARC) and treatment outcomes. Journal of Gastrointestinal Oncology. 2019;10(1):19-29.
59. Aparicio T, Svrcek M, Henriques J, Afchain P, Lievre A, Tougeron D, et al. Panel gene profiling of small bowel adenocarcinoma: Results from the NADEGE prospective cohort. International Journal of Cancer. 2020.
60. Gonzalez I, Goyal B, Xia MD, Pai RK, Ma C. DNA mismatch repair deficiency but not ARID1A loss is associated with prognosis in small intestinal adenocarcinoma. Human Pathology. 2019;85:18-26.
61. Klose J, Lasitschka F, Horsch C, Strowitzki MJ, Bruckner T, Volz C, et al. Prognostic relevance of programmed death-ligand 1 expression and microsatellite status in small bowel adenocarcinoma. Scandinavian Journal of Gastroenterology. 2020;55(3):321-9.
62. Noh BJ, Hong SM, Jun SY, Eom DW. Prognostic implications of immune classification in a multicentre cohort of patients with small intestinal adenocarcinoma. Pathology. 2020;52(2):228-35.
63. Suerink M, Kilinc G, Terlouw D, Hristova H, Sensuk L, Van Egmond D, et al. Prevalence of mismatch repair deficiency and Lynch syndrome in a cohort of unselected small bowel adenocarcinomas. Journal of Clinical Pathology. 2020;(no pagination)(207040).
64. Fraune C, Simon R, Hube-Magg C, Makrypidi-Fraune G, Kahler C, Kluth M, et al. MMR deficiency in urothelial carcinoma of the bladder presents with temporal and spatial homogeneity throughout the tumor mass. Urologic Oncology: Seminars and Original Investigations. 2020;38(5):488-95.
65. Gayhart MG, Johnson N, Paul A, Quillin JM, Hampton LJ, Idowu MO, et al. Universal Mismatch Repair Protein Screening in Upper Tract Urothelial Carcinoma. American Journal of Clinical Pathology. 2020;154(6):792-801.
66. Hodgson A, Vesprini D, Liu SK, Xu B, Downes MR. Correlation of mismatch repair protein deficiency, PD-L1 and CD8 expression in high-grade urothelial carcinoma of the bladder. Journal of Clinical Pathology. 2020;73(8):519-22.
67. Marchetti A, Di Lorito A, Felicioni L, Buttitta F. An innovative diagnostic strategy for the detection of rare molecular targets to select cancer patients for tumor-agnostic treatments. Oncotarget. 2020;10(68):6957-68.
68. Schneider B, Glass A, Jagdmann S, Huhns M, Claus J, Zettl H, et al. Loss of Mismatch-repair Protein Expression and Microsatellite Instability in Upper Tract Urothelial Carcinoma and Clinicopathologic Implications. Clinical Genitourinary Cancer. 2020;18(5):e563-e72.

69. Stoehr R, Wendler O, Giedl J, Gaisa NT, Richter G, Campean V, et al. No Evidence of Microsatellite Instability and Loss of Mismatch-Repair-Protein Expression in Squamous Cell Carcinoma of the Penis. Pathobiology. 2019;86(2-3):145-51.
70. Albero-Gonzalez R, Hernandez-Llodra S, Juanpere N, Lorenzo M, Lloret A, Segales L, et al. Immunohistochemical expression of mismatch repair proteins (MSH2, MSH6, MLH1, and PMS2) in prostate cancer: correlation with grade groups (WHO 2016) and ERG and PTEN status. Virchows Archiv. 2019;475(2):223-31.
71. Lindh C, Kis L, Delahunt B, Samaratunga H, Yaxley J, Wiklund NP, et al. PD-L1 expression and deficient mismatch repair in ductal adenocarcinoma of the prostate. Apmis. 2019;127(8):554-60.
72. Sharma M, Yang Z, Miyamoto H. Loss of DNA mismatch repair proteins in prostate cancer. Medicine. 2020;99(19):e20124.
73. Fraune C, Simon R, Hoflmayer D, Moller K, Dum D, Buscheck F, et al. High homogeneity of mismatch repair deficiency in advanced prostate cancer. Virchows Archiv. 2020;476(5):745-52.
74. Sadigh S, Farahani SJ, Shah A, Vaughn D, Lal P. Differences in PD-L1-Expressing Macrophages and Immune Microenvironment in Testicular Germ Cell Tumors. American Journal of Clinical Pathology. 2020;153(3):387-95.
75. Cheng AS, Leung SCY, Gao D, Burugu S, Anurag M, Ellis MJ, et al. Mismatch repair protein loss in breast cancer: clinicopathological associations in a large British Columbia cohort. Breast Cancer Research and Treatment. 2020;179(1):3-10.
76. Carr C, Son J, Yao M, Priyadarshini A, Marquard J, Vargas R, et al. Clinicopathologic characteristics and outcomes of endometrial Cancer patients with mismatch repair deficiency in the era of universal Lynch syndrome screening. Gynecologic Oncology. 2020;159(3):712-20.
77. He D, Wang H, Dong Y, Zhang Y, Zhao J, Lv C, et al. POLE mutation combined with microcystic, elongated and fragmented (MELF) pattern invasion in endometrial carcinomas might be associated with poor survival in Chinese women. Gynecologic Oncology. 2020;159(1):36-42.
78. Kolehmainen A, Pasanen A, Tuomi T, Koivisto-Korander R, Butzow R, Loukovaara M. Clinical factors as prognostic variables among molecular subgroups of endometrial cancer. PLoS ONE. 2020;15 (11 November) (no pagination)(e0242733).
79. Loukovaara M, Pasanen A, Butzow R. Mismatch repair protein and MLH1 methylation status as predictors of response to adjuvant therapy in endometrial cancer. Cancer Medicine. 2021.
80. Ryan NAJ, McMahon R, Tobi S, Snowsill T, Esquibel S, Wallace AJ, et al. The proportion of endometrial tumours associated with Lynch syndrome (PETALS): A prospective cross-sectional study. PLoS Medicine. 2020;17 (9) (no pagination)(1003263).
81. Ryan NAJ, Glaire MA, Blake D, Cabrera-Dandy M, Evans DG, Crosbie EJ. The proportion of endometrial cancers associated with Lynch syndrome: a systematic review of the literature and meta-analysis. Genetics in Medicine. 2019;21(10):2167-80.
82. Talhouk A, Derocher H, Schmidt P, Leung S, Milne K, Blake Gilks C, et al. Molecular subtype not immune response drives outcomes in endometrial carcinoma. Clinical Cancer Research. 2019;25(8):2537-48.
83. Kahn RM, Gordhandas S, Maddy BP, Baltich Nelson B, Askin G, Christos PJ, et al. Universal endometrial cancer tumor typing: How much has immunohistochemistry, microsatellite instability, and MLH1 methylation improved the diagnosis of Lynch syndrome across the population? Cancer. 2019;125(18):3172-83.
84. Kim SR, Pina A, Albert A, McAlpine JN, Wolber R, Gilks B, et al. Mismatch repair deficiency and prognostic significance in patients with low-risk endometrioid endometrial cancers. International Journal of Gynecological Cancer. 2020;30(6):783-8.
85. Leon-Castillo A, De Boer SM, Powell ME, Mileshkin LR, Mackay HJ, Leary A, et al. Molecular classification of the PORTEC-3 trial for high-risk endometrial cancer: Impact on prognosis and benefit from adjuvant therapy. Journal of Clinical Oncology. 2020;38(29):3388-97.

86. Stasenko M, Feit N, Lee SSK, Shepherd C, Soslow RA, Cadoo KA, et al. Clinical patterns and genomic profiling of recurrent ultra-low risk' endometrial cancer. International Journal of Gynecological Cancer. 2020;30(6):717-23.
87. Bennett JA, Pesci A, Morales-Oyarvide V, Da Silva A, Nardi V, Oliva E. Incidence of Mismatch Repair Protein Deficiency and Associated Clinicopathologic Features in a Cohort of 104 Ovarian Endometrioid Carcinomas. American Journal of Surgical Pathology. 2019;43(2):235-43.
88. Fraune C, Rosebrock J, Simon R, Hube-Magg C, Makrypidi-Fraune G, Kluth M, et al. High homogeneity of MMR deficiency in ovarian cancer. Gynecologic Oncology. 2020;156(3):669-75.
89. Hodan R, Kingham K, Cotter K, Folkins AK, Kurian AW, Ford JM, et al. Prevalence of Lynch syndrome in women with mismatch repair-deficient ovarian cancer. Cancer Medicine. 2020.
90. Kim SR, Tone A, Kim RH, Cesari M, Clarke BA, Eiriksson L, et al. Performance characteristics of screening strategies to identify Lynch syndrome in women with ovarian cancer. Cancer. 2020;126(22):4886-94.
91. Schmoeckel E, Hofmann S, Fromberger D, Rottmann M, Luthardt B, Burges A, et al. Comprehensive analysis of PD-L1 expression, HER2 amplification, ALK/EML4 fusion, and mismatch repair deficiency as putative predictive and prognostic factors in ovarian carcinoma. Virchows Archiv. 2019;474(5):599-608.
92. Yamashita H, Nakayama K, Ishikawa M, Ishibashi T, Nakamura K, Sawada K, et al. Relationship between microsatellite instability, immune cells infiltration, and expression of immune checkpoint molecules in ovarian carcinoma: Immunotherapeutic strategies for the future. International journal of molecular sciences. 2019;20 (20) (no pagination)(5129).
93. Zhu J, Ke G, Bi R, Wu X. Clinicopathological and survival characteristic of mismatch repair status in ovarian clear cell carcinoma. Journal of Surgical Oncology. 2020;122(3):538-46.
94. Leskela S, Romero I, Cristobal E, Perez-Mies B, Rosa-Rosa JM, Gutierrez-Pecharroman A, et al. Mismatch Repair Deficiency in Ovarian Carcinoma: Frequency, Causes, and Consequences. American Journal of Surgical Pathology. 2020;44(5):649-56.
95. Xue Y, Balci S, Aydin Mericoz C, Taskin OC, Jiang H, Pehlivanoglu B, et al. Frequency and clinicopathologic associations of DNA mismatch repair protein deficiency in ampullary carcinoma: Routine testing is indicated. Cancer. 2020;126(21):4788-99.
96. Ju JY, Dibbern ME, Mahadevan MS, Fan J, Kunk PR, Stelow EB. Mismatch Repair Protein Deficiency/Microsatellite Instability Is Rare in Cholangiocarcinomas and Associated with Distinctive Morphologies. American Journal of Clinical Pathology. 2020;153(5):598-604.
97. Spizzo G, Puccini A, Xiu J, Goldberg RM, Grothey A, Shields AF, et al. Molecular profile of BRCA-mutated biliary tract cancers. ESMO Open. 2020;5(3).
98. Guazzo E, Cooper C, Wilkinson L, Feng S, King B, Simpson F, et al. Therapeutic implications of immune-profiling and EGFR expression in salivary gland carcinoma. Head and Neck. 2020.
99. Vasan K, Satgunaseelan L, Anand S, Asher R, Selinger C, Low THH, et al. Tumour mismatch repair protein loss is associated with advanced stage in oral cavity squamous cell carcinoma. Pathology. 2019;51(7):688-95.
100. Zhao L, Liao X, Hong G, Zhuang Y, Fu K, Chen P, et al. Mismatch repair status and high expression of PD-LI in nasopharyngeal carcinoma. Cancer Management and Research. 2019;11:1631-40.
101. Ren Y, Lv Q, Yue W, Liu B, Zou Z. The programmed cell death protein-1/programmed cell death ligand 1 expression, CD3+ T cell infiltration, NY-ESO-1 expression, and microsatellite instability phenotype in primary cutaneous melanoma and mucosal melanoma and their clinical significance and prognostic value: a study of 89 consecutive cases. Melanoma Research. 2020;30(1):85-101.
102. Vasan K, Anand S, Satgunaseelan L, Asher R, Low H, Palme CE, et al. Mismatch repair protein loss in cutaneous head and neck squamous cell carcinoma. Journal of Surgical Oncology. 2020;122(8):1755-60.

103. McCord M, Steffens A, Javier R, Kam KL, McCortney K, Horbinski C. The efficacy of DNA mismatch repair enzyme immunohistochemistry as a screening test for hypermutated gliomas. Acta Neuropathologica Communications. 2020;8(1):15.
104. Almuhaisen G, Alhalaseh Y, Mansour R, Abu-Shanab A, Al-Ghnimat S, Al-Hussaini M. Frequency of mismatch repair protein deficiency and PD-L1 in high-grade gliomas in adolescents and young adults (AYA). Brain Tumor Pathology. 2021;38(1):14-22.
105. Caccese M, Ius T, Simonelli M, Fassan M, Cesselli D, Dipasquale A, et al. Mismatch-repair protein expression in high-grade gliomas: A large retrospective multicenter study. International journal of molecular sciences. 2020;21(18):1-12.
106. Indraccolo S, Lombardi G, Fassan M, Pasqualini L, Giunco S, Marcato R, et al. Genetic, epigenetic, and immunologic profiling of MMR-deficient relapsed glioblastoma. Clinical Cancer Research. 2019;25(6):1828-37.
107. Tepeoglu M, Borcek P, Ozen O, Altinors N. Microsatellite Instability in Glioblastoma: Is It Really Relevant in Tumor Prognosis? Turkish Neurosurgery. 2019;29(5):778-84.
108. Yan F, Lin Y, Zhou Q, Chang H, Li Y. Pathological prognostic factors of pseudomyxoma peritonei: comprehensive clinicopathological analysis of 155 cases. Human Pathology. 2020;97:9-18.
109. Nikanjam M, Arguello D, Gatalica Z, Swensen J, Barkauskas DA, Kurzrock R. Relationship between protein biomarkers of chemotherapy response and microsatellite status, tumor mutational burden and PD-L1 expression in cancer patients. International Journal of Cancer. 2020;146(11):3087-97.
110. Yoshino T, Pentheroudakis G, Mishima S, Overman MJ, Yeh KH, Baba E, et al. JSCO-ESMO-ASCO-JSMO-TOS: international expert consensus recommendations for tumour-agnostic treatments in patients with solid tumours with microsatellite instability or NTRK fusions. Annals of Oncology. 2020;31(7):861-72.
111. Tokunaga R, Xiu J, Johnston C, Goldberg RM, Philip PA, Seeber A, et al. Molecular profiling of appendiceal adenocarcinoma and comparison with right-sided and left-sided colorectal cancer. Clinical Cancer Research. 2019;25(10):3096-103.
112. Marquet B, Marchal Bressenot A, Fichel C, Bouland N, Barbe C, Bouche O, et al. Expression of the Serrated Markers Annexin A10 or Gremlin1 in Colonic Adenocarcinomas: Morphology and Prognostic Values. Pathology and Oncology Research. 2020;26(4):2509-21.
113. Chouhan H, Sammour T, M LT, J WM. Prognostic significance of BRAF mutation alone and in combination with microsatellite instability in stage III colon cancer. Asia-Pacific Journal of Clinical Oncology. 2019;15(1):69-74.
114. Mlecnik B, Bifulco C, Bindea G, Marliot F, Lugli A, Lee JJ, et al. Multicenter international society for immunotherapy of cancer study of the consensus immunoscore for the prediction of survival and response to chemotherapy in stage III colon cancer. Journal of Clinical Oncology. 2020;38(31):3638-51.
115. Shaib WL, Zakka KM, Jiang R, Yan M, Alese OB, Akce M, et al. Survival outcome of adjuvant chemotherapy in deficient mismatch repair stage III colon cancer. Cancer. 2020;126(18):4136-47.
116. Ueno H, Ishiguro M, Nakatani E, Ishikawa T, Uetake H, Matsui S, et al. Optimal Criteria for G3 (Poorly Differentiated) Stage II Colon Cancer: Prospective Validation in a Randomized Controlled Study (SACURA Trial). American Journal of Surgical Pathology. 2020;44(12):1685-98.
117. Achilli P, Crippa J, Grass F, Mathis KL, D'Angelo ALD, Abd El Aziz MA, et al. Survival impact of adjuvant chemotherapy in patients with stage IIA colon cancer: Analysis of the National Cancer Database. International Journal of Cancer. 2021;148(1):161-9.
118. Blaker H, Alwers E, Arnold A, Herpel E, Tagscherer KE, Roth W, et al. The Association Between Mutations in BRAF and Colorectal Cancer-Specific Survival Depends on Microsatellite Status and Tumor Stage. Clinical Gastroenterology and Hepatology. 2019;17(3):455-62.e6.
119. Chen MH, Chang SC, Lin PC, Yang SH, Lin CC, Lan YT, et al. Combined Microsatellite Instability and Elevated Microsatellite Alterations at Selected Tetranucleotide Repeats (EMAST) Might Be a More Promising Immune Biomarker in Colorectal Cancer. The oncologist. 2019;24(12):1534-42.

120. Zhang M, Hu W, Hu K, Lin Y, Feng Z, Yun JP, et al. Association of KRAS mutation with tumor deposit status and overall survival of colorectal cancer. Cancer Causes and Control. 2020;31(7):683-9.
121. Cui M, Li P, Mao Y, Zhang L, Xia P, Liu E, et al. Implication of microsatellite instability in Chinese cohort of human cancers. Cancer Management and Research. 2020;12:10287-95.
122. Han SA, Kim JH, Choi JH, Lee DH, Jung K, Kim SE, et al. The Clinical Significance of Microsatellite Instability in Patients with Right-sided Colorectal Cancer. Korean Journal of Gastroenterology/Taehan Sohwagi Hakhoe Chi. 2019;73(3):159-66.
123. Hatakeyama K, Nagashima T, Ohshima K, Ohnami S, Shimoda Y, Serizawa M, et al. Mutational burden and signatures in 4000 Japanese cancers provide insights into tumorigenesis and response to therapy. Cancer Science. 2019;110(8):2620-8.
124. Middha S, Yaeger R, Shia J, Stadler ZK, King S, Guercio S, et al. Majority of B2M-mutant and - deficient colorectal carcinomas achieve clinical benefit from immune checkpoint inhibitor therapy and are microsatellite instability-high. JCO Precision Oncology. 2019;3(no pagination).
125. Phipps AI, Alwers E, Harrison T, Banbury B, Brenner H, Campbell PT, et al. Association Between Molecular Subtypes of Colorectal Tumors and Patient Survival, Based on Pooled Analysis of 7 International Studies. Gastroenterology. 2020;158(8):2158-68.e4.
126. Dienstmann R, Villacampa G, Sveen A, Mason MJ, Niedzwiecki D, Nesbakken A, et al. Relative contribution of clinicopathological variables, genomic markers, transcriptomic subtyping and microenvironment features for outcome prediction in stage II/III colorectal cancer. Annals of Oncology. 2019;30(10):1622-9.
127. Oh HJ, Bae JM, Wen X, Jung S, Kim Y, Kim KJ, et al. p53 expression status is associated with cancer-specific survival in stage III and high-risk stage II colorectal cancer patients treated with oxaliplatin-based adjuvant chemotherapy. British Journal of Cancer. 2019;120(8):797-805.
128. Cavallaro P, Bordeianou L, Stafford C, Clark J, Berger D, Cusack J, et al. Impact of Single-organ Metastasis to the Liver or Lung and Genetic Mutation Status on Prognosis in Stage IV Colorectal Cancer. Clinical Colorectal Cancer. 2020;19(1):e8-e17.
129. Innocenti F, Ou FS, Qu X, Zemla TJ, Niedzwiecki D, Tam R, et al. Mutational analysis of patients with colorectal cancer in CALGB/SWOG 80405 identifies new roles of microsatellite instability and tumor mutational burden for patient outcome. Journal of Clinical Oncology. 2019;37(14):1217-27.
130. Nunes L, Aasebo K, Mathot L, Ljungstrom V, Edqvist PH, Sundstrom M, et al. Molecular characterization of a large unselected cohort of metastatic colorectal cancers in relation to primary tumor location, rare metastatic sites and prognosis. Acta Oncologica. 2020;59(4):417-26.
131. Sastre J, Orden VDL, Martinez A, Bando I, Balbin M, Bellosillo B, et al. Association Between Baseline Circulating Tumor Cells, Molecular Tumor Profiling, and Clinical Characteristics in a Large Cohort of Chemo-naive Metastatic Colorectal Cancer Patients Prospectively Collected. Clinical Colorectal Cancer. 2020;19(3):e110-e6.
132. Stahler A, Stintzing S, von Einem JC, Westphalen CB, Heinrich K, Kramer N, et al. Single-nucleotide variants, tumour mutational burden and microsatellite instability in patients with metastatic colorectal cancer: Next-generation sequencing results of the FIRE-3 trial. European Journal of Cancer. 2020;137:250-9.
133. Fan Y, Ying H, Wu X, Chen H, Hu Y, Zhang H, et al. The mutational pattern of homologous recombination (HR)-associated genes and its relevance to the immunotherapeutic response in gastric cancer. Cancer Biology and Medicine. 2020;17(4):1002-13.
134. Kohlruss M, Grosser B, Krenauer M, Slotta-Huspenina J, Jesinghaus M, Blank S, et al. Prognostic implication of molecular subtypes and response to neoadjuvant chemotherapy in 760 gastric carcinomas: role of Epstein-Barr virus infection and high- and low-microsatellite instability. Journal of Pathology: Clinical Research. 2019;5(4):227-39.
135. Polom K, Das K, Marrelli D, Roviello G, Pascale V, Voglino C, et al. KRAS Mutation in Gastric Cancer and Prognostication Associated with Microsatellite Instability Status. Pathology and Oncology Research. 2019;25(1):333-40.

136. Wu CH, Tseng CH, Huang KH, Fang WL, Chen MH, Li A, et al. The clinical significance of ARID1A mutations in gastric cancer patients. Formosan Journal of Surgery. 2020;53(3):93-100.
137. Zhou J, Zhao Z, Zhang Y, Bao C, Cui L, Cai S, et al. Pathogenic Germline Mutations in Chinese Patients with Gastric Cancer Identified by Next-Generation Sequencing. Oncology (Switzerland). 2020;98(8):583-8.
138. Cai Z, Rui W, Li S, Fingerhut A, Sun J, Ma J, et al. Microsatellite Status Affects Tumor Response and Survival in Patients Undergoing Neoadjuvant Chemotherapy for Clinical Stage III Gastric Cancer. Frontiers in Oncology. 2020;10 (no pagination)(614785).
139. Dai D, Zhao X, Li X, Shu Y, Shen B, Chen X, et al. Association Between the Microsatellite Instability Status and the Efficacy of Postoperative Adjuvant Chemoradiotherapy in Patients With Gastric Cancer. Frontiers in Oncology. 2020;9 (no pagination)(1452).
140. Haag GM, Czink E, Ahadova A, Schmidt T, Sisic L, Blank S, et al. Prognostic significance of microsatellite-instability in gastric and gastroesophageal junction cancer patients undergoing neoadjuvant chemotherapy. International Journal of Cancer. 2019;144(7):1697-703.
141. Huang KH, Chen MH, Fang WL, Lin CH, Chao Y, Lo SS, et al. The clinicopathological characteristics and genetic alterations of signet-ring cell carcinoma in gastric cancer. Cancers. 2020;12(8):1-17.
142. Jiang D, Shu C, Zhang W, Sun L, Zhang M, He Y, et al. Low level of microsatellite instability correlates with short disease-free survival of gastric cancer patients undergoing neoadjuvant chemotherapy. Virchows Archiv. 2020.
143. Kim DG, An JY, Kim H, Shin SJ, Choi S, Seo WJ, et al. Clinical implications of microsatellite instability in early gastric cancer. Journal of Gastric Cancer. 2019;19(4):427-37.
144. Kim JW, Cho SY, Chae J, Kim JW, Kim TY, Lee KW, et al. Adjuvant chemotherapy in microsatellite instability-high gastric cancer. Cancer Research and Treatment. 2020;52(4):1178-87.
145. Koh J, Nam SK, Roh H, Kim J, Lee BC, Kim JW, et al. Somatic mutational profiles of stage II and III gastric cancer according to tumor microenvironment immune type. Genes Chromosomes and Cancer. 2019;58(1):12-22.
146. Molinari C, Tedaldi G, Rebuzzi F, Morgagni P, Capelli L, Ravaioli S, et al. Early Gastric Cancer: identification of molecular markers able to distinguish submucosa-penetrating lesions with different prognosis. Gastric Cancer. 2020.
147. Pietrantonio F, Miceli R, Raimondi A, Kim YW, Kang WK, Langley RE, et al. Individual patient data meta-analysis of the value of microsatellite instability as a biomarker in gastric cancer. Journal of Clinical Oncology. 2019;37(35):3392-400.
148. Yun S, Koh J, Nam SK, Kwak Y, Ahn SH, Do Park J, et al. Immunoscore is a strong predictor of survival in the prognosis of stage II/III gastric cancer patients following 5-FU-based adjuvant chemotherapy. Cancer Immunology, Immunotherapy. 2020.
149. Kim J, Kim B, Kang SY, Heo YJ, Park SH, Kim ST, et al. Tumor Mutational Burden Determined by Panel Sequencing Predicts Survival After Immunotherapy in Patients With Advanced Gastric Cancer. Frontiers in Oncology. 2020;10 (no pagination)(314).
150. Kwon M, Hong JY, Kim ST, Kim KM, Lee J. Association of serine/threonine kinase 11 mutations and response to programmed cell death 1 inhibitors in metastatic gastric cancer. Pathology Research and Practice. 2020;216 (6) (no pagination)(152947).
151. Wang F, Wei XL, Wang FH, Xu N, Shen L, Dai GH, et al. Safety, efficacy and tumor mutational burden as a biomarker of overall survival benefit in chemo-refractory gastric cancer treated with toripalimab, a PD-1 antibody in phase Ib/II clinical trial NCT02915432. Annals of Oncology. 2019;30(9):1479-86.
152. Ang C, Klempner SJ, Ali SM, Madison R, Ross JS, Severson EA, et al. Prevalence of established and emerging biomarkers of immune checkpoint inhibitor response in advanced hepatocellular carcinoma. Oncotarget. 2019;10(40):4018-25.

153. Kawaoka T, Ando Y, Yamauchi M, Suehiro Y, Yamaoka K, Kosaka Y, et al. Incidence of microsatellite instability-high hepatocellular carcinoma among Japanese patients and response to pembrolizumab. Hepatology Research. 2020;50(7):885-8.
154. Luchini C, Brosens LAA, Wood LD, Chatterjee D, Shin JI, Sciammarella C, et al. Comprehensive characterisation of pancreatic ductal adenocarcinoma with microsatellite instability: Histology, molecular pathology and clinical implications. Gut. 2021;70(1):148-56.
155. Fraune C, Burandt E, Simon R, Hube-Magg C, Makrypidi-Fraune G, Kluth M, et al. MMR Deficiency is Homogeneous in Pancreatic Carcinoma and Associated with High Density of Cd8-Positive Lymphocytes. Annals of Surgical Oncology. 2020;27(10):3997-4006.
156. Hasan S, Renz P, Wegner RE, Finley G, Raj M, Monga D, et al. Microsatellite Instability (MSI) as an Independent Predictor of Pathologic Complete Response (PCR) in Locally Advanced Rectal Cancer: A National Cancer Database (NCDB) Analysis. Annals of Surgery. 2020;271(4):716-23.
157. Lee JH, Kang BH, Song C, Kang SB, Lee HS, Lee KW, et al. Microsatellite instability correlated inflammatory markers and their prognostic value in the rectal cancer following neoadjuvant chemoradiotherapy: A hypothesis-generating study. In Vivo. 2020;34(4):2119-26.
158. Giuffrida P, Arpa G, Grillo F, Klersy C, Sampietro G, Ardizzone S, et al. PD-L1 in small bowel adenocarcinoma is associated with etiology and tumor-infiltrating lymphocytes, in addition to microsatellite instability. Modern Pathology. 2020;33(7):1398-409.
159. Wirta EV, Szeto S, Hanninen U, Ahtiainen M, Bohm J, Mecklin JP, et al. Prognostic value of immune environment analysis in small bowel adenocarcinomas with verified mutational landscape and predisposing conditions. Cancers. 2020;12(8):1-19.
160. Vanoli A, Grillo F, Guerini C, Neri G, Arpa G, Klersy C, et al. Prognostic Role of Mismatch Repair Status, Histotype and High-Risk Pathologic Features in Stage II Small Bowel Adenocarcinomas. Annals of Surgical Oncology. 2021;28(2):1167-77.
161. Jun SY, Park ES, Lee JJ, Chang HK, Jung ES, Oh YH, et al. Prognostic significance of stromal and intraepithelial tumor-infiltrating lymphocytes in small intestinal adenocarcinoma. American Journal of Clinical Pathology. 2020;153(1):105-18.
162. Audenet F, Isharwal S, Cha EK, Donoghue MTA, Drill EN, Ostrovnaya I, et al. Clonal relatedness and mutational differences between upper tract and bladder urothelial carcinoma. Clinical Cancer Research. 2019;25(3):967-76.
163. Necchi A, Madison R, Raggi D, Jacob JM, Bratslavsky G, Shapiro O, et al. Comprehensive Assessment of Immuno-oncology Biomarkers in Adenocarcinoma, Urothelial Carcinoma, and Squamous-cell Carcinoma of the Bladder. European urology. 2020;77(4):548-56.
164. Meng H, Jiang X, Cui J, Yin G, Shi B, Liu Q, et al. Genomic Analysis Reveals Novel Specific Metastatic Mutations in Chinese Clear Cell Renal Cell Carcinoma. BioMed Research International. 2020;2020 (no pagination)(2495157).
165. Carlo MI, Khan N, Zehir A, Patil S, Ged Y, Redzematovic A, et al. Comprehensive genomic analysis of metastatic non-clear-cell renal cell carcinoma to identify therapeutic targets. JCO Precision Oncology. 2019;3(no pagination).
166. Abida W, Cheng ML, Armenia J, Middha S, Autio KA, Vargas HA, et al. Analysis of the Prevalence of Microsatellite Instability in Prostate Cancer and Response to Immune Checkpoint Blockade. JAMA oncology. 2019;5(4):471-8.
167. van Dessel LF, van Riet J, Smits M, Zhu Y, Hamberg P, van der Heijden MS, et al. The genomic landscape of metastatic castration-resistant prostate cancers reveals multiple distinct genotypes with potential clinical impact. Nature Communications. 2019;10 (1) (no pagination)(5251).
168. Necchi A, Bratslavsky G, Corona RJ, Chung JH, Millis SZ, Elvin JA, et al. Genomic Characterization of Testicular Germ Cell Tumors Relapsing After Chemotherapy. European Urology Focus. 2020;6(1):122-30.
169. Sivapiragasam A, Ashok Kumar P, Sokol ES, Albacker LA, Killian JK, Ramkissoon SH, et al. Predictive Biomarkers for Immune Checkpoint Inhibitors in Metastatic Breast Cancer. Cancer Med. 2021;10(1):53-61.

170. Cimic A, Vranic S, Arguello D, Contreras E, Gatalica Z, Swensen J. Molecular Profiling Reveals Limited Targetable Biomarkers in Neuroendocrine Carcinoma of the Cervix. Applied Immunohistochemistry and Molecular Morphology. 2020.
171. Jones NL, Xiu J, Rocconi RP, Herzog TJ, Winer IS. Immune checkpoint expression, microsatellite instability, and mutational burden: Identifying immune biomarker phenotypes in uterine cancer. Gynecologic Oncology. 2020;156(2):393-9.
172. Prendergast EN, Holman LL, Liu AY, Lai TS, Campos MP, Fahey JN, et al. Comprehensive genomic profiling of recurrent endometrial cancer: Implications for selection of systemic therapy. Gynecologic Oncology. 2019;154(3):461-6.
173. Liu YL, Selenica P, Zhou Q, Iasonos A, Callahan M, Feit NZ, et al. BRCA mutations, homologous DNA repair deficiency, tumor mutational burden, and response to immune checkpoint inhibition in recurrent ovarian cancer. JCO Precision Oncology. 2020;4:665-79.
174. Hollis RL, Thomson JP, Stanley B, Churchman M, Meynert AM, Rye T, et al. Molecular stratification of endometrioid ovarian carcinoma predicts clinical outcome. Nature Communications. 2020;11 (1) (no pagination)(4995).
175. Goeppert B, Roessler S, Renner M, Loeffler M, Singer S, Rausch M, et al. Low frequency of mismatch repair deficiency in gallbladder cancer. Diagnostic Pathology. 2019;14(1):36.
176. Goeppert B, Roessler S, Renner M, Singer S, Mehrabi A, Vogel MN, et al. Mismatch repair deficiency is a rare but putative therapeutically relevant finding in non-liver fluke associated cholangiocarcinoma. British Journal of Cancer. 2019;120(1):109-14.
177. Xu SF, Guo Y, Zhang X, Zhu XD, Fan N, Zhang ZL, et al. Somatic Mutation Profiling of Intrahepatic Cholangiocarcinoma: Comparison between Primary and Metastasis Tumor Tissues. Journal of Oncology. 2020;2020 (no pagination)(5675020).
178. Feng F, Wu X, Shi X, Gao Q, Wu Y, Yu Y, et al. Comprehensive analysis of genomic alterations of Chinese hilar cholangiocarcinoma patients. International Journal of Clinical Oncology. 2021.
179. Ahn S, Lee JC, Shin DW, Kim J, Hwang JH. High PD-L1 expression is associated with therapeutic response to pembrolizumab in patients with advanced biliary tract cancer. Scientific Reports. 2020;10(1):12348.
180. Jeon HM, Lee JS, Yun KH, Park KH, Jeon MK, Lee YH, et al. Comprehensive Immuno-Molecular Profiles for Liposarcoma: Roles of Programmed Death Ligand 1, Microsatellite Instability, and PIK3CA. Oncology (Switzerland). 2020;98(11):817-26.
181. Darabi S, Braxton DR, Eisenberg BL, Demeure MJ. Molecular genomic profiling of adrenocortical cancers in clinical practice. Surgery (United States). 2021;169(1):138-44.
182. Marabelle A, Fakih M, Lopez J, Shah M, Shapira-Frommer R, Nakagawa K, et al. Association of tumour mutational burden with outcomes in patients with advanced solid tumours treated with pembrolizumab: prospective biomarker analysis of the multicohort, open-label, phase 2 KEYNOTE-158 study. The Lancet Oncology. 2020;21(10):1353-65.
183. Zhang N, Shi J, Shi X, Chen W, Liu J. Mutational characterization and potential prognostic biomarkers of Chinese patients with esophageal squamous cell carcinoma. OncoTargets and Therapy. 2020;13:12797-809.
184. Huang RSP, Haberberger J, Severson E, Duncan DL, Hemmerich A, Edgerly C, et al. A pan-cancer analysis of PD-L1 immunohistochemistry and gene amplification, tumor mutation burden and microsatellite instability in 48,782 cases. Modern pathology : an official journal of the United States and Canadian Academy of Pathology, Inc. 2021;34(2):252-63.
185. Nassar AH, Umeton R, Kim J, Lundgren K, Harshman L, Van Allen EM, et al. Mutational analysis of 472 urothelial carcinoma across grades and anatomic sites. Clinical Cancer Research. 2019;25(8):2458-70.
186. Zhang C, Shen L, Qi F, Wang J, Luo J. Multi-omics analysis of tumor mutation burden combined with immune infiltrates in bladder urothelial carcinoma. Journal of Cellular Physiology. 2020;235(4):3849-63.

187. Chung JH, Dewal N, Sokol E, Mathew P, Whitehead R, Millis SZ, et al. Prospective comprehensive genomic profiling of primary and metastatic prostate tumors. JCO Precision Oncology. 2019;3(no pagination).
188. Barroso-Sousa R, Jain E, Cohen O, Kim D, Buendia-Buendia J, Winer E, et al. Prevalence and mutational determinants of high tumor mutation burden in breast cancer. Annals of Oncology. 2020;31(3):387-94.
189. Heeke AL, Xiu J, Elliott A, Korn WM, Lynce F, Pohlmann PR, et al. Actionable co-alterations in breast tumors with pathogenic mutations in the homologous recombination DNA damage repair pathway. Breast Cancer Research and Treatment. 2020;184(2):265-75.
190. Kobayashi H, Serizawa M, Naito T, Konno H, Kojima H, Mizuno T, et al. Characterization of tumour mutation burden in patients with non-small cell lung cancer and interstitial lung disease. Respirology. 2020;25(8):850-4.
191. Schatz S, Falk M, Jori B, Ramdani HO, Schmidt S, Willing EM, et al. Integration of tumor mutation burden and pd-l1 testing in routine laboratory diagnostics in non-small cell lung cancer. Cancers. 2020;12(6):1-14.
192. Sakai K, Tsuboi M, Kenmotsu H, Yamanaka T, Takahashi T, Goto K, et al. Tumor mutation burden as a biomarker for lung cancer patients treated with pemetrexed and cisplatin (the JIPANG-TR). Cancer Science. 2021;112(1):388-96.
193. Ready N, Hellmann MD, Awad MM, Otterson GA, Gutierrez M, Gainor JF, et al. First-line nivolumab plus ipilimumab in advanced non-small-cell lung cancer (CheckMate 568): Outcomes by programmed death ligand 1 and tumor mutational burden as biomarkers. Journal of Clinical Oncology. 2019;37(12):992-1000.
194. Ren F, Zhao T, Liu B, Pan L. Neutrophil-lymphocyte ratio (NLR) predicted prognosis for advanced non-small-cell lung cancer (NSCLC) patients who received immune checkpoint blockade (ICB). OncoTargets and Therapy. 2019;12:4235-44.
195. Stein MK, Pandey M, Xiu J, Tae H, Swensen J, Mittal S, et al. Tumor mutational burden is site specific in non-small-cell lung cancer and is highest in lung adenocarcinoma brain metastases. JCO Precision Oncology. 2019;3(no pagination).
196. Willis C, Fiander M, Tran D, Korytowsky B, Thomas JM, Calderon F, et al. Tumor mutational burden in lung cancer: A systematic literature review. Oncotarget. 2019;10(61):6604-22.
197. Jiang G, Zhang W, Wang T, Ding S, Shi X, Zhang S, et al. Characteristics of genomic alterations in Chinese cholangiocarcinoma patients. Japanese journal of clinical oncology. 2020;50(10):1117-25.
198. Hanna GJ, Ruiz ES, LeBoeuf NR, Thakuria M, Schmults CD, Decaprio JA, et al. Real-world outcomes treating patients with advanced cutaneous squamous cell carcinoma with immune checkpoint inhibitors (CPI). British Journal of Cancer. 2020;123(10):1535-42.
199. Hilke FJ, Sinnberg T, Gschwind A, Niessner H, Demidov G, Amaral T, et al. Distinct mutation patterns reveal melanoma subtypes and influence immunotherapy response in advanced melanoma patients. Cancers. 2020;12(9):1-15.
200. Singhi AD, George B, Greenbowe JR, Chung J, Suh J, Maitra A, et al. Real-Time Targeted Genome Profile Analysis of Pancreatic Ductal Adenocarcinomas Identifies Genetic Alterations That Might Be Targeted With Existing Drugs or Used as Biomarkers. Gastroenterology. 2019;156(8):2242-53.e4.
201. Singal G, Miller PG, Agarwala V, Li G, Kaushik G, Backenroth D, et al. Association of Patient Characteristics and Tumor Genomics With Clinical Outcomes Among Patients With Non-Small Cell Lung Cancer Using a Clinicogenomic Database. JAMA. 2019;321(14):1391-9.
202. Noroxe DS, Yde CW, Ostrup O, Michaelsen SR, Schmidt AY, Kinalis S, et al. Genomic profiling of newly diagnosed glioblastoma patients and its potential for clinical utility - a prospective, translational study. Molecular Oncology. 2020;14(11):2727-43.
203. Wang B, Li F, Zhou X, Ma Y, Fu W. Is microsatellite instability-high really a favorable prognostic factor for advanced colorectal cancer? A meta-analysis. World Journal of Surgical Oncology. 2019;17 (1) (no pagination)(169).

204. Deng Z, Qin Y, Wang J, Wang G, Lang X, Jiang J, et al. Prognostic and predictive role of DNA mismatch repair status in stage II-III colorectal cancer: A systematic review and meta-analysis. Clinical Genetics. 2020;97(1):25-38.
205. Song Y, Wang L, Ran W, Li G, Xiao Y, Wang X, et al. Effect of Tumor Location on Clinicopathological and Molecular Markers in Colorectal Cancer in Eastern China Patients: An Analysis of 2,356 Cases. Frontiers in Genetics. 2020;11 (no pagination)(96).
206. Williams DS, Mouradov D, Newman MR, Amini E, Nickless DK, Fang CG, et al. Tumour infiltrating lymphocyte status is superior to histological grade, DNA mismatch repair and BRAF mutation for prognosis of colorectal adenocarcinomas with mucinous differentiation. Modern Pathology. 2020;33(7):1420-32.
207. Lee CT, Chow NH, Chen YL, Ho CL, Yeh YM, Lin SC, et al. Clinicopathological features of mismatch repair protein expression patterns in colorectal cancer. Pathology Research and Practice. 2021;217 (no pagination)(153288).
208. Kunnackal John G, Das Villgran V, Caufield-Noll C, Giardiello F. Worldwide variation in lynch syndrome screening: case for universal screening in low colorectal cancer prevalence areas. Familial Cancer. 2020.
209. Salem ME, Yin J, Goldberg RM, Pederson LD, Wolmark N, Alberts SR, et al. Evaluation of the change of outcomes over a 10-year period in patients with stage III colon cancer: pooled analysis of 6501 patients treated with fluorouracil, leucovorin, and oxaliplatin in the ACCENT database. Annals of Oncology. 2020;31(4):480-6.
210. O'Connell E, Reynolds IS, McNamara DA, Prehn JHM, Burke JP. Microsatellite instability and response to neoadjuvant chemoradiotherapy in rectal cancer: A systematic review and meta-analysis. Surgical Oncology. 2020;34:57-62.
211. Australian Institute of Health and Welfare (AIHW) 2021 Cancer Data in Australia; Canberra: AIHW. <https://www.aihw.gov.au/reports/cancer/cancer-data-in-australia/>.
212. Arnold M, Rutherford M, Lam F, Bray F, Ervik M, Soerjomataram I (2019). ICBP SURVMARK-2 online tool: International Cancer Survival Benchmarking. Lyon, France: International Agency for Research on Cancer. Available from: http://gco.iarc.fr/survival/survmark, accessed [14/01/2022].
213. Luchini C, Bibeau F, Ligtenberg MJL, Singh N, Nottegar A, Bosse T, et al. ESMO recommendations on microsatellite instability testing for immunotherapy in cancer, and its relationship with PD-1/PD-L1 expression and tumour mutational burden: a systematic review-based approach. Annals of oncology : official journal of the European Society for Medical Oncology. 2019;30(8):1232-43.
214. Vanderwalde A, Spetzler D, Xiao N, Gatalica Z, Marshall J. Microsatellite instability status determined by next-generation sequencing and compared with PD-L1 and tumor mutational burden in 11,348 patients. Cancer Medicine. 2018;7(3):746-56.
215. Lee DW, Han SW, Bae JM, Jang H, Han H, Kim H, et al. Tumor mutation burden and prognosis in patients with colorectal cancer treated with adjuvant fluoropyrimidine and oxaliplatin. Clinical Cancer Research. 2019;25(20):6141-7.
216. Echejoh G, Liu Y, Chung-Faye G, Charlton J, Moorhead J, Clark B, et al. Validity of whole genomes sequencing results in neoplasms in precision medicine. Journal of Clinical Pathology. 2020.
217. Cho J, Ahn S, Son DS, Kim NKD, Lee KW, Lee J, et al. Bridging genomics and phenomics of gastric carcinoma. International Journal of Cancer. 2019;145(9):2407-17.
218. Fabrizio DA, George TJ, Dunne RF, Frampton G, Sun J, Gowen K, et al. Beyond microsatellite testing: Assessment of tumor mutational burden identifies subsets of colorectal cancer who may respond to immune checkpoint inhibition. Journal of Gastrointestinal Oncology. 2018;9(4):610-7.
219. Salem ME, Puccini A, Grothey A, Raghavan D, Goldberg RM, Xiu J, et al. Landscape of tumor mutation load, mismatch repair deficiency, and PD-L1 expression in a large patient cohort of gastrointestinal cancers. Molecular Cancer Research. 2018;16(5):805-12.

220. Weinberg BA, Xiu J, Hwang JJ, Shields AF, Salem ME, Marshall JL. Immuno-Oncology Biomarkers for Gastric and Gastroesophageal Junction Adenocarcinoma: Why PD-L1 Testing May Not Be Enough. The oncologist. 2018;23(10):1171-7.
221. Harthimmer MR, Stolborg U, Pfeiffer P, Mortensen MB, Fristrup C, Detlefsen S. Mutational profiling and immunohistochemical analysis of a surgical series of ampullary carcinomas. Journal of Clinical Pathology. 2019;72(11):762-70.
