# Supplementary material for: A scoping review and meta-analysis on the prevalence of pan-tumour biomarkers (dMMR, MSI, high TMB) in different solid tumours
Source: Sci Rep. 2022 Nov 28;12:20495. doi: 10.1038/s41598-022-23319-1 (PMC9705554; doi:10.1038/s41598-022-23319-1)
Supplement: Supplementary file 2 — Supplementary Information 2. [file 41598_2022_23319_MOESM2_ESM.pdf]

A scoping review and meta-analysis on the prevalence of pan-tumour biomarkers (dMMR, MSI, high TMB) in different solid tumours

Appendix II. Managing overlap of data sources for meta-analyses and studies with overlapping data sources

RUNNING TITLE: Prevalence of dMMR, MSI and high TMB in different solid cancers

CORRESPONDENCE: Dr Julia Steinberg

The Daffodil Centre

Tel: + 61293080283; Fax: + 61 2 8302 3550

Email: julia.steinberg@nswcc.org.au

Table of contents

1. Managing overlap of data sources for meta-analyses 3

2. Studies with overlapping data sources and the rationale for their inclusion or exclusion in meta-analyses 4

1) Studies reporting the prevalence of dMMR/MSI/high TMB based on data from the Cancer Genome Atlas 4

2) Studies reporting the prevalence of dMMR/MSI/high TMB based on data from the Foundation Medicine Database 5

3) Studies reporting the prevalence of dMMR/MSI/high TMB based on data from the Memorial Sloan Kettering Cancer Center 10

4) Studies reporting the prevalence of dMMR/MSI/high TMB based on data from the Dana-Farber Cancer Institute 12

5) Studies reporting the prevalence of dMMR/MSI/high TMB based on data from Caris Life Science 13

6) Systematic reviews and/or meta-analyses reporting the prevalence of dMMR/MSI/high TMB 15

7) Pooled analyses of clinical trials or genomic datasets reporting the prevalence of dMMR/MSI/high TMB 18

References 20

1. Managing overlap of data sources for meta-analyses

a) Some patient cohorts have been included in biomarker prevalence estimates in multiple original research studies or systematic reviews. To avoid data duplication in the meta-analyses in this review, we identified studies with overlapping data sources and only included at most one estimate based on a specific patient cohort.

b) For each biomarker and each major data source that was included multiple original research studies on the prevalence of dMMR/MSI/high TMB in specific cancer(s) and pan-cancer analyses (e.g., Foundation Medicine database, Memorial Sloan Kettering Cancer Centre patients, analysis of the Cancer Genome Atlas), we used the following approach.
   - Pan-cancer overall prevalence: the study with the largest sample size was included in the meta-analysis.
   - Cancer-specific overall prevalence: the study with the largest sample size for the specific cancer was included in the meta-analysis for “overall” cancer-specific estimates.
   - Cancer-specific prevalence for early-stage or advanced-stage cancers: the study with the largest sample size for the specific cancer type and stage was included in the meta-analysis.

c) Systematic reviews and/or meta-analyses
   - For some cancers (e.g., colorectal cancer, endometrial cancer), we identified multiple systematic reviews and/or meta-analyses that reported the prevalence of dMMR/MSI/high TMB to address a specific research question. For each cancer type, we included the study with the largest sample size. If the sample size was similar across multiple systematic reviews and/or meta-analyses, we included the study for which the research question was most aligned with our scoping review.
   - To avoid further data duplication, if a meta-analysis in this review included estimates from a previously published meta-analysis, the underlying original studies included in the previously published meta-analysis were excluded from the corresponding meta-analysis in this review.

See Section 2 below for the list of studies with overlapping data sources that reported the prevalence of dMMR/MSI/high TMB and the rationale for their inclusion or exclusion in meta-analyses.

2. Studies with overlapping data sources and the rationale for their inclusion or exclusion in meta-analyses

1) Studies reporting the prevalence of dMMR/MSI/high TMB based on data from the Cancer Genome Atlas

Author (year)	Cancer(s)	Cancer sub-group(s)	Cancer stage(s)	Data collection period and sample size[a]	Pan-tumour biomarker(s)	Rationale for inclusion/exclusion
Fan (2020)[1]	Gastric cancer		Overall	• Period N/S<br>• Total (N=924)<br>○ TCGA (n=440)<br>○ Local hospitals (n=484)	• TMB<br>• MSI	• The prevalence of high TMB was not included in the data synthesis due to a data-driven high TMB cut-off (75th percentile)<br>• The combined prevalence of MSI from both the TCGA and two local hospitals were included in the data synthesis<br>○ The TCGA cohort is larger than that included in Qu et al.[2] and Ren et al.[3]
Qu et al. (2020)[2]	Gastric cancer		Overall	• Period N/S<br>• Total (N=386)	• MSI	• Excluded from the data synthesis due to the smaller sample size than Fan et al.[1]
Ren et al. (2020)[3]	Gastric cancer		Overall	• Period N/S<br>• Total (N=383)	• TMB<br>• MSI	• Prevalence of TMB was provided in graphical format only and excluded from the data synthesis<br>• Prevalence of MSI was excluded from the data synthesis due to the smaller sample size than Fan et al.[1]
Li (2020)[4]	Gastric cancer		Overall	• Period N/S<br>• Total (N=510)<br>○ TCGA (n=210)<br>○ GEO (n=300)	• MSI	• Excluded from the data synthesis<br>○ The TCGA cohort is likely to be a subset of the cohort reported in Fan et al.[1]<br>○ The GEO cohort did not satisfy the minimum cancer-specific sample size cut-off (400+)
Dai (2020)[5]	Gastric cancer		IB-III	• Period N/S<br>• Total (N=424)<br>○ TCGA (n=202)<br>○ ACRG (n=138)<br>○ Local (n=89)	• MSI	• Included in the data synthesis for early-stage gastric cancers only

dMMR = mismatch repair deficiency; MSI = microsatellite instability; TMB = tumour mutational burden; N/S = not specified; TCGA = The Cancer Genome Atlas; ACGR = Asian Cancer Research Group; GEO = Gene Expression Omnibus.

[a] Number of samples available for the biomarker status.

2) Studies reporting the prevalence of dMMR/MSI/high TMB based on data from the Foundation Medicine Database

Author (year)	Cancer(s)	Cancer sub-group(s)	Cancer stage(s)	Data collection period and sample size[a]	Pan-tumour biomarker(s)	Rationale for inclusion/exclusion
Chan (2019)[6]	Pan-cancer	30 cancer types	Overall	• Period N/S<br>• Total (N=104,814)	• TMB (≥10 mut/Mb)	• Excluded from the data synthesis since the prevalence of high TMB was provided in graphical format only
Trabucco (2019)[7]	Pan-cancer	• 34 solid tumours<br>• ~10 haematopoietic tumours	Overall	• Period N/S<br>• Total (N=67,644)	• MSI	• Excluded from the data synthesis<br>○ Cancer-specific prevalence was shown only for cancer types with ≥100 samples, all of which had smaller sample size than Yoshino et al.[8]
Yoshino (2020)[8]	Pan-cancer	• 30 adult tumours<br>• 10 Paediatric tumours	Overall	• Period N/S<br>• Adult (N=164,410)<br>• Paediatric (N=3,592)	• MSI<br>• TMB (≥20 mut/Mb)	• Cancer-specific and pan-cancer prevalence of both MSI and of high TMB (≥20 mut/Mb) were included in the data synthesis due to the largest sample size, after excluding haematologic tumours and lymphoma
Huang (2021)[9]	Pan-cancer	• 6 tumour groups encompassing multiple common cancer types<br>• 9 cancer types	Overall	• Jan. 2016 - Nov. 2019<br>• Total (N=48,782)	• MSI<br>• TMB (≥10 mut/Mb)<br>• TMB (≥20 mut/Mb)	• Prevalence of MSI was excluded from the data synthesis due to the smaller sample size than Yoshino et al.[8]<br>• Prevalence of high TMB (≥10 mut/Mb) was included in the data synthesis<br>○ Cancer-specific prevalence: soft tissue sarcoma, melanoma, head and neck cancer, NSCLC, bladder/urothelial cancer, breast cancer, cervical cancer, endocrine tumour and neuroendocrine tumour (Note: Endocrine tumours were included in cancer-specific analysis due to thyroid cancer being the major cancer type. Neuroendocrine tumours are rare and were included in cancer-specific analysis)<br>○ Pan-cancer prevalence<br>○ Tumour group-specific prevalence: CNS tumours, gastrointestinal cancers, genitourinary tract cancers, gynaecological cancers, excluding esophageal SCC (rare histologic sub-type) and cancers not otherwise specified<br>• Prevalence of high TMB (≥20 mut/Mb) was excluded from the data synthesis due to the smaller sample size than Yoshino et al.[8]

Author (year)	Cancer(s)	Cancer sub-group(s)	Cancer stage(s)	Data collection period and sample size[a]	Pan-tumour biomarker(s)	Rationale for inclusion/exclusion
						• Specimens from metastatic site and primary tumour site represented 35.9% and 46.1%, respectively, with NSCLC representing 34.2% of the cohorts
Goodman (2019)[10]	Multiple cancer types	• SCC only	Overall	• Period N/S<br>• Total (N=12,058)<br>○ UCSD (n=2,651)<br>○ FM (n=9,407)	• MSI<br>• TMB (≥12 mut/Mb)<br>• TMB (≥20 mut/Mb)	• Excluded from the data synthesis<br>○ UCSD cohort: cancer-specific prevalence of MSI and high TMB were not provided<br>○ FM cohort: prevalence of high TMB in SCC only (lung, head and neck, esophageal, anal, cervical and urothelial SCC)
Parikh (2019)[11]	Gastrointestinal cancers	Tubular only	Advanced	• Period N/S<br>• Total (N=17,486)	• TMB (≥20 mut/Mb)	• Excluded from the data synthesis since this study focused on specific histologic sub-type only
Necchi (2020)[12]	Bladder/urothelial cancer	• Urothelial carcinoma<br>• SCC<br>• ADC	Advanced	• June 2012 – July 2018<br>• Total (N=2,368)<br>○ Urothelial carcinoma (n=2,142)<br>○ SCC (n=83)<br>○ ADC (n=143)	• MSI<br>• TMB (≥10 mut/Mb)<br>• TMB (≥20 mut/Mb)	• Included in the data synthesis since this study reported the prevalence of both MSI and high TMB (≥10 mut/Mb, ≥20 mut/Mb) in advanced bladder/urothelial cancers
Necchi (2020)[13]	Bladder/urothelial cancer	Urothelial carcinoma	Advanced	• Aug. 2014 – Nov. 2018<br>• Total (N=2,463)<br>○ Bladder (n=1,984)<br>○ Upper urinary tract (n=479)	• MSI	• Excluded from the data synthesis since Necchi et al.[12] reported the prevalence of both MSI and high TMB (≥10 mut/Mb, ≥20 mut/Mb) in advanced bladder/urothelial cancers<br>• MSI was enriched in upper urinary tract (3.3%, 16/479) relative to bladder cancer (0.8%, 15/1984) with overall prevalence 1.3% (31/2463)
Madison (2020)[14]	Bladder/urothelial cancer	Urothelial carcinoma	Advanced	• Period N/S<br>• Total (N=3,753)<br>○ Bladder (n=2,630)<br>○ Upper urinary tract (n=652)<br>○ Unspecified (n=471)	• MSI	• Excluded from the data synthesis since Necchi et al.[12] reported the prevalence of both MSI and high TMB (≥10 mut/Mb, ≥20 mut/Mb) in advanced bladder/urothelial cancers
Chung (2019)[15]	Prostate cancer		Overall	• Period N/S<br>• Total (N=3,476)<br>○ Primary site (n=1,660)	• MSI<br>• TMB (≥10 mut/Mb)	• Prevalence of both MSI and high TMB (≥20 mut/Mb) was excluded in the data synthesis due to the smaller sample size than Yoshino et al.[8]

Author (year)	Cancer(s)	Cancer sub-group(s)	Cancer stage(s)	Data collection period and sample size[a]	Pan-tumour biomarker(s)	Rationale for inclusion/exclusion
				○ Metastatic site (n=1,816)	• TMB (≥20 mut/Mb)	• Prevalence of high TMB (≥10 mut/Mb) was included in the data synthesis after combining estimates from both primary and metastatic site given metastatic sites include lymph node metastasis only and not limited to distant metastasis<br>○ Primary site (3.9%), metastatic site (6.2%)
Necchi (2020)[16]	Testicular cancer	Germ cell tumours only	Advanced (relapsed after CT)	• 2012 - 2017<br>• Total (N=107)<br>○ Seminoma (n=23)<br>○ Non-seminoma (n=84)	• MSI<br>• TMB (≥10 mut/Mb)<br>• TMB (≥20 mut/Mb)	• Included in the data synthesis after combining estimates from both seminomas and non-seminomas since this study focused on advanced testicular cancer only<br>• Patients who have experienced a relapse after at least one cisplatin-based CT regimen were included in the study, however tumour samples could have been obtained at any time during the treatment course and from any site of disease.
Patel (2020)[17]	Brain tumour<br>• Paediatric tumours only (age ≤ 21 years)	• Glioma<br>○ HGG<br>○ LGG<br>• MB<br>• Others	Overall	• Nov. 2012 – May 2017<br>• Total (N=723)<br>○ HGG (n=277)<br>○ LGG (n=235)<br>○ MB (n=134)<br>○ Others (n=77)	• TMB (≥20 mut/Mb)	• Excluded from the data synthesis<br>○ Likely to be a subset of the cohort reported by Yoshino et al.[8] (408 non-gliomas and 800 gliomas in paediatric patients)<br>○ Yoshino et al.[8] was the only one study reported the prevalence of dMMR/MSI/high TMB and data synthesis in the prevalence of the pan-tumour biomarkers in paediatric solid tumours was not performed
Chow (2020)[18]	Sarcoma	• Soft tissue sarcoma<br>○ DSRCT	Overall	• 2012-2018<br>• Total (N=83)	• MSI<br>• TMB (≥20 mut/Mb)	• Excluded from the data synthesis<br>○ Focused on a rare histologic sub-type<br>○ Yoshino et al. (2020)[8] reported the prevalence of TMB (≥20 mut/Mb) in soft tissue sarcoma
Eskander (2020)[19]	• Lung cancer<br>• Cervical cancer	• Lung: SCLC<br>• Cervix: HGNECC	Overall	• Mar. 2013 – Dec. 2017<br>• Total (N=1,800)<br>○ SCLC (n=1800)<br>○ HGNECC (n=97)	• MSI<br>• TMB (≥20 mut/Mb)	• Excluded from the data synthesis<br>○ SCLC: a sub-set of Foundation Medicine Database cohort reported by Yoshino et al. (2020)[8]<br>○ HGNECC: A rare histologic sub-type of cervical cancer

Author (year)	Cancer(s)	Cancer sub-group(s)	Cancer stage(s)	Data collection period and sample size[a]	Pan-tumour biomarker(s)	Rationale for inclusion/exclusion
Singhi (2019)[20]	Pancreatic cancer	Pancreatic ductal adenocarcinoma	Overall	• Period N/S<br>• Total (N=3,594)	• MSI<br>• TMB (≥20 mut/Mb)	• Prevalence of MSI was excluded from the data synthesis since this study was included in a systematic review by Luchini et al.[21]<br>• Prevalence of TMB (≥20 mut/Mb) was included in the data synthesis given Yoshino et al.[8] did not report the prevalence of TMB (≥20 mut/Mb) in pancreatic cancer
Huang (2020)[22]	Breast cancer	• HR+/HER2-<br>• HER2-<br>• TNBC	Overall	• Mar. 2019s – June 2019<br>• Total (N=312)	• TMB (≥9 mut/Mb)	• Exclude in the data synthesis due to the uncommon high TMB cut-off
Sivapiragasam (2021)[23]	Breast cancer	• ER+/HER2-<br>• ER-/HER2+<br>• TNBC	Metastatic	• Sep. 2012 – July 2018<br>• Total (N=3,831)<br>○ ER+/HER2- (n=1,237)<br>○ ER-/HER2+ (n=1,953)<br>○ TNBC (n=641)	• MSI<br>• TMB (≥10 mut/Mb)<br>• TMB (≥20 mut/Mb)	• Included in the data synthesis given this study focused on metastatic breast cancer only, after combining the estimates of any molecular subtypes<br>○ MSI-H: ER+/HER2- (2/1237, 0.2%), ER-/HER2/amp (2/1953, 0.1%), TNBC (3/641, 0.5%; reported % is 0.4%)<br>○ TMB≥10 mut/Mb: ER+/HER2- (99/1237, 8%), ER-/HER2+ (234/1953, 12%), TNBC (58/641, 9%)<br>○ TMB≥20 mut/Mb: ER+/HER2- (25/1237, 2%), ER-/HER2/amp (39/1953, 2%), TNBC (19/641, 3%)
Ross (2020)[24]	Cancer of unknown primary		Overall	• Period N/S<br>• Total (N=303)	• MSI<br>• TMB (≥16 mut/Mb)	• Excluded from the data synthesis due to the smaller sample size than Yoshino et al.[8]
Shao (2020)[25]	Multiple cancer types (same 10 rare solid tumour types included in KEYNOTE 158 study)	• Lung (SCLC, mesothelioma)<br>• Cervical cancer<br>• Anal cancer<br>• Vulvar cancer<br>• Endometrial cancer<br>• Biliary tract cancer<br>• Thyroid cancer<br>• Salivary gland carcinoma	Overall	• ~July 2018<br>• Total (N=2,992)	• TMB (≥10 mut/Mb)	• Excluded from the data synthesis<br>○ The cohort was generated by linking the Flatiron Health electronic health records database to the Foundation Medicine database of tumour sequencing results (Flatiron Health-Foundation Medicine Clinicogenomic Database), which is likely to be a sub-set of the study cohort reported by Huang et al.[9]

Author (year)	Cancer(s)	Cancer sub-group(s)	Cancer stage(s)	Data collection period and sample size[a]	Pan-tumour biomarker(s)	Rationale for inclusion/exclusion
		• Neuroendocrine tumour				
Singal (2019)[26]	Lung cancer	NSCLC	Advanced	• Jan. 2011 – Jan. 2018<br>• Total (N=4,064)	• TMB (≥20 mut/Mb)	• Included in the data synthesis since the study focused on advanced NSCLC only, although the study cohort was identified from the Flatiron Health-Foundation Medicine Clinicogenomic Database
Okamura (2020)[27]	9 cancer types associated with high ARID1A alterations (>5%)	• Lung cancer<br>• Colorectal cancer<br>• Breast cancer<br>• Melanoma<br>• Pancreatic cancer<br>• Cholangiocarcinoma /hepatocellular<br>• Gastric / esophageal cancer<br>• Endometrial cancer<br>• Urothelial bladder carcinomas	Overall	• Period N/S<br>• Total (N=1,093)	• MSI<br>• TMB (≥20 mut/Mb)	• Excluded from the data synthesis<br>○ Tissue DNA from the UCSD was analysed by Foundation Medicine, which is likely to be a sub-set of the study cohort reported by Yoshino et al.[8]<br>○ Even if this is not a sub-set of the Foundation Medicine cohort, inclusion of this study will not make substantial difference due to small cancer-specific sample size. For example, sample size for colorectal, endometrial, bladder and gastric/esophageal cancer are below the minimum sample size cut-off.

dMMR = mismatch repair deficiency; MSI = microsatellite instability; TMB = tumour mutational burden; N/S = not specified; CNS = central nervous system ; SCC = squamous cell carcinoma; ADC = adenocarcinoma; NSCLC = non-small cell lung cancer; UCSD = University of California San Diego; FM = Foundation Medicine; HGG = high grade glioma; LGG = low grade glioma; MB = medulloblastoma; CT = chemotherapy; DSRCT = desmoplastic small round cell tumour; SCLC = small cell lung cancer; HGNECC = high grade neuroendocrine cervical cancer; HR+ = hormone receptor positive; HER2- = human epidermal growth factor receptor negative; TNBC = triple negative breast cancer; UCSD = University of California San Diego; CRC = colorectal cancer.

[a] Number of samples available for the biomarker status.

3) Studies reporting the prevalence of dMMR/MSI/high TMB based on data from the Memorial Sloan Kettering Cancer Center

Author (year)	Cancer(s)	Cancer sub-group(s)	Cancer stage(s)	Data collection period and sample size[a]	Pan-tumour biomarker(s)	Rationale for inclusion/exclusion
Latham (2019)[28]	Pan-cancer	50+ cancer types	Overall	• Jan. 2014 – June 2017<br>• Total (N=15,045)	• MSI	• Excluded from the data synthesis<br>○ Pan-cancer prevalence: Hechtman et al.[29] reported the similar pan-cancer prevalence of MSI with the larger sample size (2.2% [326/15045] vs 2.0% [582/29530])<br>○ Cancer-specific prevalence: Prevalence of high-frequency (MSI-H) or indeterminate microsatellite instability (MSI-L) was reported. Prevalence of MSI-H and MSI-L was separately presented in graphical format only<br>• Breast (n=2,371) and lung (n=1,952) cancers represent 28.7% of tumours and CRC and EC represent 9% of all tumours (n=1,351)
Hechtman (2020)[29]	Pan-cancer		Overall	• 2014 - 2018<br>• Total (N=29,530)<br>○ Sub-group (n=443)	• MSI	• Pan-cancer prevalence of MSI only was included in the data synthesis due to the largest sample size<br>○ Cancer-specific prevalence was not provided since this study focused on a sub-group whose IHC results for dMMR was available. Discordance between MSI and dMMR was 7.2% (overall), 6.4%(CRC and 4.9% (EC)
Valero (2021)[30]	Pan-cancer	17 cancer types	Overall	• Period N/S<br>• Total (N=10,233)	• MSI<br>• TMB (percentiles)	• Excluded from the data synthesis<br>○ Prevalence of MSI: pan-cancer prevalence (3%, 264/10233) was provided with the smaller sample size than reported by Hechtman et al.[29]<br>○ Prevalence of high TMB: data driven high TMB cut-off (percentiles) was used
Jimenez-Rodriguez (2020)[31]	Colon cancer	Adenocarcinoma	I/II/III	• Feb. 2007 – Dec. 2014<br>• Total (N=443)	• dMMR	• Included in the data synthesis since this study focused on early-stage colon cancer only
Middah (2019)[32]	CRC		Overall	• Jan. 2014 – Oct. 2017<br>• Total (N=1,751)	• MSI	• Included in the data synthesis
Greally (2019)[33]	Esophagogastric cancer	• Esophageal/GE junction cancer<br>• Gastric cancer	Metastatic	• Sep. 2013 – May 2018<br>• Total (N=161)<br>○ Esophageal/GE junction (n=85)	• dMMR/MSI	• Excluded from the data synthesis<br>○ Prevalence of either dMMR or MSI measured by IHC or selected gene panel testing was provided without specifying denominators

Author (year)	Cancer(s)	Cancer sub-group(s)	Cancer stage(s)	Data collection period and sample size[a]	Pan-tumour biomarker(s)	Rationale for inclusion/exclusion
				○ Gastric (n=76)		
Audenet (2019)[34]	• Bladder/urothelial cancer	Urothelial carcinoma only	Overall	• Period N/S<br>• Total (N=649)<br>○ Bladder (n=454)<br>○ Upper urinary tract (n=194)	• MSI	• Included in the data synthesis combining estimates from both bladder and upper urinary tract urothelial carcinomas<br>○ MSI was enriched in upper urinary tract (6.2% 12/194) relative to bladder cancer (0.9%, 4/454) with overall prevalence 2.5% (16/648)
Carlo (2019)[35]	Kidney cancer	Renal cell carcinoma only	Metastatic	• Apr. 2014 – Jan. 2017<br>• Total (N=115)	• MSI	• Included in the data synthesis since this study focused on metastatic renal cell carcinoma only, which is the most common histologic sub-type of kidney cancer
Abida (2019)[36]	Prostate cancer		Overall	• Jan. 2015 – Jan. 2018<br>• Total (N=1,551 from 1,346 patients)	• MSI<br>• TMB (≥10 mut/Mb)	• Included in the data synthesis<br>○ Prevalence of MSI was reported separately for CRPC and non-CRPC: combined prevalence from both CRPC (4.5%, 16/356) and non-CRPC (2.4%, 16/677) cases were included in the data synthesis<br>○ Prevalence of TMB (≥10 mut/Mb) separately for CRPC and non-CRPC was not reported, and the overall prevalence was included in the data synthesis
Liu (2020)[37]	Ovarian cancer		Advanced (mostly recurrent III/IV)	• Jan. 2013 – Apr. 2019<br>• Total (N=64)	• MSI<br>• TMB (≥10 mut/Mb)	• Included in the data synthesis since the study included advanced ovarian cancer cases only
Stasenko (2020)[38]	Endometrial cancer	Endometrioid carcinoma	IA	• Jan. 2009 – Feb. 2017<br>• Total (N=211)	• dMMR	• Included in the data synthesis since this study focused on stage IA endometrial carcinoma only, which is the most common histologic sub-type of endometrial cancer

dMMR = mismatch repair deficiency; MSI = microsatellite instability; TMB = tumour mutational burden; N/S = not specified; CRC = colorectal cancer; EC = endometrial cancer; IHC = immunohistochemistry; GE junction = gastroesophageal junction; CRPC = castration-resistant prostate cancer

[a] Number of samples available for the biomarker status.

4) Studies reporting the prevalence of dMMR/MSI/high TMB based on data from the Dana-Farber Cancer Institute

Author (year)	Cancer(s)	Cancer sub-group(s)	Cancer stage(s)	Data collection period and sample size[a]	Pan-tumour biomarker(s)	Rationale for inclusion/exclusion
Albayrak (2020)[39]	Pan-cancer	50+ solid tumours	Overall	• Aug. 2013 – July 2018<br>• Total (n=18,709, excluding haematologic tumours, lymphomas and benign lesions)	• dMMR	• Included in the data synthesis after excluding cancer types that do not satisfy the minimum sample size threshold<br>○ Haematologic tumours, lymphomas and benign lesions were also excluded<br>○ Prevalence predicted by an algorithm was included in the data synthesis given high concordance between the algorithm-based prevalence and the historical reports by pathologists (n=4,404)
Doyle (2019)[40]	Sarcoma	• Soft tissue sarcoma	Overall	• Period (N/S)<br>• Total (N=304)<br>○ Classified (n=264)<br>○ Unclassified (n=40)	• dMMR	• Exclude from the data synthesis since this study cohort is a subset of the cohort reported in Albayrak et al.[39]<br>○ Focused on reporting the different prevalence of dMMR between unclassified sarcomas (4/40, 10.0%) and classified sarcomas (3/264, 1.1%)
Christakis (2019)[41]	• Upper GI cancers<br>• Biliary tract cancers	Cancers in the<br>• Small bowel<br>• Stomach<br>• Esophageal<br>• Pancreas<br>• Bile duct<br>• Gallbladder<br>• Ampulla	Overall	• Period (N/S)<br>• Total (N=645)<br>○ Small bowel (n=29)<br>○ Stomach (n=97)<br>○ Esophageal (n=230)<br>○ Pancreas (n=199)<br>○ Bile duct (n=60)<br>○ Gallbladder (n=19)<br>○ Ampulla (n=11)	• dMMR	• Prevalence in esophageal (incl. gastroesophageal junction cancers) only was included in the data synthesis<br>○ Albayrak et al.[39] reported the prevalence in esophagogastric cancers including both gastric cancers and esophageal cancers<br>○ Sample size of stomach cancer in this study does not satisfy the minimum sample size threshold (400+)<br>• Prevalence in other cancer types was excluded from the data synthesis due to the smaller sample size than Albayrak et al.[39]
Nassar (2019)[42]	• Bladder/urothelial cancer	Urothelial carcinoma only	• Overall<br>• Stage-specific	• 2013 – 2017<br>• Total (N=310)<br>○ Upper urinary tract (n=53)<br>○ Bladder (n=257)	• TMB (≥10 mut/Mb)<br>• TMB (≥20 mut/Mb)	• Included in the data synthesis since Albayrak et al.[39] did not report the prevalence of high TMB (≥10 mut/Mb or ≥20 mut/Mb)<br>○ A total of 162 T0 cases were excluded from the data synthesis

dMMR = mismatch repair deficiency; MSI = microsatellite instability; TMB = tumour mutational burden; N/S = not specified.

[a] Number of samples available for the biomarker status.

5) Studies reporting the prevalence of dMMR/MSI/high TMB based on data from Caris Life Science

Author (year)	Cancer(s)	Cancer sub-group(s)	Cancer stage(s)	Data collection period and sample size[a]	Pan-tumour biomarker(s)	Rationale for inclusion/exclusion
Nikanjam (2020)[43]	Pan-cancer	40 tumour types	Overall	• Feb 2015 – Nov 2017<br>• Total (N=28,034, excl. haematologic tumours, lymphomas and benign tumours)<br>○ MSI (n=28,034)<br>○ TMB (n=27,847)	• MSI<br>• TMB (≥17 mut/Mb)	• Prevalence of MSI was included in the data synthesis, excluding haematologic tumours, lymphomas ad benign tumours<br>○ Pan-cancer prevalence<br>○ Cancer-specific prevalence for those satisfying the minimum sample size threshold, except for the following cancer types<br>▪ Biliary tract cancer: Spizzo et al.[44] reported the prevalence with the bigger sample size<br>▪ Male genital tract malignancy, female genital tract malignancy: neither cancer-specific nor tumour group-specific given prostate cancer and ovarian cancer were reported, respectively<br>▪ Uveal melanoma: this is a rare subtype of skin cancer<br>• Prevalence of high TMB was included in the data synthesis in pan-caner setting only due to the uncommon high TMB cut-off<br>• No. of tumours with MSI and high-TMB were calculated based on the total no. of tumours and the reported prevalence in each cancer type (≥17 mut/Mb)
Spizzo (2020)[44]	Biliary tract cancer		Overall	• June 2014 – Jan. 2019<br>• Total (N=1,292)	• MSI<br>• TMB (≥17 mut/Mb)	• Prevalence of MSI was included in the data synthesis due to the larger sample size than Nikanjam et al.[43]<br>• Prevalence of high TMB was excluded from the data synthesis in pan-caner setting only due to the uncommon high TMB cut-off
Tokunaga (2019)[45]	Appendiceal cancer	Adenocarcinoma	Overall	• Apr. 2015 – Jan. 2018<br>• Total (N=183)	• MSI<br>• TMB (≥17 mut/Mb)	• Prevalence of MSI was included in the data synthesis<br>○ Appendiceal cancer was not reported I Nikanjam et al.[43]<br>• Prevalence of high TMB was excluded from the data synthesis due to the uncommon high TMB cut-off
Cimic (2020)[46]	Cervical cancer	• NECC<br>• SCC	• Overall	• Period N/S<br>• Total (N=661)<br>○ NECC (n=62)<br>○ SCC (n=599)	• MSI<br>• TMB (≥17 mut/Mb)	• Prevalence of MSI, combined both NECC (0/31, 0%) SCC (6/599, 1.0%), was included in the data synthesis<br>○ Cervical cancer was not reported in Nikanjam et al.[43]<br>• Prevalence of high TMB was excluded due to the uncommon high TMB cut-off

Author (year)	Cancer(s)	Cancer sub-group(s)	Cancer stage(s)	Data collection period and sample size[a]	Pan-tumour biomarker(s)	Rationale for inclusion/exclusion
Jones (2020)[47]	Endometrial cancer	• Endometrioid carcinoma	• Overall	• Period N/S<br>• Total (N=621)	• MSI<br>• TMB (≥10 mut/Mb)	• Prevalence of both MSI and TMB (≥10 mut/Mb) were included in the data synthesis<br>○ Endometrial cancer was not reported in Nikanjam et al.[43]
Seeber (2020)[48]	Pancreatic cancer	Pancreatic ductal adenocarcinoma	• Overall	• Apr. 2015 – Jan. 2018<br>• Total (N=2,818)	• dMMR/MSI	• Excluded from the data synthesis<br>○ Prevalence of either dMMR or MSI was 1.3% in the tested tumours, but the number of tested tumours were not provided<br>○ Nikanjam et al.[43] reported the similar prevalence of MSI (1.4%, 18/1261), and was included in the data synthesis
Stein (2019)[49]	Lung cancer	• NSCLC	• Advanced	• 2015-2017<br>• Total (N=3,424)	• TMB (≥10 mut/Mb)	• Included in the data synthesis
Heeke (2020)[50]	Breast cancer	• HR-MT<br>• HR-WT	Overall	• Feb. 2015 - Jan. 2019<br>• Total (N=4,562)<br>○ HR-MT (n=812)<br>○ HR-WT (n=3,750)	• dMMR/MSI<br>• TMB (≥10 mut/Mb)	• Prevalence of dMMR/MSI was excluded from the data synthesis<br>○ Multiple test platforms were used to measure dMMR/MSI including fragment analysis, IHC and NGS and the combined prevalence of dMMR/MSI (0.6%, 26/4562) was reported.<br>○ Nikanjam et al.[43] reported the similar prevalence of MSI measured by selected gene panel sequencing (0.7%, 17/2427) and was included in the data synthesis<br>• Prevalence of high TMB was included in the data synthesis combining the prevalence from both HR-MT (28.3%, 230/812) and HR-WT (19.4%, 728/3750)

dMMR = mismatch repair deficiency; MSI = microsatellite instability; TMB = tumour mutational burden; N/S = not specified; NECC = neuroendocrine cervical carcinoma; SCC = squamous cell carcinoma; NSCLC = non-small cell lung cancer; HR-MT = Homologous recombination DNA damage repair pathway mutated; HR-WT = Homologous recombination DNA damage repair pathway wild-type.

[a] Number of samples available for the biomarker status.

6) Systematic reviews and/or meta-analyses reporting the prevalence of dMMR/MSI/high TMB

Author (year)	Cancer(s)	Cancer stage(s)	Data collection period and sample size[a]	Pan-tumour biomarker(s)	Rationale for inclusion/exclusion
Lorenzi (2020)[51]	• Colorectal cancer<br>• Endometrial cancer<br>• Ovarian cancer<br>• Gastric cancer<br>• Esophageal cancer	• dMMR: Overall<br>• MSI<br>○ Overall<br>○ I/II, III/IV (sub-group)	• ~ Oct. 2017	• dMMR<br>• MSI	• Pooled overall and stage-specific prevalence of dMMR and MSI across all tumours reported in this structured/targeted review were not included in the data synthesis<br>• Overall cancer-specific prevalence of dMMR (ovarian cancer, gastric cancer) and prevalence of MSI (ovarian cancer, gastric cancer, and esophageal cancer) only were included in the data synthesis due to different stage grouping from this scoping review<br>• Prevalence of dMMR and MSI in colorectal cancer and endometrial cancer were excluded from the data synthesis<br>○ Colorectal cancer (dMMR: 13.2%, 1513/11434; MSI: 11.5%, 937/8156): prevalence estimates were obtained through targeted review, and a systematic review by Jin et al.[52] was included in the data synthesis<br>○ Endometrial cancer (dMMR: 24.8%, 1302/5248; MSI: 26.0%, 1773/6813): Ryan et al.[53] was the most up-to-date systematic review of endometrial cancer and the research questions aligns better with this scoping review, and this study was included in the data synthesis
Luchini (2020)[21]	Pancreatic ductal adenocarcinoma	Overall	• ~30/11/2019<br>• Total (N=8,323 cases from 34 studies)	• dMMR/MSI<br>○ MSI by NGS<br>○ dMMR/MSI by IHC/PCR	• Prevalence of MSI measured by selected gene panel sequencing alone was included in the data synthesis<br>○ Statistically significant difference in the prevalence by assays used: gene panel sequencing (1.1%, 68/6030) vs IHC/PCR (6.5%, 150/2293)<br>○ Most included studies used PCR for MSI analysis was not with recommended panel of markers (nor NCI neither MSI PCR).<br>○ Included studies often reported the combined prevalence of dMMR/MSI by IHC/PCR<br>○ Of the included studies, three studies were published in 2019 (Latham et al.[28], Singhi et al.,[20] and Kato et al.[54]), of which the study period overlaps with this scoping review. All three studies were also identified in this scoping review, and the prevalence estimates from these original research studies were not included in the data synthesis

Author (year)	Cancer(s)	Cancer stage(s)	Data collection period and sample size[a]	Pan-tumour biomarker(s)	Rationale for inclusion/exclusion
Ryan (2019)[53]	Endometrial cancer	Overall	• ~ July 2018<br>• Total (N=12,633 cases from 53 studies)	• dMMR<br>• MSI	• Included in the data synthesis<br>○ Prevalence of dMMR<br>○ Prevalence of MSI
Kahn (2019)[55]	Endometrial cancer	• dMMR<br>○ Overall<br>○ I, II, III, IV (sub-group)<br>• MSI<br>○ Overall	• Jan. 1990 - Jan. 2018<br>• Total (N=6,649 cases from 29 studies)<br>○ dMMR (n=6,649)<br>○ MSI (n=3,140)	• dMMR<br>• MSI	• Prevalence of dMMR in early-stage endometrial cancer only was included in the data synthesis<br>○ Likely to be a subset of studies included in a systematic review by Ryan et al.[53]<br>○ Prevalence in advanced-stage endometrial cancer does not satisfy the minimum sample size threshold (n=24)
Jin (2020)[52]	Colorectal cancer	• Overall	• 2007 – July 2018<br>• Total (N=17,621 from 44 studies)	• dMMR<br>• MSI	• Included in the data synthesis due to separate reporting of the prevalence of dMMR and MSI, although the sample size was smaller than John et al.[56]
John (2020)[56]	Colorectal cancer	• Overall	• 2005 – 2017<br>• Total (N=47,545 from 73 studies)	• dMMR/MSI	• Included in the data synthesis for the combined prevalence of dMMR and MSI<br>○ High concordance between dMMR and MSI in colorectal cancer<br>○ Research question is aligned with this scoping review: Systematic review of studies performing universal screening for LS
Wang (2019)[57]	Colorectal cancer	• III<br>• IV	• ~ July 2018<br>• Total (N=21,175 from 36 studies)<br>○ Stage III (n=18,277)<br>○ Stage IV (n=2,898)	• dMMR/MSI	• Included in the data synthesis for the combined prevalence of dMMR and MSI<br>○ Stage-specific prevalence was reported<br>○ High concordance between dMMR and MSI in colorectal cancer
Deng (2020)[58]	Colorectal cancer	II/III combined	• ~ May 2019<br>• Total (N=28,331 from 51 studies)	• dMMR	• Included in the data synthesis for early-stage colorectal cancer<br>○ Of the included studies, only one study was published in 2019 (Fountzilas et al.[59]), of which the study period overlaps with this scoping review. This study was also identified in this scoping review and the prevalence estimate from this original study was excluded from the data synthesis
O'Connell (2020)[60]	Rectal cancer	II/III combined	• ~ Aug. 2019<br>• Total (N=5,877 from 9 studies)	• dMMR/MSI	• Included in the data synthesis for the combined prevalence of dMMR and MSI<br>○ Stage-specific prevalence was reported<br>○ High concordance between dMMR and MSI in colorectal cancer

Author (year)	Cancer(s)	Cancer stage(s)	Data collection period and sample size[a]	Pan-tumour biomarker(s)	Rationale for inclusion/exclusion
					○ Of the included studies, only one study was published in 2019 (Meillan et al.[61]), of which the study period overlaps with this scoping review. This study was also identified in this scoping review and the prevalence estimate from this original study was excluded from the data synthesis
Willis (2019)[62]	Lung cancer<br>• NSCLC<br>• SCLC	Advanced	• Jan. 2012 ~ Apr. 2018<br>• Total (N=10,122 from 3 publications)<br>○ NSCLC (n=991)<br>○ SCLC (n=211)	• TMB (≥10 mut/Mb)	• Included in the data synthesis from 3 studies reported the prevalence of high TMB using a common high TMB cut-off (≥10 mut/Mb)<br>○ Two NSCLC studies: Checkmate 026, Checkmate 227<br>○ One SCLC study: Checkmate 032
Zhu (2019)[63]	Lung cancer<br>• NSCLC	Advanced	• ~ Oct. 2018<br>• Total (N= 2,661 from 8 studies)	• TMB (≥10 mut/Mb)	• Excluded from the data synthesis<br>○ Of the included studies, only two studies used the common high TMB cut-off TMB (>=10 mut/Mb or 200+ mutations from WES), and both of them were NSCLC studies that were also included in Willis et al.[62]

dMMR = mismatch repair deficiency; MSI = microsatellite instability; TMB = tumour mutational burden; N/S = not specified; IHC = immunohistochemistry; PCR= polymerase chain reaction; LS = Lynch syndrome; NSCLC = non-small cell lung cancer; SCLC = small cell lung cancer.

[a] Number of samples available for the biomarker status.

7) Pooled analyses of clinical trials or genomic datasets reporting the prevalence of dMMR/MSI/high TMB

Author (year)	Cancer(s)	Cancer stage(s)	Data collection period and sample size[a]	Pan-tumour biomarker(s)	Rationale for inclusion/exclusion
Salem (2020)[64]	Colon cancer	III	• 1998 - 2009<br>• Total (N=6,501)	• dMMR/MSI	• Included in the data synthesis for the combined prevalence of dMMR and MSI given high concordance between dMMR and MSI in colorectal cancer<br>○ Pooled analysis of patients from six adjuvant CT trials (MOSAIC, C07, C08, PETACC8, N0147, AVANT) treated with fluorouracil, leucovorin, and oxaliplatin and included in the ACCENT database<br>○ Larger sample size than Taieb et al.[65]
Taieb (2019)[65]	Colon cancer	III	• 1998 - 2009<br>• Total (N=2,630)	• dMMR/MSI	• Excluded from the data synthesis due to the smaller sample size than Salem et al.[64]<br>○ Pooled analysis of patients from six adjuvant CT trials (MOSAIC, C07, C08, PETACC8, N0147, AVANT) focused on those with disease recurrence following adjuvant treatment
Sinicrope (2021)[66]	Colon cancer	III	• 2004 - 2009<br>• Total (N=5,337)	• dMMR/MSI	• Excluded from the data synthesis<br>○ A subset of six adjuvant CT trials (MOSAIC, C07, C08, PETACC8, N0147, AVANT) including participants from PETACC8 and N0147 only
Pietrantonio (2019)[67]	Gastric cancer	II/III	• Period (N/S?)<br>• (N= 1,556 from 4 RCTs)	• MSI	• Included in the data synthesis<br>○ Meta-analysis of individual patient data from four RCTS (MAGIC, ITACA-S, ARTIST, CLASSIC) compared surgery with surgery + CT for resectable gastric cancer in four countries
Choi (2019)[68]	Gastric cancer	II/III	• Period (N/S?)<br>• Total (N= 592)	• MSI	• Excluded from the data synthesis<br>○ A subset of the study cohort reported in Pietrantonio et al.,[67] including participants from CLASSIC only
Barroso-Sousa (2020)[69]	Breast cancer	• Overall<br>○ Primary<br>○ Metastatic	• Period (N/S?)<br>• Total (N=3,951)<br>○ Primary cancer (n=2,455)<br>○ Metastatic cancer (n=1,496)	• TMB (≥10 mut/Mb)	• Included in the data synthesis<br>○ Genomic and clinical datasets from three WES studies and three targeted panel studies, including GENIE-DFCI-ONCOPANEL-3, GENIE-MSK IMPACT410, and GENIE-MSK IMPACT468.<br>○ Original research study from the MSK breast cancer cohort was not identified in this scoping review.<br>○ One original research study from the DFCI breast cancer cohort was identified in this scoping review and the prevalence of dMMR reported in Albayrak et al.[39] was included in the data synthesis

Author (year)	Cancer(s)	Cancer stage(s)	Data collection period and sample size[a]	Pan-tumour biomarker(s)	Rationale for inclusion/exclusion
					○ Overall prevalence using all the samples (50%, 196/3951) and the prevalence in advanced breast cancer using samples from metastatic cancers only (8.4%, 125/1496)

dMMR = mismatch repair deficiency; MSI = microsatellite instability; TMB = tumour mutational burden; N/S = not specified; CT = chemotherapy; RCT = randomised controlled trial; WES = whole exome sequencing; MSK = Memorial Sloan Kettering Cancer Centre; DFCI = Dana-Farber Cancer Institute.

[a] Number of samples available for the biomarker status.

References

1. Fan Y, Ying H, Wu X, Chen H, Hu Y, Zhang H, et al. The mutational pattern of homologous recombination (HR)-associated genes and its relevance to the immunotherapeutic response in gastric cancer. Cancer Biology and Medicine. 2020;17(4):1002-13.
2. Qu X, Zhao L, Zhang R, Wei Q, Wang M. Differential microRNA expression profiles associated with microsatellite status reveal possible epigenetic regulation of microsatellite instability in gastric adenocarcinoma. Annals of Translational Medicine. 2020;8 (7) (no pagination)(484).
3. Ren Q, Zhu P, Zhang H, Ye T, Liu D, Gong Z, et al. Identification and validation of stromal-tumor microenvironment-based subtypes tightly associated with PD-1/PD-L1 immunotherapy and outcomes in patients with gastric cancer. Cancer Cell International. 2020;20 (1) (no pagination)(92).
4. Li Y, Wang HC, Wang JS, Sun B, Li LP. Chemokine receptor 4 expression is correlated with the occurrence and prognosis of gastric cancer. FEBS Open Bio. 2020;10(6):1149-61.
5. Dai D, Zhao X, Li X, Shu Y, Shen B, Chen X, et al. Association Between the Microsatellite Instability Status and the Efficacy of Postoperative Adjuvant Chemoradiotherapy in Patients With Gastric Cancer. Frontiers in Oncology. 2020;9 (no pagination)(1452).
6. Chan TA, Yarchoan M, Jaffee E, Swanton C, Quezada SA, Stenzinger A, et al. Development of tumor mutation burden as an immunotherapy biomarker: Utility for the oncology clinic. Annals of Oncology. 2019;30(1):44-56.
7. Trabucco SE, Gowen K, Maund SL, Sanford E, Fabrizio DA, Hall MJ, et al. A Novel Next-Generation Sequencing Approach to Detecting Microsatellite Instability and Pan-Tumor Characterization of 1000 Microsatellite Instability-High Cases in 67,000 Patient Samples. Journal of Molecular Diagnostics. 2019;21(6):1053-66.
8. Yoshino T, Pentheroudakis G, Mishima S, Overman MJ, Yeh KH, Baba E, et al. JSCO-ESMO-ASCO-JSMO-TOS: international expert consensus recommendations for tumour-agnostic treatments in patients with solid tumours with microsatellite instability or NTRK fusions. Annals of Oncology. 2020;31(7):861-72.
9. Huang RSP, Haberberger J, Severson E, Duncan DL, Hemmerich A, Edgerly C, et al. A pan-cancer analysis of PD-L1 immunohistochemistry and gene amplification, tumor mutation burden and microsatellite instability in 48,782 cases. Modern pathology : an official journal of the United States and Canadian Academy of Pathology, Inc. 2021;34(2):252-63.
10. Goodman AM, Kato S, Chattopadhyay R, Okamura R, Saunders IM, Montesion M, et al. Phenotypic and genomic determinants of immunotherapy response associated with squamousness. Cancer Immunology Research. 2019;7(6):866-73.
11. Parikh AR, He Y, Hong TS, Corcoran RB, Clark JW, Ryan DP, et al. Analysis of DNA Damage Response Gene Alterations and Tumor Mutational Burden Across 17,486 Tubular Gastrointestinal Carcinomas: Implications for Therapy. The oncologist. 2019;24(10):1340-7.
12. Necchi A, Madison R, Raggi D, Jacob JM, Bratslavsky G, Shapiro O, et al. Comprehensive Assessment of Immuno-oncology Biomarkers in Adenocarcinoma, Urothelial Carcinoma, and Squamous-cell Carcinoma of the Bladder. European urology. 2020;77(4):548-56.
13. Necchi A, Madison R, Pal SK, Ross JS, Agarwal N, Sonpavde G, et al. Comprehensive Genomic Profiling of Upper-tract and Bladder Urothelial Carcinoma. European Urology Focus. 2020.
14. Madison RW, Gupta SV, Elamin YY, Lin DI, Pal SK, Necchi A, et al. Urothelial cancer harbours EGFR and HER2 amplifications and exon 20 insertions. BJU International. 2020;125(5):739-46.
15. Chung JH, Dewal N, Sokol E, Mathew P, Whitehead R, Millis SZ, et al. Prospective comprehensive genomic profiling of primary and metastatic prostate tumors. JCO Precision Oncology. 2019;3(no pagination).
16. Necchi A, Bratslavsky G, Corona RJ, Chung JH, Millis SZ, Elvin JA, et al. Genomic Characterization of Testicular Germ Cell Tumors Relapsing After Chemotherapy. European Urology Focus. 2020;6(1):122-30.

17. Patel RR, Ramkissoon SH, Ross J, Weintraub L. Tumor mutational burden and driver mutations: Characterizing the genomic landscape of pediatric brain tumors. Pediatric Blood and Cancer. 2020;67 (7) (no pagination)(e28338).
18. Chow WA, Yee JK, Tsark W, Wu X, Qin H, Guan M, et al. Recurrent secondary genomic alterations in desmoplastic small round cell tumors. BMC Medical Genetics. 2020;21 (1) (no pagination)(101).
19. Eskander RN, Elvin J, Gay L, Ross JS, Miller VA, Kurzrock R. Unique genomic landscape of high-grade neuroendocrine cervical carcinoma: Implications for rethinking current treatment paradigms. JCO Precision Oncology. 2020;4:972-87.
20. Singhi AD, George B, Greenbowe JR, Chung J, Suh J, Maitra A, et al. Real-Time Targeted Genome Profile Analysis of Pancreatic Ductal Adenocarcinomas Identifies Genetic Alterations That Might Be Targeted With Existing Drugs or Used as Biomarkers. Gastroenterology. 2019;156(8):2242-53.e4.
21. Luchini C, Brosens LAA, Wood LD, Chatterjee D, Shin JI, Sciammarella C, et al. Comprehensive characterisation of pancreatic ductal adenocarcinoma with microsatellite instability: Histology, molecular pathology and clinical implications. Gut. 2021;70(1):148-56.
22. Huang RSP, Li X, Haberberger J, Sokol E, Severson E, Duncan DL, et al. Biomarkers in Breast Cancer: An Integrated Analysis of Comprehensive Genomic Profiling and PD-L1 Immunohistochemistry Biomarkers in 312 Patients with Breast Cancer. The oncologist. 2020;25(11):943-53.
23. Sivapiragasam A, Ashok Kumar P, Sokol ES, Albacker LA, Killian JK, Ramkissoon SH, et al. Predictive Biomarkers for Immune Checkpoint Inhibitors in Metastatic Breast Cancer. Cancer Med. 2021;10(1):53-61.
24. Ross JS, Sokol ES, Moch H, Mileshkin L, Baciarello G, Losa F, et al. Comprehensive Genomic Profiling of Carcinoma of Unknown Primary Origin: Retrospective Molecular Classification Considering the CUPISCO Study Design. Oncologist. 2020.
25. Shao C, Li G, Huang L, Pruitt S, Castellanos E, Frampton G, et al. Prevalence of High Tumor Mutational Burden and Association With Survival in Patients With Less Common Solid Tumors. JAMA Network Open. 2020;3 (10) (no pagination)(e2025109).
26. Singal G, Miller PG, Agarwala V, Li G, Kaushik G, Backenroth D, et al. Association of Patient Characteristics and Tumor Genomics With Clinical Outcomes Among Patients With Non-Small Cell Lung Cancer Using a Clinicogenomic Database. JAMA. 2019;321(14):1391-9.
27. Okamura R, Kato S, Lee S, Jimenez RE, Sicklick JK, Kurzrock R. ARID1A alterations function as a biomarker for longer progression-free survival after anti-PD-1/PD-L1 immunotherapy. Journal for immunotherapy of cancer. 2020;8 (1) (no pagination)(e000438).
28. Latham A, Srinivasan P, Kemel Y, Shia J, Bandlamudi C, Mandelker D, et al. Microsatellite instability is associated with the presence of Lynch syndrome pan-cancer. Journal of Clinical Oncology. 2019;37(4):286-95.
29. Hechtman JF, Rana S, Middha S, Stadler ZK, Latham A, Benayed R, et al. Retained mismatch repair protein expression occurs in approximately 6% of microsatellite instability-high cancers and is associated with missense mutations in mismatch repair genes. Modern Pathology. 2020;33(5):871-9.
30. Valero C, Lee M, Hoen D, Wang J, Nadeem Z, Patel N, et al. The association between tumor mutational burden and prognosis is dependent on treatment context. Nature genetics. 2021;53(1):11-5.
31. Jimenez-Rodriguez RM, Patil S, Keshinro A, Shia J, Vakiani E, Stadler Z, et al. Quantitative assessment of tumor-infiltrating lymphocytes in mismatch repair proficient colon cancer. Oncoimmunology. 2020;9 (1) (no pagination)(1841948).
32. Middha S, Yaeger R, Shia J, Stadler ZK, King S, Guercio S, et al. Majority of B2M-mutant and -deficient colorectal carcinomas achieve clinical benefit from immune checkpoint inhibitor therapy and are microsatellite instability-high. JCO Precision Oncology. 2019;3(no pagination).

33. Greally M, Chou JF, Chatila WK, Margolis M, Capanu M, Hechtman JF, et al. Clinical and molecular predictors of response to immune checkpoint inhibitors in patients with advanced esophagogastric cancer. Clinical Cancer Research. 2019;25(20):6160-9.
34. Audenet F, Isharwal S, Cha EK, Donoghue MTA, Drill EN, Ostrovnaya I, et al. Clonal relatedness and mutational differences between upper tract and bladder urothelial carcinoma. Clinical Cancer Research. 2019;25(3):967-76.
35. Carlo MI, Khan N, Zehir A, Patil S, Ged Y, Redzematovic A, et al. Comprehensive genomic analysis of metastatic non-clear-cell renal cell carcinoma to identify therapeutic targets. JCO Precision Oncology. 2019;3(no pagination).
36. Abida W, Cheng ML, Armenia J, Middha S, Autio KA, Vargas HA, et al. Analysis of the Prevalence of Microsatellite Instability in Prostate Cancer and Response to Immune Checkpoint Blockade. JAMA oncology. 2019;5(4):471-8.
37. Liu YL, Selenica P, Zhou Q, Iasonos A, Callahan M, Feit NZ, et al. BRCA mutations, homologous DNA repair deficiency, tumor mutational burden, and response to immune checkpoint inhibition in recurrent ovarian cancer. JCO Precision Oncology. 2020;4:665-79.
38. Stasenko M, Feit N, Lee SSK, Shepherd C, Soslow RA, Cadoo KA, et al. Clinical patterns and genomic profiling of recurrent ultra-low risk' endometrial cancer. International Journal of Gynecological Cancer. 2020;30(6):717-23.
39. Albayrak A, Garrido-Castro AC, Giannakis M, Umeton R, Manam MD, Stover EH, et al. Clinical pan-cancer assessment of mismatch repair deficiency using tumor-only, targeted next-generation sequencing. JCO Precision Oncology. 2020;4:1084-97.
40. Doyle LA, Nowak JA, Nathenson MJ, Thornton K, Wagner AJ, Johnson JM, et al. Characteristics of mismatch repair deficiency in sarcomas. Modern Pathology. 2019;32(7):977-87.
41. Christakis AG, Papke DJ, Nowak JA, Yurgelun MB, Agoston AT, Lindeman NI, et al. Targeted Cancer Next-Generation Sequencing as a Primary Screening Tool for Microsatellite Instability and Lynch Syndrome in Upper Gastrointestinal Tract Cancers. Cancer epidemiology, biomarkers & prevention : a publication of the American Association for Cancer Research, cosponsored by the American Society of Preventive Oncology. 2019;28(7):1246-51.
42. Nassar AH, Umeton R, Kim J, Lundgren K, Harshman L, Van Allen EM, et al. Mutational analysis of 472 urothelial carcinoma across grades and anatomic sites. Clinical Cancer Research. 2019;25(8):2458-70.
43. Nikanjam M, Arguello D, Gatalica Z, Swensen J, Barkauskas DA, Kurzrock R. Relationship between protein biomarkers of chemotherapy response and microsatellite status, tumor mutational burden and PD-L1 expression in cancer patients. International Journal of Cancer. 2020;146(11):3087-97.
44. Spizzo G, Puccini A, Xiu J, Goldberg RM, Grothey A, Shields AF, et al. Molecular profile of BRCA-mutated biliary tract cancers. ESMO Open. 2020;5(3).
45. Tokunaga R, Xiu J, Johnston C, Goldberg RM, Philip PA, Seeber A, et al. Molecular profiling of appendiceal adenocarcinoma and comparison with right-sided and left-sided colorectal cancer. Clinical Cancer Research. 2019;25(10):3096-103.
46. Cimic A, Vranic S, Arguello D, Contreras E, Gatalica Z, Swensen J. Molecular Profiling Reveals Limited Targetable Biomarkers in Neuroendocrine Carcinoma of the Cervix. Applied Immunohistochemistry and Molecular Morphology. 2020.
47. Jones NL, Xiu J, Rocconi RP, Herzog TJ, Winer IS. Immune checkpoint expression, microsatellite instability, and mutational burden: Identifying immune biomarker phenotypes in uterine cancer. Gynecologic Oncology. 2020;156(2):393-9.
48. Seeber A, Zimmer K, Kocher F, Puccini A, Xiu J, Nabhan C, et al. Molecular characteristics of BRCA1/2 and PALB2 mutations in pancreatic ductal adenocarcinoma. ESMO Open. 2020;5 (6) (no pagination)(e000942).

49. Stein MK, Pandey M, Xiu J, Tae H, Swensen J, Mittal S, et al. Tumor mutational burden is site specific in non-small-cell lung cancer and is highest in lung adenocarcinoma brain metastases. JCO Precision Oncology. 2019;3(no pagination).
50. Heeke AL, Xiu J, Elliott A, Korn WM, Lynce F, Pohlmann PR, et al. Actionable co-alterations in breast tumors with pathogenic mutations in the homologous recombination DNA damage repair pathway. Breast Cancer Research and Treatment. 2020;184(2):265-75.
51. Lorenzi M, Amonkar M, Zhang J, Mehta S, Liaw KL. Epidemiology of Microsatellite Instability High (MSI-H) and Deficient Mismatch Repair (dMMR) in Solid Tumors: A Structured Literature Review. Journal of Oncology. 2020;2020 (no pagination)(1807929).
52. Jin J, Shi Y, Zhang S, Yang S. PIK3CA mutation and clinicopathological features of colorectal cancer: a systematic review and Meta-Analysis. Acta Oncologica. 2020;59(1):66-74.
53. Ryan NAJ, Glaire MA, Blake D, Cabrera-Dandy M, Evans DG, Crosbie EJ. The proportion of endometrial cancers associated with Lynch syndrome: a systematic review of the literature and meta-analysis. Genetics in Medicine. 2019;21(10):2167-80.
54. Kato S, Hayashi T, Suehara Y, Hamanoue H, Yamanaka S, Ichikawa Y, et al. Multicenter experience with large panel next-generation sequencing in patients with advanced solid cancers in Japan. Japanese journal of clinical oncology. 2019;49(2):174-82.
55. Kahn RM, Gordhandas S, Maddy BP, Baltich Nelson B, Askin G, Christos PJ, et al. Universal endometrial cancer tumor typing: How much has immunohistochemistry, microsatellite instability, and MLH1 methylation improved the diagnosis of Lynch syndrome across the population? Cancer. 2019;125(18):3172-83.
56. Kunnackal John G, Das Villgran V, Caufield-Noll C, Giardiello F. Worldwide variation in lynch syndrome screening: case for universal screening in low colorectal cancer prevalence areas. Familial Cancer. 2020.
57. Wang B, Li F, Zhou X, Ma Y, Fu W. Is microsatellite instability-high really a favorable prognostic factor for advanced colorectal cancer? A meta-analysis. World Journal of Surgical Oncology. 2019;17 (1) (no pagination)(169).
58. Deng Z, Qin Y, Wang J, Wang G, Lang X, Jiang J, et al. Prognostic and predictive role of DNA mismatch repair status in stage II-III colorectal cancer: A systematic review and meta-analysis. Clinical Genetics. 2020;97(1):25-38.
59. Fountzilas E, Kotoula V, Pentheroudakis G, Manousou K, Polychronidou G, Vrettou E, et al. Prognostic implications of mismatch repair deficiency in patients with nonmetastatic colorectal and endometrial cancer. ESMO Open. 2019;4 (2) (no pagination)(000474).
60. O'Connell E, Reynolds IS, McNamara DA, Prehn JHM, Burke JP. Microsatellite instability and response to neoadjuvant chemoradiotherapy in rectal cancer: A systematic review and meta-analysis. Surgical Oncology. 2020;34:57-62.
61. Meillan N, Vernerey D, Lefevre JH, Manceau G, Svrcek M, Augustin J, et al. Mismatch Repair System Deficiency Is Associated With Response to Neoadjuvant Chemoradiation in Locally Advanced Rectal Cancer. International Journal of Radiation Oncology Biology Physics. 2019;105(4):824-33.
62. Willis C, Fiander M, Tran D, Korytowsky B, Thomas JM, Calderon F, et al. Tumor mutational burden in lung cancer: A systematic literature review. Oncotarget. 2019;10(61):6604-22.
63. Zhu J, Zhang T, Li J, Lin J, Liang W, Huang W, et al. Association between tumor mutation burden (TMB) and outcomes of cancer patients treated with PD-1/ PD-L1 inhibitions: A meta-analysis. Frontiers in Pharmacology. 2019;10 (JUN) (no pagination)(673).
64. Salem ME, Yin J, Goldberg RM, Pederson LD, Wolmark N, Alberts SR, et al. Evaluation of the change of outcomes over a 10-year period in patients with stage III colon cancer: pooled analysis of 6501 patients treated with fluorouracil, leucovorin, and oxaliplatin in the ACCENT database. Annals of Oncology. 2020;31(4):480-6.
65. Taieb J, Shi Q, Pederson L, Alberts S, Wolmark N, Van Cutsem E, et al. Prognosis of microsatellite instability and/or mismatch repair deficiency stage III colon cancer patients after disease

recurrence following adjuvant treatment: Results of an ACCENT pooled analysis of seven studies. Annals of Oncology. 2019;30(9):1466-71.
66. Sinicrope FA, Chakrabarti S, Laurent-Puig P, Huebner L, Smyrk TC, Tabernero J, et al. Prognostic variables in low and high risk stage III colon cancers treated in two adjuvant chemotherapy trials. European Journal of Cancer. 2021;144:101-12.
67. Pietrantonio F, Miceli R, Raimondi A, Kim YW, Kang WK, Langley RE, et al. Individual patient data meta-analysis of the value of microsatellite instability as a biomarker in gastric cancer. Journal of Clinical Oncology. 2019;37(35):3392-400.
68. Choi YY, Kim H, Shin SJ, Kim HY, Lee J, Yang HK, et al. Microsatellite Instability and Programmed Cell Death-Ligand 1 Expression in Stage II/III Gastric Cancer: Post Hoc Analysis of the CLASSIC Randomized Controlled study. Annals of Surgery. 2019;270(2):309-16.
69. Barroso-Sousa R, Jain E, Cohen O, Kim D, Buendia-Buendia J, Winer E, et al. Prevalence and mutational determinants of high tumor mutation burden in breast cancer. Annals of Oncology. 2020;31(3):387-94.
